# Supplementary material for: Putting your money where your self is: Connecting dimensions of closeness and theories of personal identity
Source: PLoS One. 2020 Feb 12;15(2):e0228271. doi: 10.1371/journal.pone.0228271 (PMC7015397; doi:10.1371/journal.pone.0228271)
Supplement: S1 Supporting Information — Study materials, supporting analyses, and qualitative coding scheme. (PDF) [file pone.0228271.s001.pdf]

S1 Supporting Information: Supporting Material for "Putting your Money Where your Self is"

Jan K. Woike*
Max Planck Institute for Human Development, Center for Adaptive Rationality (ARC), Lentzeallee 94, 14195 Berlin, Germany

Philip Collard
University of Bristol, School of Psychological Science, Bristol, UK

Bruce Hood
University of Bristol, School of Psychological Science, Bristol, UK

*Corresponding author, E-mail: woike@mpib-berlin.mpg.de

Contents

1. Surveys presented in the manuscript **4**
1.1. Survey Study 1 . . . 4
1.1.1. Introduction . . . 4
1.1.2. Q1 — Money split . . . 4
1.1.3. Q2 — Explanation . . . 4
1.1.4. Q3–Q8 — Post-Questionnaire . . . 6
1.1.5. Q9–Q13 — Importance for identity . . . 6
1.1.6. Q14–Q18 — Importance for survival . . . 6
1.2. Survey Study 2 . . . 7
1.2.1. Introduction . . . 7
1.2.2. QII1 — Decision . . . 8
1.2.3. QII2 — Decision difficulty . . . 8
1.2.4. QII3–QII8 — Post-Questionnaire . . . 9
1.2.5. QII9–QII32 — Importance for identity . . . 10
1.2.6. QII33–QII37 — Importance for survival . . . 11
1.2.7. QII38–QII47 — Reductionism and Self-evaluation items (exploratory) . 11
1.3 Notes on the chosen scenario . . . 12

2. Additional data analysis **14**
2.1. Study 1 . . . 14
2.1.1. Detailed pyramid plot of allocation decisions . . . 14

2.1.2. Stability of main effects across different concentrations of dimensions . . 16
2.1.3. Full ANOVA tables 16
2.1.4. Being two people and split psychology and memory 22
2.1.5. Identification with continuers and distribution of memory and psychology 22
2.1.6. Intercorrelations of postquestionnaire responses 25
2.1.7. Weighting of dimensions and monetary allocation: exploratory findings . 25
2.1.8. Body Importance and allocations: Exploratory regression models 28
2.1.9. Cluster analyses of postquestionnaire items: Explicit weighting of dimensions 33
2.1.10. Cluster analyses of postquestionnaire items: Re-identification after fission 35
2.2. Study 2 38
2.2.1. Full ANOVA Tables 38
2.2.2. Decision difficulty and split psychology and memory 45
2.2.3. Inter-correlations of postquestionnaire responses 45
2.2.4. Post-questionnaire differences between studies 47
2.2.5. Order effects for survival items 48
2.2.6. Reductionist and non-reductionist belief among participants 49
2.2.7. Cluster analyses of postquestionnaire items: Explicit weighting of dimensions 50
2.2.8. Cluster analyses of postquestionnaire items: Re-identification after fission 51
2.2.9. Percentage of traits retained and postquestionnaire responses 54
2.3. Qualitative Analysis in Study 1: Reasons given for allocating the money 57
2.3.1. Confirmatory evidence 57
2.3.2. Alternative interpretations 61
2.3.3. Conclusion 63

References **64**

List of Figures

A Scenario condition table in Study 1 5
B Slider element for money allocation 5
C Scenario condition table in Study 2 8
D Radio boxes for inheritance decision 9
E Slider element for decision difficulty 9
F Pyramid plot of monetary allocations (Study 1) 15
G Allocation based on location of psychology and memory (Study 1) 17
H Two-people response based on distribution of memory and psychology (Study 1) 22
I Scatterplots of identification with continuers A and B (Study 1) 24
J Difference in allocation split by attribute importance (Study 1) 29
K Value of body continuity based on judged importance (Study 1) 31
L Cluster centers for explicit weightings (Study 1) 34

M Cluster centers for postquestionnaire items (Study 1) 36
N Decision difficulty based on split of memory and psychology (Study 2) 45
O Cluster centers for explicit weightings (Study 2) 52
P Cluster centers for postquestionnaire items (Study 2) 53
Q Identification based on percentage of dimensions retained (Study 2) 55
R Survival and sameness based on percentage of dimensions retained (Study 2) . 56
S Being two people and non-existence based on percentage of dimensions retained (Study 2) 57

List of Tables

A ANOVA results for monetary allocation (Study 1) 18
B ANOVA for identification with Person A (Study 1) 19
C ANOVA for identification with Person B (Study 1) 20
D ANOVA for identification with both continuers (Study 1) 21
E Correlations and descriptives of explicit weightings (Study 1) 26
F Correlations and descriptives of postquestionnaire items (Study 1) 27
G Tobit regression results (Study 1) 32
H Cluster differences (Study 1) 38
I ANOVA for inheritance decision difficulty weighted by direction (Study 2) . . 39
J ANOVA for inheritance decision difficulty (Study 2) 40
K ANOVA results for identification with Person A (Study 2) 41
L ANOVA results for identification with Person B (Study 2) 42
M ANOVA for identification with both continuers (Study 2) 43
N ANOVA for non-existence after fission (Study 2) 44
O Correlations between explicit weightings (Study 2) 46
P Correlations between weightings for identity and survival (Study 2) 47
Q Test of cueing effects (Study 1 and 2) 48
R Test of order effects for surival weightings (Study 1 and 2) 49
S Descriptives for questions about body–mind relationship (Study 2) 50
T Cluster differences (Study 2) 54
U Distribution of justification types in open answers (Study 1) 59
V Distribution of unusual justification types in open answers (Study 1) 61

1. Surveys presented in the manuscript

1.1. Survey Study 1

The questionnaire was constructed and presented with Qualtrics.

1.1.1. Introduction. The following questions are part of a study about identity and survival. The chosen scenarios are mostly science-fiction scenarios. Please try to imagine nonetheless that the described events are possible. We are genuinely interested in how you would judge the situations if they actually occurred.

In the future, hyperspace travel has become normal. You enter hyperspace to travel large distances and leave it at your new destination. Unfortunately, the technology still has some problems. One rare incident occurs, while you are traveling from one planet to another planet: for a brief moment, the present universe overlaps with a parallel universe. Your travel agency contacts you while you are still in hyperspace and informs you that due to the overlap it has been calculated that, unfortunately, not one but two people will leave the hyperspace at your target destination: person A and person B, while you will no longer exist in your present state:

1. One of the two persons has your exact body and appearance, the other person has the body and appearance of a randomly chosen person of the same age and gender.
2. One of the two persons has the same personality and psychology as you, the other person has the personality and psychology of a randomly chosen person of the same age and gender.
3. One of the two persons has the same memory and knowledge as you, the other person has the memory and knowledge of a randomly chosen person of the same age and gender.
4. One of the two persons has the same friends as you, the other person has the friends of a randomly chosen person of the same age and gender (these friends have been impacted by the incident as well and are true friends of this person).
5. One of the two persons has the same possessions as you, the other person has the possessions of a randomly chosen person of the same age and gender (these possessions have been impacted by the incident as well and are true and uncontested possessions of this person).

The diagram below shows, which person is in which condition after the exit from hyperspace. [*A variant of Figure A is shown to participant*].

1.1.2. Q1 — Money split. The travel agency's insurance company is willing to pay 100 000 $ to compensate you for the problems caused by the incident. Fortunately, they can contact you before you leave hyperspace. You have to decide now, how to distribute the money between the two people that will exist (at your place) after you leave hyperspace. (The values you choose must add up to 100 000). [*The slider element shown in Figure B is used as input element.*]

1.1.3. Q2 — Explanation. Please explain your answer (briefly)

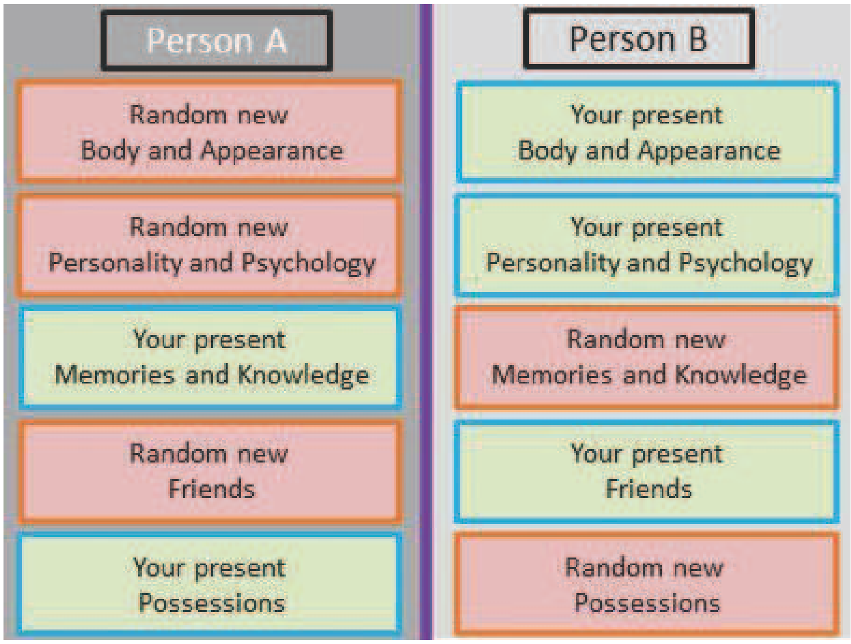

Figure A. Figure shown to participants that characterizes their scenario condition: the five components exist in their present form and in randomly replaced form and are assigned to the two continuers, so that one of each version is is given to the pair. The green boxes represent present versions and the red boxes random versions, with the stack of five boxes under each person defining their respective configuration. There are 32 images that define the 32 scenarios.

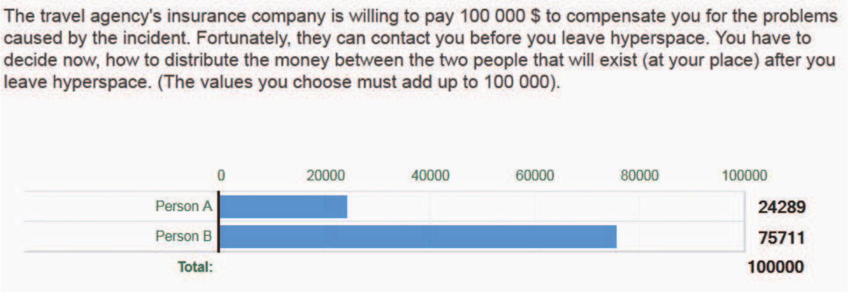

Figure B. Slider element (Qualtrics) used to input the insurance sum allocation: Sliders start in an unassigned position, and the sum is constrained to stay below 100,000. A total of 100,000 is necessary for proceeding.

1.1.4. Q3–Q8 — Post-Questionnaire. Now the incident has occurred and two persons leave hyperspace. Please click on the scale position that corresponds best to the degree you agree with the statement.

- You have survived the incident.
- You are the same person as before the incident.
- You are Person A after the incident.
- You are Person B after the incident.
- You do not exist after the incident.
- You are two people after the incident.

Scale: Strongly Disagree – Disagree – Neither Agree nor Disagree – Agree – Strongly Agree (1-5, continuous)

1.1.5. Q9–Q13 — Importance for identity. How important are the following aspects for you when it comes to determining identity between two people?

- Body and Appearance
- Personality and Psychology
- Memory and Knowledge
- Friends
- Possessions

Scale: Not at all important– Extremely important (0-10, continuous)

1.1.6. Q14–Q18 — Importance for survival. How important are the following aspects for you when it comes to determining the survival of a person?

- Body and Appearance
- Personality and Psychology
- Memory and Knowledge
- Friends
- Possessions

Scale: Not at all important– Extremely important (0-10, continuous)

1.2. Survey Study 2

The questionnaire was constructed and presented with Qualtrics. Elements were injected via JavaScript using the libraries "Bootstrap" (version 3.3.6), "JQuery" (version 1.11) and "JQuery UI" (version 1.11.4). The use of JavaScript necessitated the avoidance of Qualtrics new Jiffy-Engine by adding "Q_JFE=0" to the generated link.

1.2.1. Introduction. The following questions are part of a study about identity and survival. The chosen scenarios are mostly science-fiction scenarios. Please try to imagine nonetheless that the described events are possible. We are genuinely interested in how you would judge the situations if they actually occurred.

[*Shown on new page.*]

In the future, hyperspace travel has become normal. Long-distance travelers enter hyperspace, travel from planet to planet and leave it at their destination. Travel pods are built for single travelers, there is no pilot on board.

Unfortunately, the technology still has some problems. One rare incident occurs in the middle of your journey: for a brief moment, the present universe overlaps with a parallel universe.

Your travel agency contacts you while you are still in hyperspace and informs you that due to the overlap it has been calculated that (unfortunately) not one but two people will leave the pod at your target destination: person A and person B (and no-one else). The following is true for person A and person B:

[*The enumeration was generated in JavaScript and injected into the page. The five statements were ordered randomly.*]

1. One of the two persons has the same friends as you, the other person has the friends of a randomly chosen person of the same age and gender (these friends have been impacted by the incident as well and are true friends of this person).

2. One of the two persons has the same possessions as you, the other person has the possessions of a randomly chosen person of the same age and gender (these possessions have been impacted by the incident as well and are true and uncontested possessions of this person).

3. One of the two persons has the same personality and psychology as you, the other person has the personality and psychology of a randomly chosen person of the same age and gender.

4. One of the two persons has your exact body and appearance, the other person has the body and appearance of a randomly chosen person of the same age and gender.

5. One of the two persons has the same memory and knowledge as you, the other person has the memory and knowledge of a randomly chosen person of the same age and gender.

The table below shows, which person is in which condition after the exit from hyperspace. [*A variant Figure C is shown to participant*].

[*The table was generated in JavaScript and injected into the page. The order corresponded to the order in the descriptions.*]

	Person A	Person B
**Friends**	Your present Friends	Random new Friends
**Possessions**	Random new Possessions	Your present Possessions
**Personality and Psychology**	Random new Personality and Psychology	Your present Personality and Psychology
**Body and Appearance**	Your present Body and Appearance	Random new Body and Appearance
**Memories and Knowledge**	Random new Memories and Knowledge	Your present Memories and Knowledge

Figure C. Table shown to participants that characterizes their scenario condition. The red boxes represent present new random versions and the blue boxes original versions, with the stack of five boxes under each person defining their respective configuration. As the order is randomized, there are $120 \cdot 32 = 3840$ possible versions of this table. The 32 different scenarios were counterbalanced across participants.

Both persons will be compensated for the incident.

1.2.2. QII1 — Decision. At the same time, the travel agency informs you that a distant relative of yours has died and that you have been chosen as the sole heir of the relative's estate. The inheritance cannot be split up into smaller parts and it cannot be sold or divided by other means.

Due to the circumstances of the incident, you are asked to decide, which of the persons leaving the pod at your destination should be declared heir, person A or person B (it has to be either A or B, the other person cannot receive any part of the inheritance at any point in time).

[*A customized version of radio boxes shown in Figure D was used as input element.*]

1.2.3. QII2 — Decision difficulty. [*shown after the decision on a new page*]

How difficult was it for you to decide who should be declared the heir?

[*The input element is shown in Figure E.*]

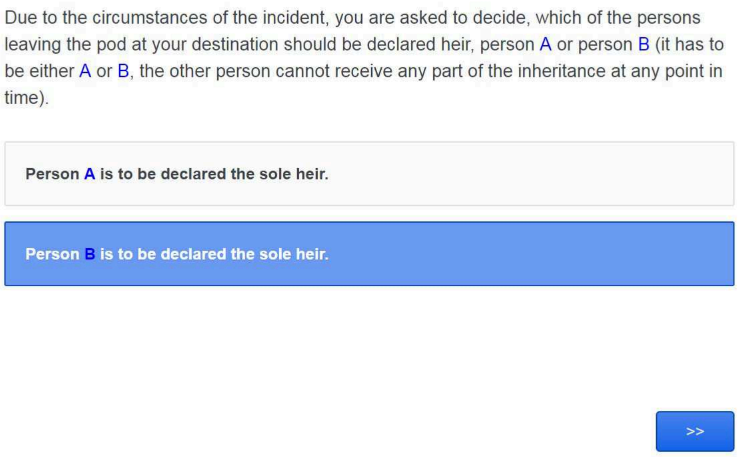

Figure D. Radio boxes to enter the decision between heirs. A click on a box turned the box dark blue, the decision had to be confirmed by clicking the “»”-button.

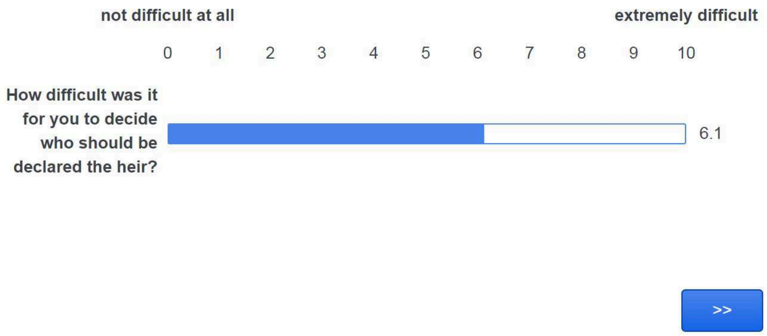

Figure E. Slider element (Qualtrics) used to input the decision difficulty. The sliders starts in an unassigned position.

1.2.4. QII3–QII8 — Post-Questionnaire. [*shown on new page.*]

Now the incident has occurred and two persons leave hyperspace. Please click on the scale position that corresponds best to the degree you agree with the statement. (*The table below the questions is the same table you have seen before*).

[*Items were ordered randomly*]

- You have survived the incident.
- You are the same person as before the incident.
- You are Person A after the incident.
- You are Person B after the incident.

- You do not exist after the incident.
- You are two people after the incident.

Scale: Strongly Disagree – Disagree – Neither Agree nor Disagree – Agree – Strongly Agree (0-5, continuous) [*Below the questions the table was shown again*]

1.2.5. QII9–QII32 — Importance for identity. [*Shown on new page.*] How important are the following aspects for you when it comes to determining identity between two people?

[*The eight items below were shown in random order.*]

- Body
- Appearance
- Personality
- Psychology
- Memory
- Knowledge
- Friends
- Possessions

[*Six of the 16 items were randomly selected (balanced across participants) and presented in random order.*]

- Mind
- Brain
- Nationality
- Gender
- Moral values
- Virtues and vices
- Philosophy of life
- Religion
- Loved ones
- Family

- Relationships with other people
- Membership in groups
- Private roles
- Professional roles
- Profession/Job
- Colleagues

Scale: Not at all important– Extremely important (0-10, continuous)

1.2.6. QII33–QII37 — Importance for survival. [*Shown on new page.*]

How important are the following aspects for you when it comes to determining the survival of a person?

[*same order as above*]

- Body and Appearance
- Personality and Psychology
- Memory and Knowledge
- Friends
- Possessions

Scale: Not at all important– Extremely important (0-10, continuous)

1.2.7. QII38–QII47 — Reductionism and Self-evaluation items (exploratory). [*Shown on new page.*]

Please click on the scale position that corresponds best to the degree you agree with each statement.

[*Items were presented in random order*]

- Nothing in the mind occurs that does not originate in the body.
- Everything that we think or feel can be reduced to activations of neurons in our body.
- Ghosts truly exist in this world.
- At least in theory psychology can be reduced to physics.
- Body and mind are independent to some relevant degree.
- Humans have souls that are separate from their bodies.

- In the future it may be possible to overcome death by uploading your mind to a computer.
- I like the current state of my body and appearance.
- I like the current state of my memories and knowledge.
- I would like to be somebody else.

Scale: Strongly Disagree – Disagree – Neither Agree nor Disagree – Agree – Strongly Agree (1-5, continuous)

1.3 Notes on the chosen scenario

Here, we extend the discussion of possible challenges and objections to the studies' main scenario (section 4.4) in the manuscript, discussing the proper role of the brain, morality, and causal connections.

For McMahan (2002, p. 68), future-oriented self-concern should be based on the continuity of brain areas that support consciousness and mental activity.In contrast, Olson (2003) considered the brain to be one organ among others and argued for identifying the person with the body. Some will argue that our scenario cannot overcome its dualist overtones: Integrating memory, psychology, and body in "the brain" might yield a more convincing and realistic criterion for determining identity. What speaks in favor of this objection is the relatively high importance assigned to "brain" in Study 2.

Ordinarily though, any concern for the brain is an instrumental concern:D. Shoemaker (2014) argued: "It may be rational for me to anticipate only the experiences of my biological continuers, for instance, but it won't be *in virtue* of my biological continuity with them that it's rational to do so; rather it seems rational only in virtue of the psychological relations they are expected to bear to me.". A hypothetical replacement of the brain by a functionally equivalent artificial replacement without a break in causal continuity would for most people not be equivalent to a death (Nozick, 1981; Williams, 1970). Identifying psychological states as a property of individuals (or even a "state" of individual brains) is criticized in philosophy (Clark, 1997; Putnam, 1975) and, the brain itself is the basis for a multitude of capabilities, many of them separable in principle. The idea of a "nefarious neuroscientist" (Matheson, 2014) targeting and changing specific psychological functions in isolation would still be compatible with a reductionist position.

The empirical evidence shows that even when people accept the singular importance of the brain (Chen, Urminsky, & Bartels, 2016; Gottfried, Gelman, & Schultz, 1999) participants faced with a brain transplant scenario do not view the replacement of brain matter as identity-changing when it is not accompanied by changes in values or behavior (Strohminger & Nichols, 2014). Blok, Newman, Behr, and Rips (2001) showed that a computer copy with preserved memories was still rated as closer to the original person than a transplanted brain with altered memories. Nichols and Bruno (2010) directly demonstrated that observers judge a person to be preserved in a brain transplant if and only if memories are preserved. Our own data make it

clear that only a minority of participants strongly endorsed reductionist positions (see section 2.2.6.).

Evidence from several studies considering real-world personal transformations has indicated that identity judgments are most heavily influenced by changes or steadfastness in moral values (Strohminger & Nichols, 2014). Changes in morality were judged to be more relevant than changes in (nonmoral) personality attributes or memory. In a similar vein, Strohminger and Nichols (2015) reported that changes in morality in patients with neurodegenerative diseases strongly determined changes in perceived identity. Nunner-Winkler (2015) reported on a study asking participants which changes would lead them to see themselves as a different person. Ideas about right and wrong and gender identity were considered to be quite important; appearance and money were considered less relevant (although some participants rated looks to be important, consistent with our distributional results). On the other hand, moral categories were named less frequently than psychological and bodily categories in the study by Berniūnas and Dranseika (2016). Morality is different from other dimensions due to the fact that one's morals cannot exist in a social vacuum; moral consensus is central for coordination, affiliation, and conflict resolution. Morality stands in complex relations to beliefs, values, behaviors and communities, and the distinction between moral and nonmoral traits is somewhat ambiguous (e.g., conscientiousness was considered as a moral trait rather than a personality factor in some studies). Most people also seem to have an inflated belief of their own morality (Newman, De Freitas, & Knobe, 2015). Some argue that morality is inconceivable without personal identity (Mills, 1993; Parfit, 1984; D. W. Shoemaker, 2007).

Morality also depends on memory in nontrivial ways. Some of the scenarios used in this stream of research even involved the complete loss of the moral faculty, which could have been seen to have a strong effect on other dimensions of the self. If this is the case, the relevance of morality for personal identity might lie in these possibly disruptive consequences of changing one's morals in relation to one's environment and not because of its self-defining importance. Evidence for this interpretation is found in two studies demonstrating that changes in widely shared (and therefore less unique to the individual) moral values are considered to lead to more changes to the person than changes in controversial moral beliefs (Heiphetz, Strohminger, & Young, 2017; Heiphetz, Strohminger, Gelman, & Young, n.d.). For controversial moral beliefs, which might be considered more definitive of a person's self, the effect was weaker than for memory. In our scenarios, dimensions are replaced through random sampling from the participant's reference population, which is a different operationalization of change than loss or explicitly defined transformations. Heiphetz et al. (n.d.) showed how the perceived change was mediated by perceived disruptions of friendships; it is therefore difficult to predict the role morality would play in our scenario.

Critics of our scenario might further object that our random collage of features in the two continuers destroys the causal connection between past and present states necessary for identity (Matthews, 2000; Parfit, 1984). Preschool children already individuate objects and persons spatiotemporally (Gutheil, Gelman, Klein, Michos, & Kelaita, 2008; Wagner & Carey, 2003) and, following Sagi and Rips (2014), causal histories receive special attention in linguis-

tic disambiguation in discourse. In all our scenarios (except the two extreme cases with exact duplicates), change in characteristics was induced by an accident, an unusual life event that disrupts spatiotemporal continuity. This fact might strengthen impressions that identity is not preserved. According to data reported by Nunner-Winkler (2015), for example, participants regarded changes in attitudes or beliefs that were due to normal life experiences as nonconsequential for identity judgments—as opposed to changes induced by brainwashing, severe medical conditions, or accidents. Therefore, the nature of the transformation might play a role in our participants' judgments.

Accident or illness can severely transform both memory and personality. Kitcher (1979) considered the possibility that total amnesia may violate the psychological "laws" that typically govern the class of persons; our scenarios likewise violate those laws. We implicitly assume, however, that the resulting persons are indistinguishable from persons who lived through the memories; thus, the quasi-character of memories should not disqualify the continuers from survival (Campbell, 2005; Kolak & Martin, 1987). Although the abruptness and symmetry of the original person's transformation prevents the application of spatiotemporal continuation criteria, participants might still construct "fictive causal histories" (Fields, 2012) to assess which of the two continuers might have the better chance of being the result of changes within an ordinary life.

2. Additional data analysis

Most analyses were conducted using IBM SPSS 20.0 (IBM Corp., 2011). Confidence intervals were calculated using the Exploratory Software for Confidence Intervals (ESCI, Cumming, 2014). Other software is indicated where it applies. Diagrams were created in LaTeX using PSTricks (Van Zandt, 2003).

2.1. Study 1

2.1.1. Detailed pyramid plot of allocation decisions. Figure F shows a detailed pyramid plot of allocation decisions. Depicted are allocations to Person B (which are the complement to the allocations to Person A, as both have to add up to USD 100,000) that range over the full spectrum from USD 0 to USD 100,000. The diagram is vertically split for cases, in which original psychology and original memory are united in one of the two persons and cases in which they are split between the two continuers. It is also clearly visible that many participants chose to split the money close to evenly.

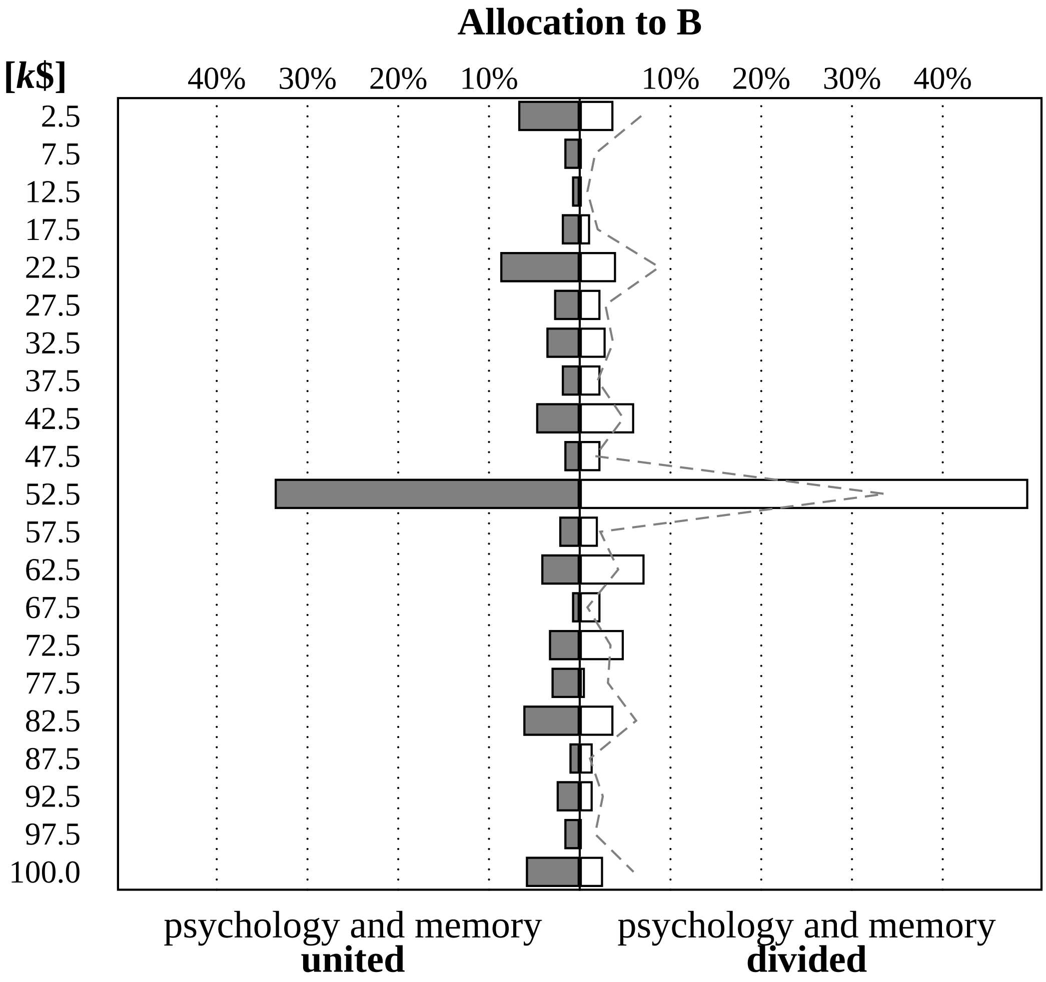

Figure F. Pyramid plot of allocations to Person B (in thousands of dollar) in Study 1: The relative frequencies for scenarios with memory and psychology united within one continuer (n=354) are shown on the left side, the relative frequencies for the components divided between continuers (n=350) are shown on the right side. For each of 21 intervals the percentage of allocations is plotted for each subset of scenarios (intervals are 5,000 $ intervals with the included upper limit shown as value on the y-axis, the two extreme intervals are 2,500 $ wide). The dashed line on the divided side mirrors the percentages on the united side to allow for side comparisons.

2.1.2. Stability of main effects across different concentrations of dimensions. To further explore the stability of the ANOVA results for explaining financial allocations that we found (two main effects and no interactions), we separated the conditions into groups that shared the same percentage of original attributes maintained by Person B. Ignoring cases for which Person B maintains none or all of the attributes, four situations are investigated separately, in which B retains between one (20%) and four (80%) of the original attributes.

Given the absence of significant interactions, substantive main effects for memory and psychology should be observable independent of the distribution of the other three attributes. Therefore we plotted the average allocation to Person B as a function of presence of the original memory and psychology. The cases where none or all five attributes are given to Person B are not relevant here, as there is no variation of either factor in this condition, while all four combinations of presence and absence of the two personal attributes can only be observed in the conditions with two (40%) and three (60%) of attributes assigned to person B. Figure G shows the four resulting graphs in a panel. Given the between-subject nature of the design, each graph presents results for a unique subset of participants. In all conditions there is a discernible difference in allocations between scenario variants with presence and absence of either dimension. The size of allocations in cases where only one of the two dimensions is present does not seem to depend on which of the two B retains (which corresponds to the similarity in effect sizes with comparable variance).

2.1.3. Full ANOVA tables. The full results for the four ANOVAs mentioned in the main text are presented in TabÃűe A for the monetary allocation, in Table B for the identification with Person A, in Table C for the identification with Person B, and in Table D for the identification with both continuers simultaneously.

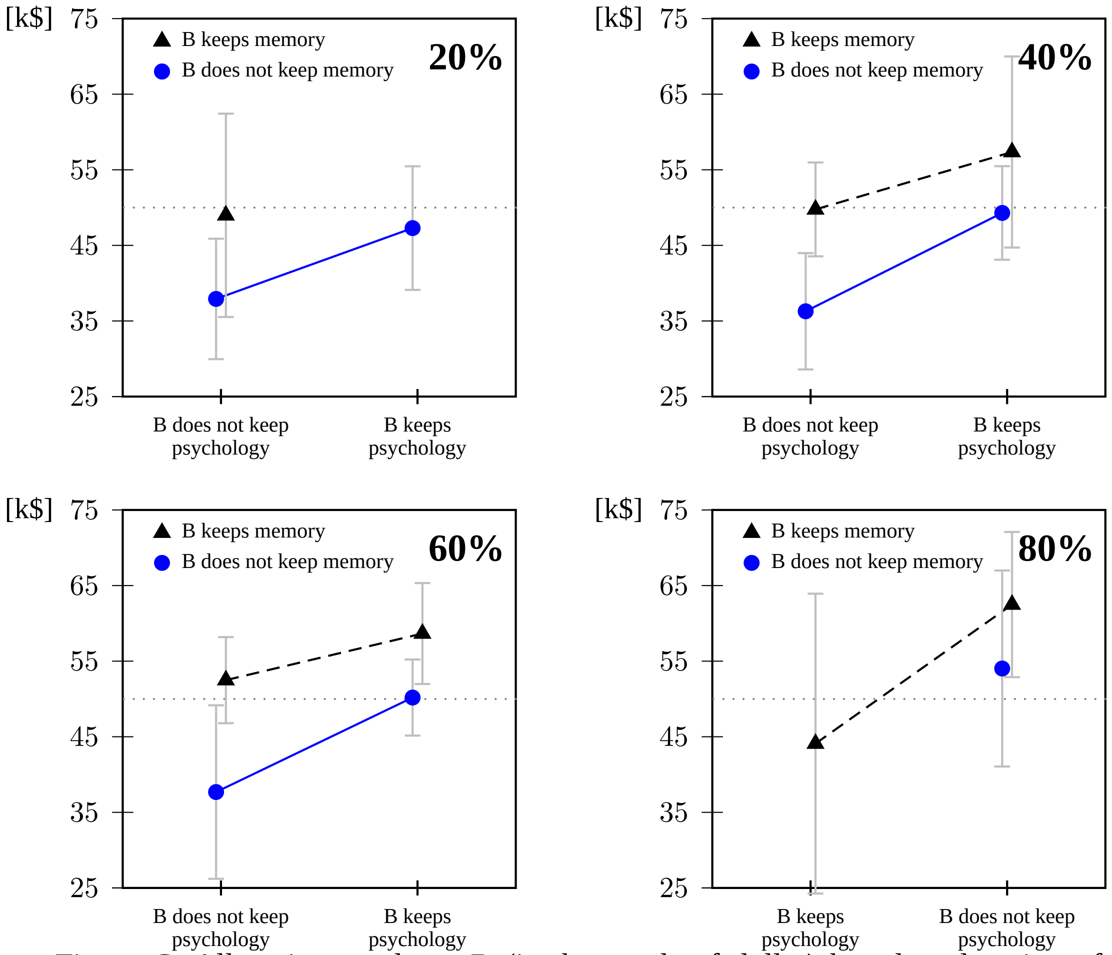

Figure G. Allocation to player B (in thousands of dollar) based on location of psychology and memory and percentage of attributes inherited by continuer B (Study 1): separate graphs summarize scenarios, in which continuer B inherits between one (20%) and four (80%) of the original components. In each graph, allocations are shown for each possible division of the memory and psychology component. Note that B cannot receive both components in the 20% condition and A cannot receive both components in the 80% condition. Whiskers show the 99% confidence intervals around the means.

Table A

ANOVA results for allocation to Person B as dependent variable in Study 1 (complete five-factorial design, N = 704)

Source	df	Mean square	F	*p*	Partial η^2
Intercept	1	1,672,592,838,417.1	3523.63	<.001	0.84
Body	1	172,039,134.5	0.36	.55	0.00
Psychology	1	21,364,023,540.4	45.01	<.001	0.06
Memory	1	21,454,301,417.7	45.20	<.001	0.06
Friends	1	213,581,425.6	0.45	.50	0.00
Possessions	1	117,203,874.2	0.25	.62	0.00
Body * Psychology	1	728,891,783.7	1.54	.22	0.00
Body * Memory	1	209,546.9	0.00	.98	0.00
Body * Friends	1	134,184,320.0	0.28	.60	0.00
Body * Possessions	1	58,781,736.9	0.12	.73	0.00
Psychology * Memory	1	349,246,942.0	0.74	.39	0.00
Psychology * Friends	1	99,275,390.8	0.21	.65	0.00
Psychology * Possessions	1	146,652,492.9	0.31	.58	0.00
Memory * Friends	1	2,929,968.1	0.01	.94	0.00
Memory * Possessions	1	17,603,198.1	0.04	.85	0.00
Friends * Possessions	1	1,641,054,047.3	3.46	.06	0.01
Body * Psychology * Memory	1	35,034,378.4	0.07	.79	0.00
Body * Psychology * Friends	1	263,195,935.1	0.55	.46	0.00
Body * Psychology * Possessions	1	220,764,710.7	0.47	.50	0.00
Body * Memory * Friends	1	42,847,043.7	0.09	.76	0.00
Body * Memory * Possessions	1	1,361,374,594.1	2.87	.09	0.00
Body * Friends * Possessions	1	313,937,995.6	0.66	.42	0.00
Psychology * Memory * Friends	1	92,592,035.4	0.20	.66	0.00
Psychology * Memory * Possessions	1	31,214,164.0	0.07	.80	0.00
Psychology * Friends * Possessions	1	1,555,328.1	0.00	.95	0.00
Memory * Friends * Possessions	1	453,268,655.0	0.95	.33	0.00
Body * Psychology * Memory * Friends	1	851,471,535.0	1.79	.18	0.00
Body * Psychology * Memory * Possessions	1	437,555,664.0	0.92	.34	0.00
Body * Psychology * Friends * Possessions	1	8,699,930.1	0.02	.89	0.00
Body * Memory * Friends * Possessions	1	679,020,253.2	1.43	.23	0.00
Psychology * Memory * Friends * Possessions	1	106,558,023.6	0.22	.64	0.00
Body * Psychology * Memory * Friends * Possessions	1	9,089,960.0	0.02	.89	0.00
Error	672	474,678,558.7			
Total	704				

Table B

ANOVA results for identification with Person A as dependent variable (complete five-factorial design, N=692)

Source	df	Mean Square	F	Sign.	Partial η^2
Intercept	1	5429.17	4799.92	<.001	0.88
Body	1	0.57	0.51	.48	0.00
Psychology	1	79.47	70.26	<.001	0.10
Memory	1	86.53	76.50	<.001	0.10
Friends	1	1.00	0.88	.35	0.00
Possessions	1	0.06	0.05	.82	0.00
Body * Psychology	1	0.13	0.12	.73	0.00
Body * Memory	1	0.75	0.66	.42	0.00
Body * Friends	1	4.13	3.65	.06	0.01
Body * Possessions	1	0.00	0.00	.98	0.00
Psychology * Memory	1	1.04	0.92	.34	0.00
Psychology * Friends	1	0.65	0.57	.45	0.00
Psychology * Possessions	1	1.83	1.62	.20	0.00
Memory * Friends	1	1.43	1.26	.26	0.00
Memory * Possessions	1	0.04	0.03	.86	0.00
Friends * Possessions	1	0.14	0.13	.72	0.00
Body * Psychology * Memory	1	0.11	0.10	.76	0.00
Body * Psychology * Friends	1	0.05	0.04	.84	0.00
Body * Psychology * Possessions	1	0.04	0.03	.86	0.00
Body * Memory * Friends	1	0.10	0.09	.77	0.00
Body * Memory * Possessions	1	0.88	0.78	.38	0.00
Body * Friends * Possessions	1	0.17	0.15	.70	0.00
Psychology * Memory * Friends	1	0.01	0.01	.94	0.00
Psychology * Memory * Possessions	1	1.09	0.97	.33	0.00
Psychology * Friends * Possessions	1	0.08	0.07	.79	0.00
Memory * Friends * Possessions	1	1.68	1.49	.22	0.00
Body * Psychology * Memory * Friends	1	2.44	2.15	.14	0.00
Body * Psychology * Memory * Possessions	1	0.33	0.29	.59	0.00
Body * Psychology * Friends * Possessions	1	1.06	0.94	.33	0.00
Body * Memory * Friends * Possessions	1	0.01	0.01	.94	0.00
Psychology * Memory * Friends * Possessions	1	0.29	0.26	.61	0.00
Body * Psychology * Memory * Friends * Possessions	1	4.11	3.63	.06	0.01
Error	660	1.13			
Total	692				

Table C

ANOVA results for identification with Person B as dependent variable (complete five-factorial design, N=694)

Source	df	Mean Square	F	Sign.	Partial η^2
Intercept	1	4851.24	4226.41	<.001	0.87
Body	1	0.08	0.07	.79	0.00
Psychology	1	51.46	44.83	<.001	0.06
Memory	1	68.09	59.32	<.001	0.08
Friends	1	2.58	2.25	.13	0.00
Possessions	1	2.16	1.88	.17	0.00
Body * Psychology	1	0.18	0.16	.69	0.00
Body * Memory	1	0.84	0.73	.39	0.00
Body * Friends	1	1.73	1.51	.22	0.00
Body * Possessions	1	0.58	0.50	.48	0.00
Psychology * Memory	1	3.42	2.98	.09	0.00
Psychology * Friends	1	0.14	0.12	.73	0.00
Psychology * Possessions	1	0.02	0.02	.90	0.00
Memory * Friends	1	0.04	0.04	.85	0.00
Memory * Possessions	1	0.99	0.86	.35	0.00
Friends * Possessions	1	0.20	0.17	.68	0.00
Body * Psychology * Memory	1	1.04	0.90	.34	0.00
Body * Psychology * Friends	1	0.81	0.70	.40	0.00
Body * Psychology * Possessions	1	1.79	1.56	.21	0.00
Body * Memory * Friends	1	0.06	0.05	.82	0.00
Body * Memory * Possessions	1	0.75	0.65	.42	0.00
Body * Friends * Possessions	1	9.97	8.69	.003	0.01
Psychology * Memory * Friends	1	0.56	0.49	0.48	0.00
Psychology * Memory * Possessions	1	1.05	0.92	.34	0.00
Psychology * Friends * Possessions	1	1.75	1.52	.22	0.00
Memory * Friends * Possessions	1	1.13	0.98	.32	0.00
Body * Psychology * Memory * Friends	1	0.79	0.69	.41	0.00
Body * Psychology * Memory * Possessions	1	0.28	0.24	.62	0.00
Body * Psychology * Friends * Possessions	1	0.08	0.07	.79	0.00
Body * Memory * Friends * Possessions	1	0.01	0.01	.93	0.00
Psychology * Memory * Friends * Possessions	1	0.11	0.10	.76	0.00
Body * Psychology * Memory * Friends * Possessions	1	0.11	0.10	.76	0.00
Error	662	1.15			
Total	694				

Table D

ANOVA results for identification with two persons as dependent variable (complete five-factorial design, N=695)

Source	df	Mean Square	F	Sign.	Partial η^2
Intercept	1	5374.60	3145.48	<.001	0.83
Body	1	1.08	0.63	.43	0.00
Psychology	1	0.92	0.54	.46	0.00
Memory	1	0.23	0.13	.71	0.00
Friends	1	0.40	0.23	.63	0.00
Possessions	1	2.97	1.74	.19	0.00
Body * Psychology	1	0.00	0.00	.98	0.00
Body * Memory	1	1.97	1.15	.28	0.00
Body * Friends	1	0.01	0.01	.93	0.00
Body * Possessions	1	0.01	0.00	.95	0.00
Psychology * Memory	1	48.57	28.43	<.001	0.04
Psychology * Friends	1	1.82	1.07	.30	0.00
Psychology * Possessions	1	4.19	2.45	.12	0.00
Memory * Friends	1	11.39	6.67	.01	0.01
Memory * Possessions	1	0.28	0.17	.68	0.00
Friends * Possessions	1	0.68	0.40	.53	0.00
Body * Psychology * Memory	1	0.27	0.16	.69	0.00
Body * Psychology * Friends	1	0.08	0.05	.83	0.00
Body * Psychology * Possessions	1	3.11	1.82	.18	0.00
Body * Memory * Friends	1	2.67	1.56	.21	0.00
Body * Memory * Possessions	1	1.10	0.64	.42	0.00
Body * Friends * Possessions	1	0.22	0.13	.72	0.00
Psychology * Memory * Friends	1	2.42	1.41	.24	0.00
Psychology * Memory * Possessions	1	0.48	0.28	.60	0.00
Psychology * Friends * Possessions	1	0.00	0.00	.97	0.00
Memory * Friends * Possessions	1	0.84	0.49	.49	0.00
Body * Psychology * Memory * Friends	1	0.08	0.05	.83	0.00
Body * Psychology * Memory * Possessions	1	1.82	1.06	.30	0.00
Body * Psychology * Friends * Possessions	1	0.53	0.31	.58	0.00
Body * Memory * Friends * Possessions	1	1.32	0.77	.38	0.00
Psychology * Memory * Friends * Possessions	1	0.33	0.19	.66	0.00
Body * Psychology * Memory * Friends * Possessions	1	6.99	4.09	.04	0.01
Error	663	1.709			
Total	695				

2.1.4. Being two people and split psychology and memory. Figure H illustrates the interaction of the distribution of memory and psychology in predicting the degree of affirming that one is two people after the incident. The means for united conditions (B keeps psychology and memory, or B does not keep memory and does not keep psychology) are lower than for the other two conditions. This disordinal interaction is in line with the suggestion that it is easier to imagine being two people when the two components are divided between the two continuers. While means move across the indifference level, they do not move far away from it.

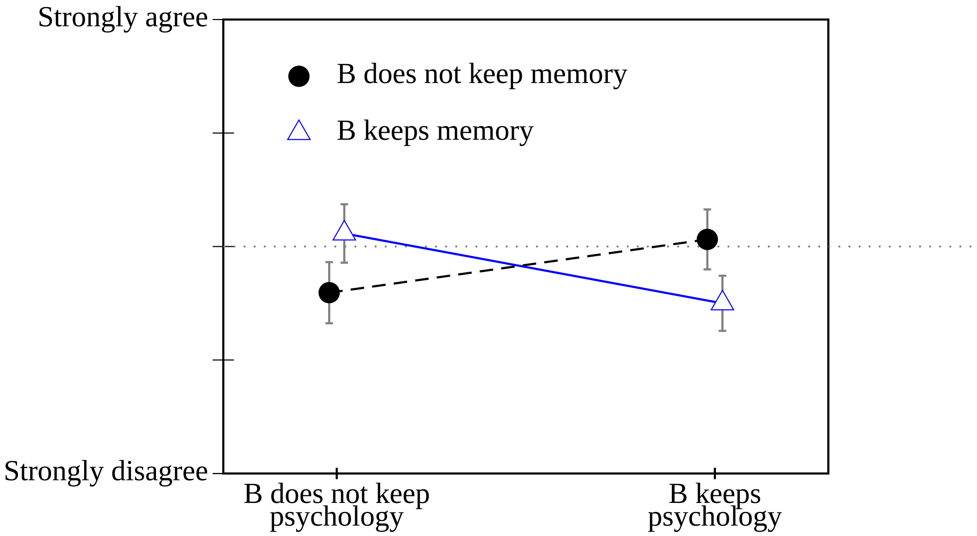

Figure H. Mean response to the statement "‘You are two people after the incident."’ for the four possible distributions of memory and psychology component in Study 1. Whiskers denote the 99% confidence intervals around the mean (N=695).

2.1.5. Identification with continuers and distribution of memory and psychology. To illustrate the relationship between the two identification responses, Figure I presents scatterplots for all combinations of allocating the memory and the psychology dimension that allow to compare both means and distributions of responses to identification ratings with continuers A and B. On the aggregate level, the ANOVA result of two main effects for psychology and memory on identification with the continuers is illustrated in the comparison of means between conditions: For both questions, the mean is higher, when the corresponding person retains either component, and the highest when both components are retained. At the same time, the joint mean distribution illustrates the difference between united and divided faculties. In the two cases, where the psychology and memory are split between the two continuers, (upper right and lower left quadrant) participants on average identify with neither of the continuers. At the same time, when the two components are united (upper left and lower right quadrant), participants on average identify with the continuer possessing both dimensions and do not identify

with the continuer retaining neither of the two components.

The analysis of individual data points offers further insight into the heterogeneity of participants' responses: In all quadrants, a group of participants identifies with both continuers to the same degree. Those participants are split between groups identifying with both to a high, medium, or low degree, which corresponds to the perspectives that one is identical to both continuers at the same time (which violates transitivity), one is neither completely identical nor completely different from both continuers (responses around the middle point) and one is identical with neither of the continuers. In addition, while the means follow the pattern described above, in all four diagrams there are participants in all quadrants. The role of psychology and memory for identification with the continuers is therefore not universally agreed upon, while it is shared by a large cluster of participants.

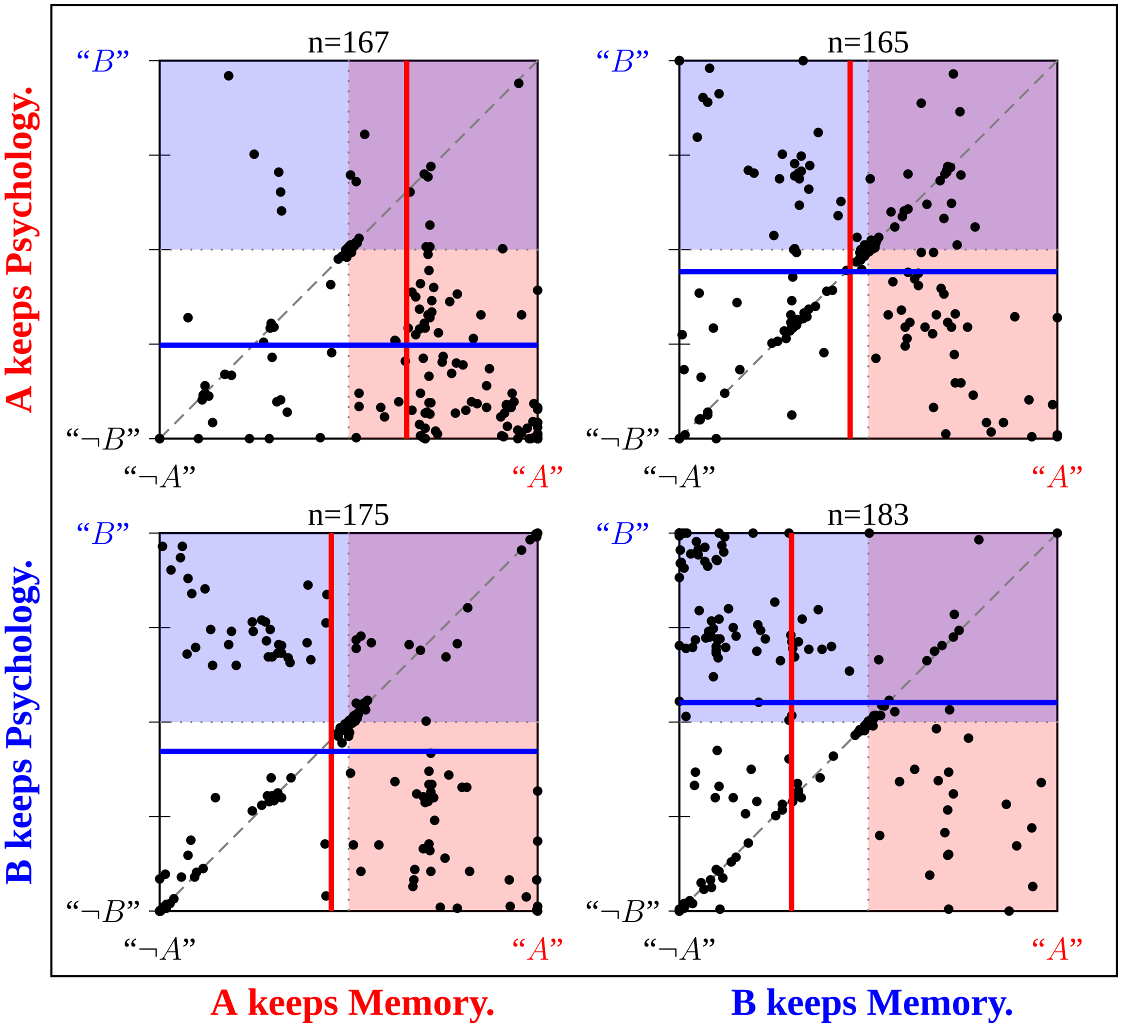

Figure I. Scatterplots of answers to the questions concerning identification with Person A and Person B (after the incident) for the four distributions of memory and personality (Study 1): each scatterplot shows individual data points (black circles), and means for both questions in the subsample (red for identification with A and blue for identification with B). In each diagram the four quadrants are color-coded: A response in the pure red quadrant corresponds with a positive identification with Person A and a negative identification with Person B and a response in the pure blue quadrant corresponds with the opposite identification pattern. Responses in the upper right quadrant signal identification with both continuers, and responses in the white quadrant with identification with none of the continuers. Points on the dashed diagonal correspond to an equal degree of identification with both continuers.

2.1.6. Intercorrelations of postquestionnaire responses. This section presents intercorrelation matrices for the survey questions asked after the scenarios. Table E presents intercorrelation information on the judgments of importance. Participants judged for each of the five component how important they considered it to be for determining identity on the one hand and survival on the other hand. The resulting ten answers are listed with means and standard deviations.

It can be seen that answers concerning the same component are highly but not perfectly correlated. This provides additional support for the positive answer to Research Question 1: Criteria for identity and survival are regarded in a similar light. Yet, not every criterion is treated in the same way: There is a substantial correlation between responses concerning memory and personality, but responses regarding these two variables do not correlate substantially with responses concerning other criteria (the highest correlations are between body and personality responses). On the other hand, there is a stronger relationship between ratings of friends and possessions, and body and possessions. It is noteworthy that there are no negative correlations. In contrast to the allocation task which forced participants to potentially trade off the attributes against each other, there were no such constraints imposed on the answers to these importance questions. The importance placed on the body component seems to be rather orthogonal to the evaluation of psychological criteria, whereas memory and personality tend to be regarded as related.

Table F shows similar information for the six general questions regarding identity and survival after the incident. The acknowledgment of survival is correlated positively with stating that one stays the same person, and negatively with the statement that one does not exist after the incident. Again, these correlations are not perfect, and based on philosophical controversies, a perfect correlation need not be expected. The maximum identification with either continuer is positively correlated with the affirmation of being the same person. Being two people after the incident is considered more or less orthogonal to the question of survival, but responses show some alignment with acknowledgments of non-existence and are negatively correlated with affirmations of staying the same. Further, the correlation with the minimum identification indicates that participants base their feeling of being two people partially on identification with both continuers individually. The mentioned non-correlation with survival indicates that some participants perceive that an extra-ordinary form of existence might not imply the survival of the original. At least in this pattern, the problem of intransitivity of being the same as two distinct continuers is reflected in the answers. Note that the intercorrelations between variable pairs across the two sets are mostly very small (all $|r| < .18$), and for a lack of good theoretical reason these correlations are not reported here.

2.1.7. Weighting of dimensions and monetary allocation: exploratory findings. Comparing the ANOVA results for monetary allocations with the individual judgments of dimensions reveals a slightly puzzling discrepancy. While we observed only two clear main effects in the ANOVA, a substantive number of participants assign medium to high weights to some of the other factors, e.g. regarding body and appearance. This led us to conduct a further step in the analysis that was not planned before this observation. To examine the relationship

Table E

Correlations, means, and standard deviations of the ten judgments of importance for identity and survival: correlations between responses concerning the same component are printed in bold.

Item	Mean	SD	1	2	3	4	5	6	7	8	9
1. Identity: Body and Appearance	4.83	3.04	—								
2. Identity: Personality and Psychology	8.13	2.13	.15***	—							
3. Identity: Memory and Knowledge	8.22	2.11	.07	.47***	—						
4. Identity: Friends	4.63	3.00	.12**	.08*	.13***	—					
5. Identity: Possessions	2.11	2.42	.33***	-.01	-.04	.30***	—				
6. Survival: Body and Appearance	4.52	3.20	**.60*****	.14***	.09*	.06	.26***	—			
7. Survival: Personality and Psychology	7.79	2.48	.09*	**.58*****	.40***	.12**	-.01	.18***	—		
8. Survival: Memory and Knowledge	8.01	2.39	.07	.37***	**.52*****	.16***	-.02	.14***	.59***	—	
9. Survival: Friends	3.69	2.95	.08*	.00	.06	**.61*****	.21***	.12**	.13***	.22***	—
10. Survival: Posessions	2.17	2.60	.21***	-.05	-.06	.17***	**.54*****	.27***	-.01	.06	.39***

$N = 704$, *** p<.001 ** p<.01 * p<.05

Table F

Correlations, means, and standard deviations of the six postquestionnaire items (Study 1)

Item	Mean	SD	1	2	3	4	5
1. You have survived the incident	3.34	1.14	—				
2. You are the same person as before the incident.	2.33	1.10	.44***	—			
3. You are Person A/B after the incident. (Max)	3.41	1.06	.44***	.30***	—		
4. You are Person A/B after the incident. (Min)	2.12	0.87	.04	-.07	.04	—	
5. You do not exist after the incident.	2.58	1.31	-.55***	-.38***	-.40***	.06	—
6. You are two people after the incident.	2.82	1.33	-.05	-.27***	-.07	.32***	.14***

$687 < n < 696$, *** p<.001 ** p<.01 * p<.05

between the judgments of importance and the monetary allocations further, for each of the five dimensions, three groups of participants were formed based on their assigned importance for identity. Those who assigned either very high (8 or higher) or very low importance (2 or lower) were separated from those participants assigning medium importance. For each of the resulting 5×3 subgroups, the difference between the average allocation to the continuer possessing the judged component and the average allocation to the continuer not possessing the component are displayed in Figure J. For all five dimensions, differences were close to 0 for participant groups who judged the component to be of medium importance, indicating that participants with and without the component received comparable allocations. On the other hand, all groups who assigned high importance to a component allocated more money to the continuer with the respective component. Most interestingly, though, participants who assigned low importance actually assigned more money to the participant *without* the component. This was most pronounced for the body component, and less pronounced for friends and possessions. The values for psychology and memory were based on very small samples, as few participants considered these two factors unimportant.

A negative weighting of dimensions is difficult to align with the closest continuer theory. It would imply that the continuity of an attribute would be considered to cause a decrease in closeness. One possible explanation would be a discrepancy with the perceived state of the self, and the “true” state the self should be in. Approaches studying the “true self” find asymmetries in participants’ perspectives on their and other participants’ interpretation of actions: people are assumed to be fundamentally moral even if their actions seem to disconfirm this notion (Newman et al., 2015). Actions consistent with the assumption are instead assumed to be an expression of this moral true self. Likewise, positive changes to the self are assumed to be less disruptive to the self than negative changes (Molouki & Bartels, 2017) or even experienced as self-discoveries (Bench, Schlegel, Davis, & Vess, 2015), with the future holding more potential for positive than for negative events (Newby-Clark & Ross, 2003). In our scenario, components were not destroyed or damaged, but replaced by a randomly selected variant out of the population. Now, given the impression that a specific dimension is of lower than average quality (judged by the participant), a replacement could lead to a perceived improvement (see also 2.2.6). This change could, given the findings above, even be associated with coming closer

to what the self *should* be. Given the lack of further information or questions, this explanation is purely speculative, but it should demonstrate how the present scenario might be utilized productively in research aiming to uncover impressions of the "true self".

This finding would not have been observable with a single allocation task or only attributions of importance. While the analysis was unplanned and the results should be considered exploratory, we find based on this additional analysis limited support for Hypothesis 1a (in addition to further support for Hypothesis 1b and Hypothesis 1c). The support is limited as only the answers of a (nonetheless non-negligible) subset of participants allocate more money to the continuer inheriting body and appearance. The discovered relationship is further explored (and validated) with censored regression modeling.

2.1.8. Body Importance and allocations: Exploratory regression models. The analysis of judgments of importance (reported in the results section of the main text) revealed the possibility that respondents indicating very low values on the five dimensions might actually value these dimensions negatively in the sense of paying systematically less to the continuer in possession of the evaluated component. This seemed especially relevant for judgments regarding the body dimension. To further explore this possibility, we estimated a sequence of regression models, incorporating some of the evaluations. As this modeling was conducted in light of the previous data analysis, we consider these models to be exploratory.

As a baseline model (Model I), we first estimated a regression model with the location of the five components as predictors and the allocation as criterion. This model is essentially similar to an ANOVA with the five main effects for location. We focused the models on the allocation to person B as criterion. The five locations were coded as 1 for "present in B" and -1 for "present in A". Regression equation 1 summarizes this model:

$$\begin{aligned} \text{Allocation\_to\_B}_i = \alpha &+ \beta_1 \text{Body\_B}_i \\ &+ \beta_2 \text{Psychology\_B}_i \\ &+ \beta_3 \text{Memory\_B}_i \\ &+ \beta_4 \text{Friends\_B}_i \\ &+ \beta_5 \text{Possessions\_B}_i + \epsilon \end{aligned} \tag{1}$$

In the equation, Body_B_i takes on the value 1, if B inherits the body and -1 if A inherits the body. The parameter estimates can be interpreted as the change in allocation due to retaining or losing the component (in this sense, as the value of the dimension for allocation purposes). The difference between continuers who retain and lack the dimension, respectively, would be twice this amount. The constant is expected to be estimated at around \$50,000, which would represent symmetry in allocations to A and B. As we intend to introduce some continuous variables, we consider the censored nature of the criterion. Allocations cannot be lower than \$0 and cannot be higher than \$100,000. For this reason we use a Tobit regression approach for all models. Calculations were implemented in R (version 3.2.5, R Core Team, 2015), using the "censReg"-package (Henningsen, 2013).

The results for all models are listed in Table G. Corresponding to the two significant main

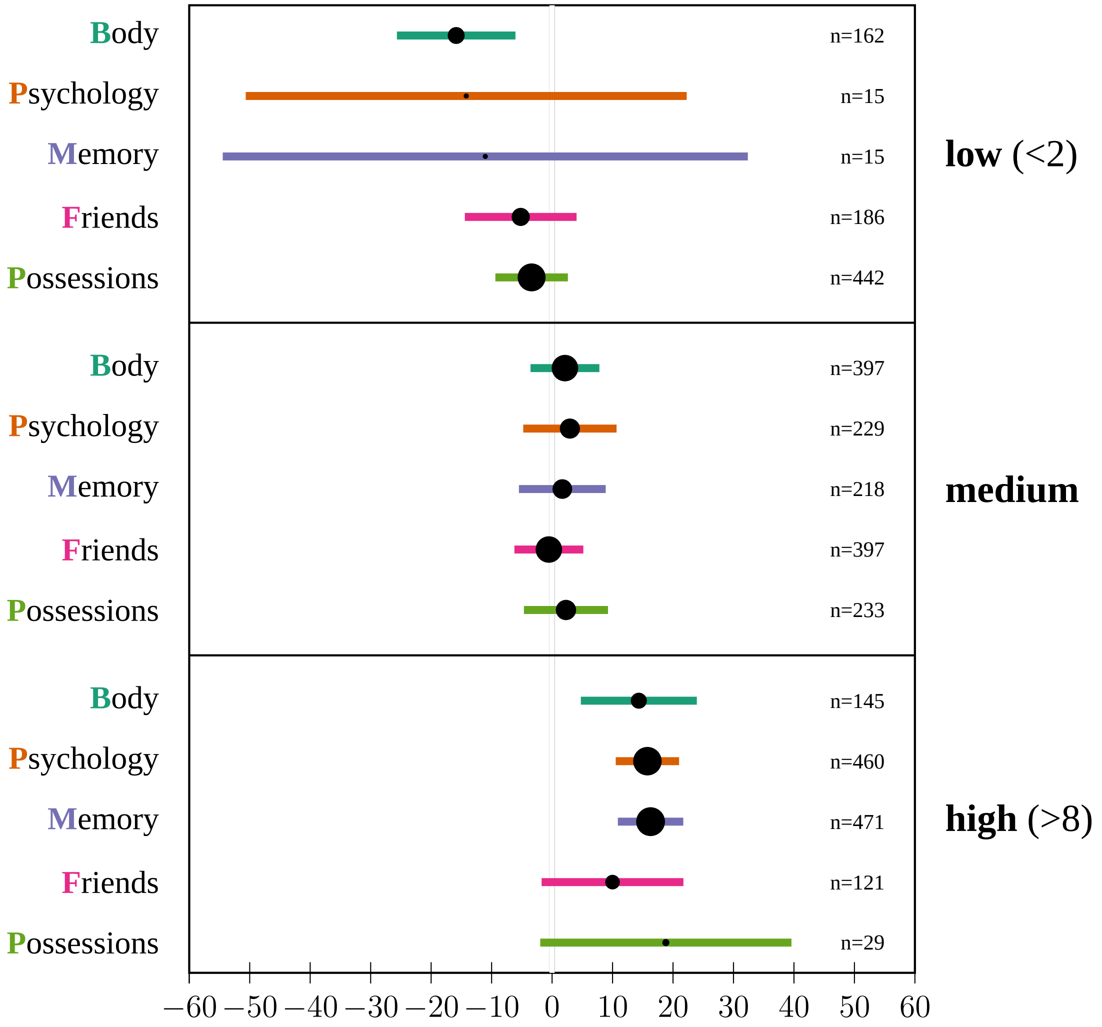

Figure J. Mean difference in allocation (in thousands of dollar) between continuer who inherits attribute and continuer who does not inherits the row attribute separated by assigned importance to attribute (Study 1): The width of the colored bars is the 99% confidence interval around the mean. The area of the markers is proportional to the subsample size for this row. Each participant contributes values to exactly five rows based on the participant's ratings (sample sizes for each attribute across the three rating categories add up to $N = 704$). Note that each participant contributes to exactly five rows that depend on the participant's rating of the five attributes.

effects in the original ANOVA results parameters β_2 and β_3 are significantly different from 0. The constant is estimated close to $50,000. The values for these two components are estimated as close to $6,000 each.

To mode the effect of low judgments of importance, we created a derived variable from the original importance judgment (bounded between 0 and 10). The transformation takes the form shown in Equation 2

$$x' = 0 \begin{cases} 0, x \geq 5 \\ x - 5, x < 5, \end{cases} \tag{2}$$

or equivalently $x' = min(x - 5, 0)$. This transformed evaluation is multiplied by the dummy variable for the component to form the variable used in Equation 3:

$$\begin{aligned} \text{Allocation\_to\_B}_i = \alpha &+ \beta_1 \text{Body\_B}_i \\ &+ \beta_2 \text{Psychology\_B}_i \\ &+ \beta_3 \text{Memory\_B}_i \\ &+ \beta_4 \text{Friends\_B}_i \\ &+ \beta_5 \text{Possessions\_B}_i \\ &+ \beta_6 \text{Body\_B}_i \cdot min(\text{BodyImportance}_i - 5, 0) + \epsilon \end{aligned} \tag{3}$$

The parameter β_6 can be interpreted as the reduction in value for the body component for each scale point the importance of the body component is judged below 5. For participants with judgments above 5, the value is solely determined by β_1 as before. The results of Model II are shown in the second column of Table G: While the coefficients for memory and psychology stay at about the same level, the coefficient for the new variable is estimated as significantly different from 0. In addition, the parameter coding for the value of the body component is now estimated as significantly different from 0, as well. The estimated value is lower than for the two other components, though. This finding leads to the interpretation that the body component is evaluated positively for participants with positive judgments of relevance, but is valued negatively for the group with low judgments of relevance. This corresponds to the finding for the extreme lower group. The difference regarding the value of the body component is visualized in Figure K.

In Model I, the dimension's value is estimated to be rather low. Model II allows for value differentiation: The dimension's value is estimated higher for the group affirming its importance and negative for participants rejecting its importance. The extreme negative value is even higher than the positive value for memory and personality. Note again, that the difference between the two continuers possessing and lacking the original component would be double the parameter estimate.

Finally, to check for the robustness of this finding, we estimated Model III, in which we add technical variables for the other four components, leading to Model III shown in Equation 4:

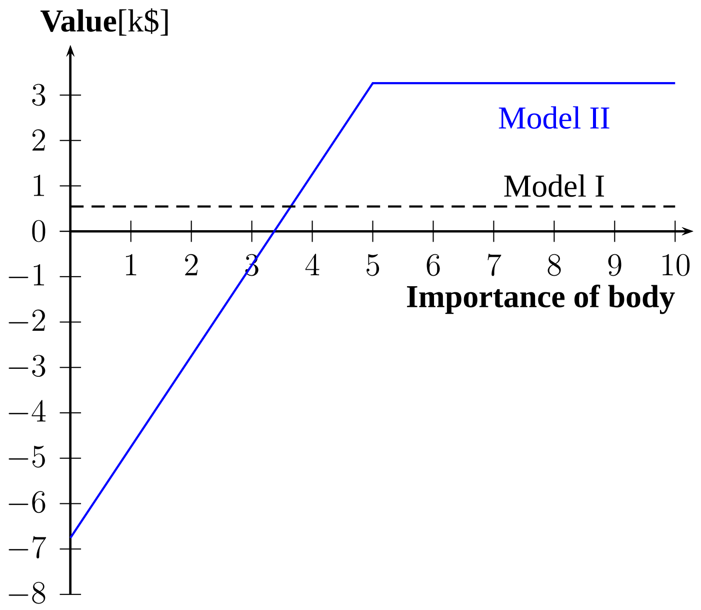

Figure K. Estimated value of body continuity in Model I and Model II depending on the judgment of body importance (Study 1)

$$
\begin{aligned}
\text{Allocation\_to\_B}_i = \alpha &+ \beta_1 \text{Body\_B}_i \\
&+ \beta_2 \text{Psychology\_B}_i \\
&+ \beta_3 \text{Memory\_B}_i \\
&+ \beta_4 \text{Friends\_B}_i \\
&+ \beta_5 \text{Possessions\_B}_i \\
&+ \beta_6 \text{Body\_B}_i \cdot min(\text{BodyImportance}_i - 5, 0) \\
&+ \beta_7 \text{Psychology\_B}_i \cdot min(\text{PsychologyImportance}_i - 5, 0) \\
&+ \beta_8 \text{Memory\_B}_i \cdot min(\text{MemoryImportance}_i - 5, 0) \\
&+ \beta_9 \text{Friends\_B}_i \cdot min(\text{FriendsImportance}_i - 5, 0) \\
&+ \beta_{10} \text{Possessions\_B}_i \cdot min(\text{PossessionsImportance}_i - 5, 0) + \epsilon
\end{aligned} \tag{4}
$$

The results for this model are presented in the third column of Table G. The direction and size of the previously observed effects does not substantially change. One new parameter that is significantly different from 0 is the parameter coding for participants attesting a very low importance to memory. The model error is not reduced substantially from Model I to Model III, while the number of parameters increases.

One should add the caveat that the chosen functional form might not be the most appropriate for capturing the exact relationship between the variables. Nonetheless, this analysis

Table G

Results for the Tobit regressions (Study 1): Parameter estimates are shown in the top rows, t-values in parantheses below.

Variable	Parameter	Model I	Model II	Model III
constant	α	49266.91^{***}	49197.28^{***}	49198.16^{***}
		(55.33)	(55.82)	-56.19
body in B	β_1	545.93	3262.85^{**}	2749.94^{*}
		(0.61)	(2.89)	(2.43)
psychology in B	β_2	5936.87^{***}	5263.94^{***}	5626.62^{***}
		(6.68)	(5.88)	(6.07)
memory in B	β_3	6127.48^{***}	5789.22^{***}	5941.25^{***}
		(6.89)	(6.55)	(6.48)
friends in B	β_4	572.53	502.84	1397.48
		(0.64)	(0.57)	(1.23)
possessions in B	β_5	-127.07	-134.65	1383.55
		(-0.14)	(-0.15)	(0.76)
negative weight body	β_6		2003.72^{***}	1694.63^{**}
			(3.86)	(3.21)
negative weight personality	β_7			2324.90
				(1.90)
negative weight memory	β_8			2933.01^{*}
				(2.31)
negative weight friends	β_9			760.34
				(1.54)
negative weight possessions	β_{10}			458.72
				(0.91)
$log(\sigma)$		10.06^{***}	10.05^{***}	10.04^{***}
		(350.24)	(349.95)	(349.72)

*** p<001 ** p<01 * p<05

consolidates the descriptive finding, that some groups of participants might put a negative value on components. This possibility could be explored both for the body component and for the memory component. An ad-hoc interpretation could illustrate these relationships with cases of traumatic or unwanted memories, or physical bodies that do not conform to a personal body ideal. Confirmation would need a replication of this finding in a different sample and more within-subject variation to identify the form of the relationship and additional data to find systematic explanations for this finding.

In summary, a closer analysis of individual answers reveals that participants showed a high degree of variance especially regarding the importance of the location of the body. While some participants affirmed the body as essential for their identity judgments, others assigned a very low importance and were even shown to give less money to the continuer inheriting the

body component. This difference is consistent with the idea that participants who assigned a low degree of importance to the body component valued their own body negatively. From this point of view, a replacement by a randomly chosen other body might seem like an improvement.

2.1.9. Cluster analyses of postquestionnaire items: Explicit weighting of dimensions. Participants evaluated the five dimensions (body, personality, memory, friends, and possessions) both for identity and survival. The analyses above focused on univariate and bivariate statistics. To study typical patterns of responses across all ten questions, we conducted a k-means cluster analysis with 6 cluster centers in SPSS 15. The cluster center means after the final iteration are presented in Figure L. The cluster solution illustrates commonalities and differences between participant groups: For all clusters, memory and psychology were considered to be the most important, both for identity and survival. For all groups (with one minor exception), possessions were considered not important. Also, responses regarding identity and survival are close t each other, again confirming the answer to Research Question 1. The cluster solution also demonstrates heterogeneity in responses. In particular the six clusters can be characterized as follows:

1. For participants in cluster A ($n_A = 108$, 15.3%), all attributes are important to some relevant degree, with psychology and memory in the first group, followed by body, and then by both friends and possessions (which both received average responses above 5).
2. Participants in cluster B ($n_B = 178$, 25.3%) answer in accordance with the ANOVA results: They place a high importance on psychology and memory and low importance on all other criteria.
3. Participants in cluster C ($n_C = 122$, 17.3%) are similar to participants in cluster B in that the highest values are chosen for psychology and memory. In contrast participants in this cluster indicate relatively higher values for friends.
4. Participants in cluster D ($n_D = 91$, 12.9%) do not seem to regard any of the criteria as particular important for identity and survival. Chosen weights are much lower than those chosen by participants in cluster A. The highest value are barely above the medium point of the scale and those are for psychology and memory, but only when answering about their importance for identity.
5. Participants in cluster E ($n_E = 95$, 13.5%), assign the highest importance to psychology and memory, followed by body, and values for friends still higher than 5.
6. Participants in cluster F ($n_F = 110$, 15.6%) also assign high values to psychology and memory, followed by those for body, but in contrast to participants in cluster E they place a low value on friends.

In summary, only a minority of respondents value possessions at all and all clusters assign the highest importance to psychology and memory. Some add body to the list of important criteria, some friends, and some both body and friends. This is consistent with, but adds some context to the reported ANOVA results.

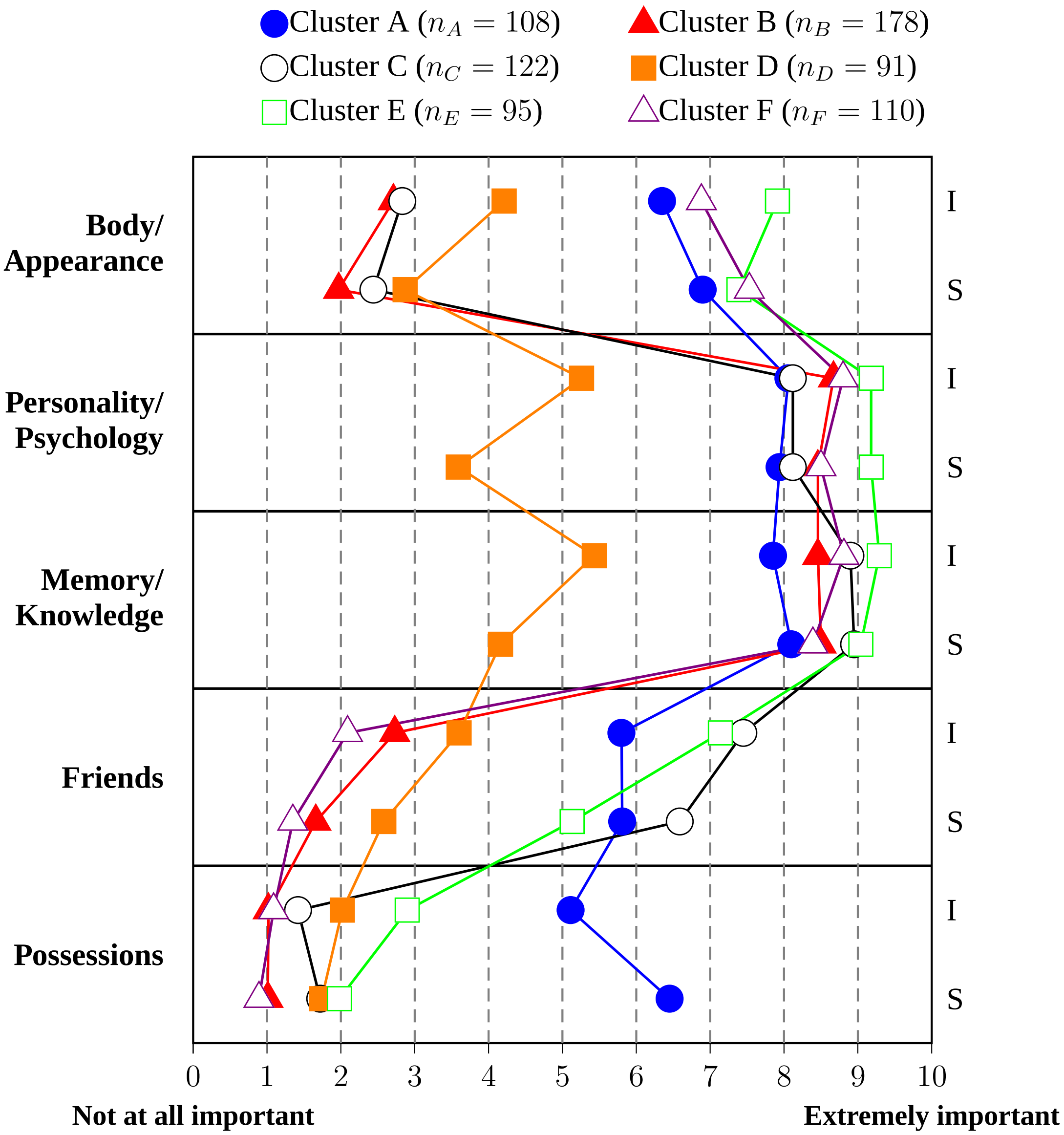

Figure L. Cluster centers after final iteration for a cluster analysis of the ten attribute evaluations for identity (I) and survival (S) in Study 1

2.1.10. Cluster analyses of postquestionnaire items: Re-identification after fission. Participants gave six answers to the postquestionnaire regarding the state after the incident. These answers were reported separately in the manuscript, in addition to bivariate correlations and some ANOVA results that demonstrated their dependency on features of the scenario. Here, we focus on the multivariate pattern of responses. More than for the separate evaluations of dimensions, it is relevant to consider here that these responses were given to systematically varied scenarios. The response pattern is therefore both determined by inter-individual differences in general judgment and reactions to specific scenarios.

We conducted a k-means cluster analysis on the six responses to the post-questionnaire (as the composition of Person A and Person B depends on the scenario, we used the minimum and maximum response as input to the cluster analysis). We determined the number of initial cluster centers as six, the analysis was conducted as cluster center anaylsis in SPSS 15. Cluster center means in the final iteration are presented in Figure M. Compared to the previous cluster analysis, the response patterns in clusters are more divergent, but each cluster's mean response pattern can be linked to a certain interpretation of the situation. One general observation might be that the degree of affirmation of survival is inversely linked to the degree of affirmation of non-existence after the incident. The answer patterns in the six clusters can be described in the following way:

1. Participants in cluster A' ($n_{A'} = 135$, 19.7%) believe that they have survived the incident, but that they are not the same, afterwards. They affirm their existence but also believe that they are two people afterwards. Their identification with the two continuers is medium or high.

2. Participants in cluster B' ($n_{B'} = 72$, 10.5%) do not believe that they have survived the incident and do not believe that they are the same after it. They believe that they do not exist after the incident, but do not believe that they are two people after the incident. Their identification with either continuer is low, after the incident.

3. Participants in cluster C' ($n_{C'} = 140$, 20.5%) believe that they are not the same after the incident and are skeptical about their survival. They simultaneously affirm that they are two people after the incident and that they do not exist after it. Their identification with either continuer is medium.

4. Participants in cluster D' ($n_{D'} = 154$, 22.5%) believe that they have survived the incident, but that they are not the same, afterwards. They are not convinced that they do not exist after it, nor that they are two people. Their identification is relatively high with one continuer, but still medium to low with the other.

5. Participants in cluster E' ($n_{E'} = 51$, 7.5%) believe that they have survived the incident as the same person as before. They believe that they exist, afterwards, but that they are two people. They indicate a medium identification with both continuers.

6. Participants in cluster F’ ($n_{F'} = 132$, 19.3%) also believe that they have survived the incident as the same person. They believe that they exist, afterwards, but they do not believe that they are two people. Their identification with one continuer is very high and their identification with the other continuer is very low (they show the largest difference in identification).

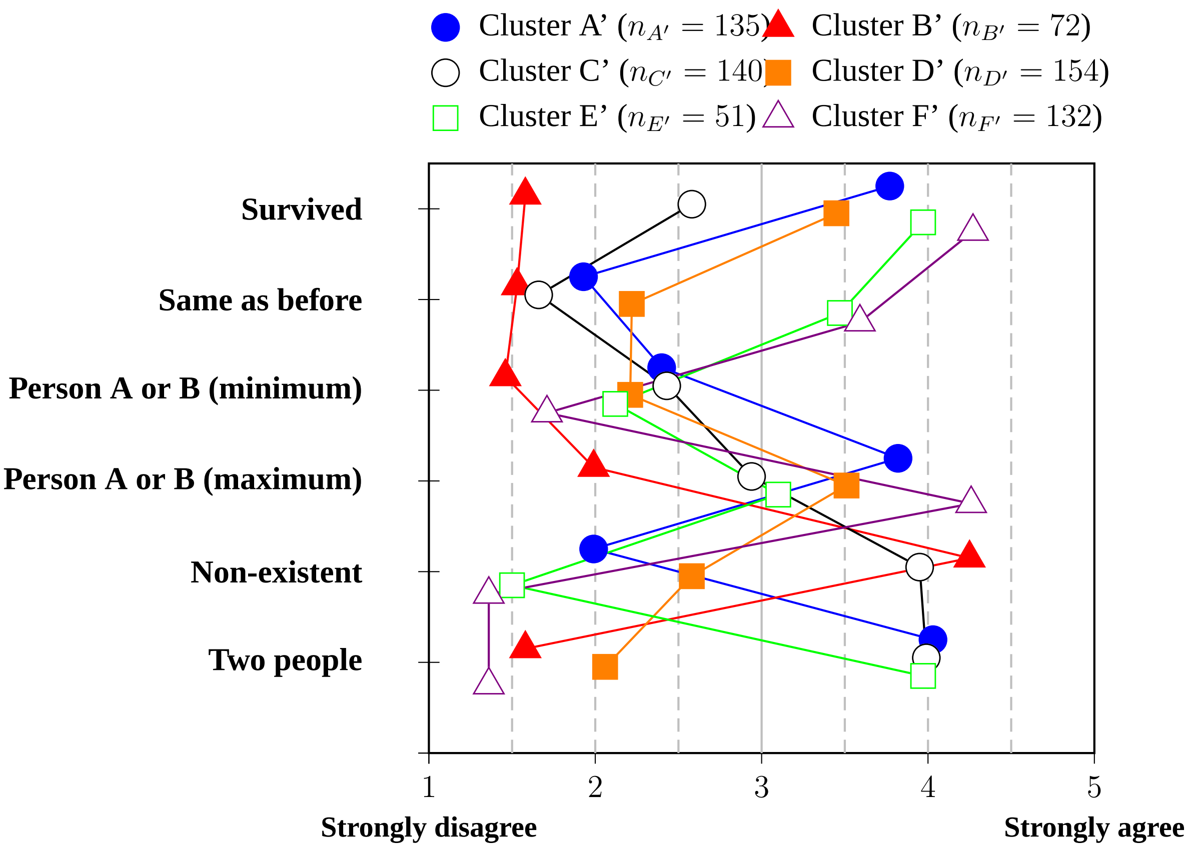

Figure M. Cluster centers after final iteration for a cluster analysis of the six postquestionnaire items in Study 1

This pattern of results allows to qualify and contextualize some of the findings indicating intransitivity of judgments. Only participants in cluster F’ see a clear solution, which is facilitated by a large perceived difference between the two persons that are candidates for self-identification. Therefore they can make an unambiguous decision and do not see a high degree of personal change, affirm their existence and see themselves as one person after the incident

(one option is good enough, the other clearly is not). This corresponds to Case 4 in the decision scheme of Nozick (1981). In contrast, participants in cluster B' do not identify strongly enough with either continuer, therefore they indicate that they do not exist after the incident (none of the options is good enough). There is an ambiguity in this response, though. If participants see a high and indistinguishable degree of closeness with both continuers, they might also describe it as being neither person. Reactions could therefore correspond either with Case 2or Case 3 in the decision scheme.

Participants in the other four clusters face a dilemma in deciding between the two participants that is resolved in different ways. Participants in cluster A' are faced with two continuers they both would consider appropriate to identify with (both options are good enough). They opt for the solution to see themselves as existing as two people afterwards. Participants in cluster C' do not see a large difference between the two continuers, and consequently affirm that they did not survive, do not exist and that they exist as two people after the incident. Participants in cluster D' see themselves as somewhat changed after the incident, as they are able to identify with one of the continuers, but still see a closer connection to the other than participants in cluster B'. These participants are undecided about the other categories of judgment. Participants in cluster E' solve the dilemma —being faced with two continuers that are appropriate enough—by affirming their existence as two people.

By interpreting the seeming violations of transitivity as part of this sensemaking process, it becomes possible to align some of these reactions with philosophical interpretations of identity in the fission case. The idea of existing as two people is affirmed by three of the clusters and rejected by the other three clusters.

Cluster B' resolves the dilemma of two possible continuers possibly in a similar way to Parfit (1984): as the original cannot be identical to two people with close to equivalent claims, the original cannot exist after the incident (as stated above, this might also be a reaction to two continuers not being close enough). On the other hand, they link this assessment to the question of survival, as well, where many philosophers would come to a different result. Cluster F' faces no dilemma, as one continuer is chosen (Case 4). Cluster D' acknowledges change but is still able to choose a continuer between the two options. This is a more informative variant of Case 4 in the decision scheme, as participants in this situation both experience the dilemma of finding two changed continuers (this dilemma is not experienced in cluster F'), and resolve it in the way the decision scheme predicts.

Cluster A', C', and E' all affirm to be two people after the incident, a reaction that implies an intransitive identity relation. Cluster C' simultaneously acknowledges non-existence and change, which again might make this pattern of answers compatible with a Parfitian interpretation of identity ("'being two people"' is not equated with existing in this group). Clusters A' and E' differ in the degree to which they see themselves as changed, but both groups of participants strongly affirm that they exist as two people after the incident. This is incompatible with both a Parfitian and a closest-continuer interpretation of personal identity, but it could be—in particular for cluster E'—charitably linked to a conception of persons as branching with multiple futures (e.g., Moyer, 2008), see the discussion in the main manuscript.

Table H

Differences between clusters in average monetary allocation and scenario conditions in Study 1

	larger money share	larger attribute share	proportion memory/ psychology united
Cluster A’	65.6%	67.6%	45.2%
Cluster B’	64.6%	69.7%	52.8%
Cluster C’	61.0%	65.9%	36.4%
Cluster D’	66.0%	67.7%	55.2%
Cluster E’	60.3%	69.0%	45.1%
Cluster F’	72.9%	71.1%	67.4%

Table H offers some supporting results for these interpretations. Clusters differ significantly in three variables (no other variable was tested): maximum share in monetary allocation ($F(5, 678) = 8.405$, $p < .001$)), maximum share in attributes ($\chi^2(10) = 22.3$, $p = .01$, this is a 6×3 two-sided independence test, as only three maximum percentages can exist: 60%, 80%, 100%), and proportion of scenarios, in which memory and psychology are united in one continuer ($\chi^2(5) = 29.8$, $p < .001$, two-sided independence test). Note that none of these variables was part of the clustering procedure (we omit tests for differences regarding the cluster-defining variables). Moreover, the pattern of differences aligns with the interpretation of decisional conflict: Clusters A’, C’, and E’ who affirm to be two people after the incident have the lowest share of scenarios with united memory and psychology. Cluster F’ is characterized by the largest proportion of united psychology and memory, the largest share of attributes and the largest proportion of money assigned to one survivor. Again, the relative position of memory and psychology seems to be more important than the number of attributes retained. Nonetheless, the analysis also demonstrates that there are interindividual differences in interpretation that cannot be reduced to differences in the encountered scenario.

These results suggest that participants do not share a unified conception of personal identity. Many but not all responses confirm with a closest-continuer interpretation. Nonetheless, care should be taken not to automatically judge the majority of lay-people as simply mistaken based on the evidence of a lack of transitivity in their identity judgments. Future research should explore the concept of persons and develop a more fine-grained picture of what participants mean, when they endorse the existence as two persons.

2.2. Study 2

2.2.1. Full ANOVA Tables. The full results for the ANOVAs referenced in the manuscript are presented in Table I for the inheritance decision weighted by decision difficulty, in Table J for decision difficulty.

The results for identification with either continuer are shown in Table K for Person A and in Table L for Person B. Finally, the results for an ANOVA with the endorsement for the statement “You are two people after the incident” as criterion is given in Table M and the

Table I

ANOVA results for decision difficulty weighted by decision direction (-10, decision for A with minimum difficulty, to +10, decision for B with minimum difficulty) in Study 2 (complete five-factorial design, N=821)

Source	df	Mean Square	F	Sign.	Partial η^2
Intercept	1	3.00	.122	.73	0.00
Body	1	295.31	11.99	<.001	0.02
Psychology	1	2391.84	97.07	<.001	0.11
Memory	1	7278.48	295.38	<.001	0.27
Friends	1	187.95	7.63	.006	0.01
Possessions	1	39.85	1.62	.20	0.00
Body * Psychology	1	29.06	1.18	.28	0.00
Body * Memory	1	1.45	0.06	.81	0.00
Body * Friends	1	24.16	.98	.32	0.00
Body * Possessions	1	0.19	0.01	.93	0.00
Psychology * Memory	1	3.20	0.13	.72	0.00
Psychology * Friends	1	43.02	1.75	.19	0.00
Psychology * Possessions	1	0.34	0.01	.91	0.00
Memory * Friends	1	9.27	0.38	.54	0.00
Memory * Possessions	1	3.85	0.16	.69	0.00
Friends * Possessions	1	0.41	0.02	.90	0.00
Body * Psychology * Memory	1	4.80	0.20	.66	0.00
Body * Psychology * Friends	1	81.67	3.31	.07	0.00
Body * Psychology * Possessions	1	11.39	0.46	.50	0.00
Body * Memory * Friends	1	3.64	0.15	.70	0.00
Body * Memory * Possessions	1	1.53	.06	.80	0.00
Body * Friends * Possessions	1	2.00	0.08	.78	0.00
Psychology * Memory * Friends	1	13.43	0.55	.46	0.00
Psychology * Memory * Possessions	1	33.91	1.38	.24	0.00
Psychology * Friends * Possessions	1	41.05	1.67	.20	0.00
Memory * Friends * Possessions	1	5.32	0.22	.64	0.00
Body * Psychology * Memory * Friends	1	3.73	0.15	.70	0.00
Body * Psychology * Memory * Possessions	1	2.48	0.10	.75	0.00
Body * Psychology * Friends * Possessions	1	5.22	0.21	.65	0.00
Body * Memory * Friends * Possessions	1	0.07	0.003	.96	0.00
Psychology * Memory * Friends * Possessions	1	0.55	0.02	.88	0.00
Body * Psychology * Memory * Friends * Possessions	1	63.02	2.56	.11	0.00
Error	789	24.64			
Total	821				

ANOVA for “You do not exist after the incident.” in Table N.

Table J

ANOVA results for decision difficulty in Study 2 (complete five-factorial design, N=821)

Source	df	Mean Square	F	Sign.	Partial η^2
Intercept	1	17500.12	2930.36	<.001	0.75
Body	1	7.33	1.00	.32	0.00
Psychology	1	6.33	0.87	.35	0.00
Memory	1	0.00	0.00	.99	0.00
Friends	1	0.55	0.07	.79	0.00
Possessions	1	36.45	4.98	.03	0.01
Body * Psychology	1	9.75	1.33	.25	0.00
Body * Memory	1	50.50	6.90	.009	0.01
Body * Friends	1	13.33	1.82	.18	0.00
Body * Possessions	1	3.60	0.49	.48	0.00
Psychology * Memory	1	412.27	56.31	<.001	0.07
Psychology * Friends	1	42.76	5.84	.02	0.01
Psychology * Possessions	1	3.76	0.51	.47	0.00
Memory * Friends	1	39.07	5.34	.02	0.01
Memory * Possessions	1	13.52	1.85	.17	0.00
Friends * Possessions	1	0.36	0.05	.83	0.00
Body * Psychology * Memory	1	15.68	2.14	.14	0.00
Body * Psychology * Friends	1	34.43	4.70	.03	0.01
Body * Psychology * Possessions	1	3.74	0.51	.48	0.00
Body * Memory * Friends	1	20.91	2.865	.09	0.00
Body * Memory * Possessions	1	1.50	0.20	.65	0.00
Body * Friends * Possessions	1	7.05	0.96	.33	0.00
Psychology * Memory * Friends	1	10.20	1.39	.24	0.00
Psychology * Memory * Possessions	1	3.16	0.43	.51	0.00
Psychology * Friends * Possessions	1	0.01	0.00	.98	0.00
Memory * Friends * Possessions	1	0.73	0.10	.75	0.00
Body * Psychology * Memory * Friends	1	6.72	0.92	.34	0.00
Body * Psychology * Memory * Possessions	1	0.97	0.13	.72	0.00
Body * Psychology * Friends * Possessions	1	24.44	3.34	.07	0.00
Body * Memory * Friends * Possessions	1	0.44	0.06	.81	0.00
Psychology * Memory * Friends * Possessions	1	0.15	0.02	.89	0.00
Body * Psychology * Memory * Friends * Possessions	1	17.22	2.35	.13	0.00
Error	789	5776.36			
Total	821				

Table K

ANOVA results for identification with Person A in Study 2 (QII5) as dependent variable (complete five-factorial design, N=821)

Source	df	Mean Square	F	Sign.	Partial η^2
Intercept	1	4536.09	2114.96	<.001	0.73
Body	1	12.36	5.76	.03	0.01
Psychology	1	169.21	78.89	<.001	0.09
Memory	1	285.25	133.13	<.001	0.14
Friends	1	8.73	4.07	.04	0.01
Possessions	1	0.46	0.21	.65	0.00
Body * Psychology	1	0.00	0.00	>.99	0.00
Body * Memory	1	0.92	0.43	.51	0.00
Body * Friends	1	2.09	0.98	.32	0.00
Body * Possessions	1	0.08	0.04	.85	0.00
Psychology * Memory	1	4.84	2.26	.13	0.00
Psychology * Friends	1	12.93	6.03	.01	0.01
Psychology * Possessions	1	0.63	0.29	.59	0.00
Memory * Friends	1	2.46	1.15	.28	0.00
Memory * Possessions	1	0.05	0.02	.88	0.00
Friends * Possessions	1	0.00	0.00	.99	0.00
Body * Psychology * Memory	1	0.97	0.45	.50	0.00
Body * Psychology * Friends	1	0.31	0.14	.71	0.00
Body * Psychology * Possessions	1	0.15	0.07	.79	0.00
Body * Memory * Friends	1	2.78	1.30	.256	0.00
Body * Memory * Possessions	1	0.78	0.37	.55	0.00
Body * Friends * Possessions	1	0.17	0.08	.78	0.00
Psychology * Memory * Friends	1	0.72	0.33	.56	0.00
Psychology * Memory * Possessions	1	0.03	0.01	.91	0.00
Psychology * Friends * Possessions	1	0.73	0.34	.56	0.00
Memory * Friends * Possessions	1	1.65	0.77	.38	0.00
Body * Psychology * Memory * Friends	1	0.98	0.46	.50	0.00
Body * Psychology * Memory * Possessions	1	0.00	0.00	.98	0.00
Body * Psychology * Friends * Possessions	1	0.02	0.09	.77	0.00
Body * Memory * Friends * Possessions	1	0.05	0.02	.88	0.00
Psychology * Memory * Friends * Possessions	1	0.06	0.03	.86	0.00
Body * Psychology * Memory * Friends * Possessions	1	0.00	0.00	.98	0.00
Error	789	2.15			
Total	821				

Table L

ANOVA results for identification with Person B in Study 2 (QII6) as dependent variable (complete five-factorial design, N=821)

Source	df	Mean Square	F	Sign.	Partial η^2
Intercept	1	4448.05	2123.02	<.001	0.73
Body	1	7.41	3.54	.06	0.00
Psychology	1	146.27	69.81	<.001	0.08
Memory	1	263.08	125.57	<.001	0.14
Friends	1	8.86	4.23	.04	0.01
Possessions	1	0.50	0.14	.63	0.00
Body * Psychology	1	3.95	1.88	.17	0.00
Body * Memory	1	24.84	11.86	.001	0.02
Body * Friends	1	0.47	0.22	.06	0.00
Body * Possessions	1	0.13	0.06	.81	0.00
Psychology * Memory	1	0.39	0.18	.67	0.00
Psychology * Friends	1	1.57	0.75	.39	0.00
Psychology * Possessions	1	1.15	0.55	.46	0.00
Memory * Friends	1	0.05	0.02	.88	0.00
Memory * Possessions	1	2.52	1.21	.27	0.00
Friends * Possessions	1	3.61	1.72	.19	0.00
Body * Psychology * Memory	1	2.40	1.15	.28	0.00
Body * Psychology * Friends	1	14.14	6.75	.01	0.01
Body * Psychology * Possessions	1	0.76	0.36	.55	0.00
Body * Memory * Friends	1	0.15	0.07	.79	0.00
Body * Memory * Possessions	1	3.38	1.61	.20	0.00
Body * Friends * Possessions	1	0.24	0.11	.74	0.00
Psychology * Memory * Friends	1	2.52	1.20	.27	0.00
Psychology * Memory * Possessions	1	1.66	0.79	.37	0.00
Psychology * Friends * Possessions	1	1.10	0.52	.47	0.00
Memory * Friends * Possessions	1	0.92	0.44	.51	0.00
Body * Psychology * Memory * Friends	1	0.01	0.00	.96	0.00
Body * Psychology * Memory * Possessions	1	0.70	0.34	.56	0.00
Body * Psychology * Friends * Possessions	1	0.99	0.47	.49	0.00
Body * Memory * Friends * Possessions	1	0.25	0.12	.73	0.00
Psychology * Memory * Friends * Possessions	1	0.00	0.00	.99	0.00
Body * Psychology * Memory * Friends * Possessions	1	1.40	0.67	.41	0.00
Error	789	2.10			
Total	821				

Table M

ANOVA results for agreement with statement "You are two people after the incident" in Study 2 (QII8) as dependent variable (complete five-factorial design, N=821)

Source	df	Mean Square	F	Sign.	Partial η^2
Intercept	1	3923.69	1579.59	<.001	0.67
Body	1	0.02	0.01	.94	0.00
Psychology	1	4.62	1.86	.17	0.00
Memory	1	0.84	0.34	.56	0.00
Friends	1	0.36	0.14	.70	0.00
Possessions	1	0.32	0.13	.72	0.00
Body * Psychology	1	0.06	0.03	.87	0.00
Body * Memory	1	24.84	10.00	.002	0.01
Body * Friends	1	0.00	0.00	.99	0.00
Body * Possessions	1	0.81	0.33	.57	0.00
Psychology * Memory	1	26.04	10.48	.001	0.01
Psychology * Friends	1	13.35	5.38	.02	0.01
Psychology * Possessions	1	0.05	0.02	.88	0.00
Memory * Friends	1	38.74	15.60	<.001	0.02
Memory * Possessions	1	0.72	0.29	.59	0.00
Friends * Possessions	1	1.26	0.51	.48	0.00
Body * Psychology * Memory	1	0.01	0.00	.95	0.00
Body * Psychology * Friends	1	4.54	1.83	.18	0.00
Body * Psychology * Possessions	1	0.54	0.18	.68	0.00
Body * Memory * Friends	1	0.29	0.12	.77	0.00
Body * Memory * Possessions	1	2.64	1.06	.30	0.00
Body * Friends * Possessions	1	1.47	0.59	.44	0.00
Psychology * Memory * Friends	1	2.55	1.03	.31	0.00
Psychology * Memory * Possessions	1	0.00	0.00	.97	0.00
Psychology * Friends * Possessions	1	0.33	0.13	.71	0.00
Memory * Friends * Possessions	1	7.65	3.08	.08	0.00
Body * Psychology * Memory * Friends	1	4.40	1.77	.18	0.00
Body * Psychology * Memory * Possessions	1	0.02	0.01	.94	0.00
Body * Psychology * Friends * Possessions	1	15.08	6.07	.01	0.01
Body * Memory * Friends * Possessions	1	1.21	0.49	.48	0.00
Psychology * Memory * Friends * Possessions	1	0.74	0.30	.59	0.00
Body * Psychology * Memory * Friends * Possessions	1	0.04	0.02	.90	0.00
Error	789	2.48			
Total	821				

Table N

ANOVA results for agreement with statement "You do not exist after the incident." in Study 2 (QII7) as dependent variable (complete five-factorial design, N=821)

Source	df	Mean Square	F	Sign.	Partial η^2
Intercept	1	2471.09	1060.58	<.001	0.57
Body	1	0.09	0.04	.84	0.00
Psychology	1	0.46	0.20	.66	0.00
Memory	1	0.55	0.24	.63	0.00
Friends	1	7.31	3.14	.08	0.00
Possessions	1	5.12	2.20	.14	0.00
Body * Psychology	1	13.60	5.84	.02	0.01
Body * Memory	1	8.98	3.86	.05	0.01
Body * Friends	1	4.11	1.76	.18	0.00
Body * Possessions	1	0.72	0.31	.58	0.00
Psychology * Memory	1	52.04	22.34	<.001	0.03
Psychology * Friends	1	6.43	2.76	.10	0.00
Psychology * Possessions	1	2.83	1.21	.27	0.00
Memory * Friends	1	9.38	4.02	.05	0.01
Memory * Possessions	1	0.77	0.33	.56	0.00
Friends * Possessions	1	3.17	1.36	.24	0.00
Body * Psychology * Memory	1	0.35	0.15	.70	0.00
Body * Psychology * Friends	1	0.48	0.21	.65	0.00
Body * Psychology * Possessions	1	0.92	0.40	.53	0.00
Body * Memory * Friends	1	0.02	0.01	.92	0.00
Body * Memory * Possessions	1	0.65	0.28	.60	0.00
Body * Friends * Possessions	1	0.01	0.00	.95	0.00
Psychology * Memory * Friends	1	0.14	0.06	.80	0.00
Psychology * Memory * Possessions	1	1.59	0.68	.41	0.00
Psychology * Friends * Possessions	1	3.09	1.33	.25	0.00
Memory * Friends * Possessions	1	2.36	1.01	.31	0.00
Body * Psychology * Memory * Friends	1	1.37	0.59	.44	0.00
Body * Psychology * Memory * Possessions	1	0.05	0.02	.88	0.00
Body * Psychology * Friends * Possessions	1	0.49	0.21	.65	0.00
Body * Memory * Friends * Possessions	1	0.93	0.40	.53	0.00
Psychology * Memory * Friends * Possessions	1	9.60	4.12	.04	0.01
Body * Psychology * Memory * Friends * Possessions	1	2.86	1.23	.27	0.00
Error	789	2.33			
Total	821				

2.2.2. Decision difficulty and split psychology and memory. Figure N illustrates the interaction of the distribution of memory and psychology in predicting the degree of stated difficulty of the inheritance decision. The means for united conditions (B keeps psychology and memory, or B does not keep memory and does not keep psychology) are lower than for split conditions. This disordinal interaction is in line with the suggestion that it is easier to decide for a continuer when the components are united in one continuer.

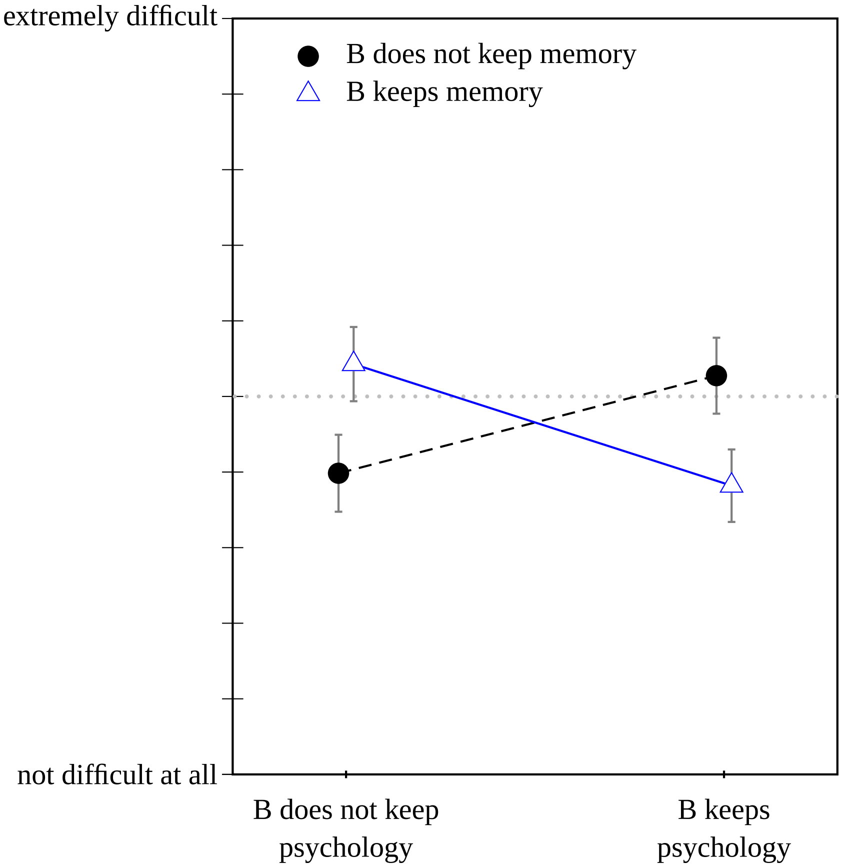

Figure N. Mean response to the question "'How difficult was it for you to decide who should be declared the heir?"' in Study 2 for the four possible distributions of memory and psychology component. Whiskers denote the 99% confidence intervals around the mean (N=821).

2.2.3. Inter-correlations of postquestionnaire responses. Specific correlations between items in the identity and survival importance scales (QII19–QII37) are discussed in the manuscript. Here the most relevant inter-correlations are presented in Table O for the identity items ordered by mean assigned importance: Correlations are shown for all of the 24 attributes with the eight sub-components of the five attributes featured in both studies.

The separate evaluation makes it possible to compare the ratings for otherwise joint dimensions: Appearance and body have similar mean ratings and are highly correlated ($r = .76$,

Table O

Selected inter-correlations for the 24 items concerning the importance of attributes for identity between people (QII9–QII32) in Study 2

	Importance for identity between persons							
Item	Bod	App	Per	Psy	Mem	Kno	Fri	Pos
**Memories (Mem)**	.09**	.04	.35***	.32***	—	.37***	.10**	-.11**
**Personality (Per)**	.10**	.11**	—	.53***	.35***	.37***	.08*	-.05
Mind	.07	.02	.44***	.58***	.39***	.31***	.07	-.04
Brain	.17**	.17**	.45***	.47***	.33***	.40***	.11	.03
**Knowledge (Kno)**	.15***	.12***	.37***	.34***	.37***	—	.17***	.06
**Psychology (Psy)**	.12***	.04	.53***	—	.32***	.34***	.07*	-.13
Moral values	.14*	.17**	.36***	.36***	.28***	.31***	.31***	.16**
Loved ones	.25***	.27***	.27***	.19**	.29***	.30***	.47***	.20***
Family	.26***	.27***	.17**	.05	.29***	.20***	.49***	.18**
Philosophy of life	.09	-.03	.33***	.42***	.22***	.31***	.19***	.03
Virtues and vices	.06	.02	.26***	.43***	.18**	.30***	.20***	.11
Gender	.43***	.43***	.16**	.07	.16**	-.02	.23***	.20***
Relationships w. o. ppl.	.27***	.14*	.19***	.14*	.10***	.20***	.56***	.26***
**Appearance (App)**	.76***	—	.11**	.04	.04	.12***	.16***	.32***
**Body (Bod)**	—	.76***	.10**	.12***	.09**	.15***	.21***	.33***
Private roles	.21***	.17**	.17**	.24***	.15**	.25***	.29***	.33***
**Friends (Fri)**	.21***	.16***	.08*	.07*	.10**	.17***	—	.33***
Religion	.11	.07	.02	.12*	-.05	.11	.33***	.21***
Profession/Job	.30***	.27***	.16**	.16**	-.05	.20***	.39***	.52***
Professional roles	.29***	.22***	.15*	.16**	-.07	.26***	.32***	.51***
Nationality	.43***	.43***	.08	.09	.05	.11*	.33***	.40***
Colleagues	.27***	.25***	.04	.11	.02	.06	.58***	.51***
Membership in groups	.22***	.22***	-.01	.07	-.13*	.06	.42***	.53***
**Possessions (Pos)**	.33***	.32***	-.05	-.01	-.11**	.06	.33***	—

$300 < n < 816$, *** p<.001 ** p<.01 * p<.05

$p < .001$). Similarly, personality and psychology ratings are correlated ($r = .53$, $p < .001$) and, to a lesser degree, memory and knowledge ratings ($r = .37$, $p < .001$), and personality and knowledge ratings ($r = .37$, $p < .001$).

Brain and mind are considered important for determining identity but show a high overlap with the dimensions we used in our scenario: Brain ratings correlated with psychology ($r = .47$, $p < .001$) and personality ($r = .45$, $p < .001$), and to a somewhat lesser degree with memory ($r = .33$, $p < .001$) and knowledge ($r = .40$, $p < .001$), but only weakly with body and appearance (both $r = .17$). Mind ratings correlated strongly with psychology ($r = .58$, $p < .001$) and personality ($r = .44$, $p < .001$), also with memory ($r = .39$, $p < .001$) and knowledge ($r = .31$, $p < .001$), but not significantly with body and appearance. These findings indicate that participants interpret body in terms of its visible properties, as the brain is attributed qualities that are not attributed to the body. At the same time, there was a strong correlation between gender and both body and appearance (both $r = .43$, $p < .001$); correlations between gender and other attributes were weaker, though there were positive correlations for both friends ($r = .23$, $p < .001$) and possessions ($r = .20$, $p < .001$).

Table P shows selected inter-correlations for the identity items (QII19–QII32) with the five survival items (QII33-QII37). Columns have been restricted to sub-components of the five central attributes.

Table P

Correlations between the 24 items concerning the importance of attributes for identity between people (QII9–QII32) and the five items concerning the importance of attributes for survival (QII33–QII37) in Study 2

	Importance for survival				
Item	Body	Psychol.	Memory	Friends	Possess.
**Memories**	.08*	.29***	**.57*****	.06	-.11**
**Personality**	.09*	**.54*****	.32***	.08*	-.03
Mind	.12*	.46***	.34***	.05	-.12*
Brain	.15**	.35***	.23***	.06	.03*
**Knowledge**	.12***	.29***	**.37*****	.13***	.04
**Psychology**	.11*	**.49*****	.26***	.05	-.05
Moral values	.01	.21***	.29***	.26***	.02
Loved ones	.23***	.09	.19***	.35***	.14*
Family	.12*	.05	.24***	.42***	.15**
Philosophy of life	.11	.24***	.14*	.18**	.02
Virtues and vices	.10	.17**	.15**	.18**	.07*
Gender	.34***	.07	.08	.22***	.17**
Relationships w. o. ppl.	.20***	.13*	.02	.52***	.15*
**Appearance**	**.55*****	.07	.03	.15***	.26***
**Body**	**.58*****	.06	.07*	.17***	.25***
Private roles	.19***	.22***	.14*	.27***	.23***
**Friends**	.18***	.02	.09*	**.62*****	.27***
Religion	.10	-.01	-.03	.27***	.14*
Profession/Job	.29***	.05	.04	.28***	.38***
Professional roles	.32***	.16**	.00	.28***	.37***
Nationality	.34***	.08	-.02	.31***	.30***
Colleagues	.26***	.04	-.03	.36***	.41***
Membership in groups	.27***	.01	-.18***	.38***	.40***
**Possessions**	.28***	-.03	-.10**	.31***	**.59*****

$300 < n < 816$, *** p<.001 ** p<.01 * p<.05

2.2.4. Post-questionnaire differences between studies. Changing the task from a continuous splitting task to an all-or-nothing task might change the interpretation of the underlying situation, as one anonymous reviewer suggested. If money is to be shared between continuers, this might be considered a suggestion by the experimenter that both continuers deserve some of the money by virtue of being related to the original person. This cueing might lead participants to change their assessment of the situation. In both studies we asked participants to answer questions regarding the state of the original person after the incident. A comparison of answers to these questions allows to measure the strength of this effect.

The original scale used was in the interval between 1 and 5 (Q3–Q8), the new scale between 0 and 5 (QII3–QII8) with the same anchor point labels at the extremes. To make values comparable, old answers were transformed by $x' = (x-1)\frac{5}{4}$. Table Q shows the mean responses, compared between both studies. Results are indeed consistent with a cueing effect:

Participants in Study 2 agree more with items expressing the survival and continuity after the incident and less with items expressing non-existence. Similarly, the maximum identification with one continuer is higher, the minimum identification lower. There is a smaller difference for the statement referring to being two people after the incident.

While the avoidance of a splitting task reduces the tendency to violate transitivity, it does not eliminate it and the analysis for Study 1 remains valid. The scatterplot in Figure I could still be produced for Study 2, for example. If psychology and memory are united in A, the mean identification rating with A and B is 3.34 and 1.31 (51.2% of answers are between 0 and 1), respectively. If they are united in B, the mean identification ratings are 1.23 (52.5% of answers between 0 and 1) and 3.33, respectively. But when memory and psychology are split and A keeps the memory the values are 2.58 and 2.21 (27.8% of the values between 0 and 1), when B keeps the memory the values are 2.30 (21.2% of the values between 0 and 1) and 2.50. These values are still comparable to those in Study 1.

Table Q

Test of cueing effects, Study 1 and 2

		Study1	Study2	Difference in means
You have survived the incident.	M	2.92	3.42	***
	SD	(1.43)	(1.46)	
You are the same person as before the incident.	M	1.67	2.02	***
	SD	(1.37)	(1.62)	
You are person A/B (max).	M	3.01	3.52	***
	SD	(1.32)	(1.23)	
You are person A/B (min).	M	1.40	1.18	***
	SD	(1.09)	(1.04)	
You do not exist after the incident.	M	1.98	1.73	**
	SD	(1.63)	(1.55)	
You are two people after the incident.	M	2.27	2.18	
	SD	(1.67)	(1.60)	

$N_1 = 821, 690 < N_2 < 696$ *** p<.001 ** p<.01 * p<.05

2.2.5. Order effects for survival items. In Study 1, questions concerning survival (Q14–Q18) were always asked after questions concerning the importance of dimensions for identity (Q9-Q13), which are more directly relevant for the article's focus. Varying the order between the blocks of questions (QII9–QII32 and QII33–QII37) systematically gave us a chance to test a potential effect of this forced ordering on the second block of questions.

Table R shows means and standard deviations for the five survival questions in Study 1 and in Study 2 (here, conditional on the relative position of the block). Tests for significant differences between the three conditions show that there are some changes between Study 1 and Study 2 (friends are considered more important in Study 2) and some differences between responses obtained in a block before identity questions and in a block after identity questions. Differences are small, though. Friends are considered slightly more important on average when asked before the identity questions, but slightly less important when asked after. This could, for

example, be caused by a tendency to interpret the second block of questions as non-redundant: Participants might try to demonstrate how determining survival is different from determining identity between people and see the body as more central and memory as less central when survival questions are framed in this way. As ratings for body and friends are close to each other in all conditions and those for memory far higher, this potential effect of order does not impact other analyses or their interpretations.

Vice versa, the ordering of the block of questions concerning identity does not seem to impact responses at a statistically significant level, with the exception of two items, that are only significant if p-values are not adjusted: These are for gender ($t(305) = 2.35$, $p = .02$, two-tailed test) and Friends ($t(815) = 2.00$, $p = .05$, two-tailed test). In both cases, importance for identity is considered lower when asked after the survival questions. The difference in means is smaller than one scale point on a scale from 0 to 10, and there were 24 significance test, which raises doubts about interpretability.

Table R

Test of order effects for survival weightings (QII33-QII37), Study 1 and 2

		Study1	Study2		Difference in means		
		(a) after	(b) before	(c) after	ab	ac	bc
Survival: Body and appearance	M	4.52	4.18	4.62			*
	SD	(3.20)	(3.00)	(3.07)			
Survival: Personality and psychology	M	7.79	8.05	7.75			
	SD	(2.48)	(2.19)	(2.30)			
Survival: Memory and knowledge	M	8.01	8.55	8.08	***		**
	SD	(2.39)	(2.02)	(2.30)			
Survival: Friends	M	3.69	4.34	4.26	***	**	
	SD	(2.95)	(3.13)	(3.08)			
Survival: Possessions	M	2.17	2.07	2.49			*
	SD	(2.60)	(2.36)	(2.63)			
N		704	424	397			

*** p<.001 ** p<.01 * p<.05

2.2.6. Reductionist and non-reductionist belief among participants. The thought scenario used in both studies demands a stretch of imagination, as it is implicitly assumed that the five chosen dimensions can be separately allocated to different continuers. This assumption is shared by some of the earliest thought experiments, such as the story of the prince and the cobbler (Locke, 1700/1998): the prince is able to perceive himself as the prince while in the cobbler's body. For those striving to reduce psychology to physiology or physics, this assumption might well prove impalatable. While we did not receive any complaint by participants, several questions were added at the end of the survey to assess to which degree these scenario elements conflict with espoused attitudes towards the relationship between body and mind. Table S summarizes mean responses and frequencies of positive and extreme answers to the ten survey questions (QII38–QII47).

Results show that participants were roughly split into equal parts in affirming and dis-

Table S
Mean responses, standard deviations and relative frequency percentages of positive and high and low responses for questions regarding interdependence of body and mind (QII38–QII47)

	Descriptives		Relative frequency		
	M	SD	>5	<3	>8
Nothing in the mind occurs that does not... ...originate in the body.	5.62	2.86	52.2	19.3	19.8
Everything that we think or feel can be reduced to... ...activations of neurons in our body.	5.35	2.87	49.2	19.7	20
Ghosts truly exist in this world	5.51	2.86	49.2	19.7	20
At least in theory psychology can be reduced to physics.	5.52	2.91	50.2	20.6	20
Body and mind are independent to some relevant degree.	5.49	2.92	49.3	20.6	21.2
Humans have souls that are separate from their bodies.	5.65	2.75	52.6	16.3	18.7
In the future it may be possible to overcome death... ...by uploading your mind to a computer.	5.41	2.95	49.4	21.8	21.3
I like the current state of my body and appearance.	5.38	2.87	47.5	21.4	17.9
I like the current state of my memories and knowledge.	5.44	2.84	49.7	19.7	20.2
I would like to be somebody else.	5.62	2.89	51.2	18.8	22.4

affirming each of the statements, with about 20% strongly affirming and 20% strongly disaffirming each of the statements. This indicates, for example, that nearly half of the participants believe in the existence of ghosts, and most participants believe in the existence of souls separate from bodies. While these endorsement do not align with the standard scientific understanding of the world, they at the same time demonstrate that conceptual difficulties with the basic premises of our scenario should have been limited to few participants.

The last three questions are different and relate to the perspective of participants on their own attributes. Both body/appearance and memory/knowledge were positively regarded by less than half of the participants in their current state and most participants would wish to be somebody else. Again, these responses demonstrate that the notion of alternative versions of the self was not incompatible with participants' other beliefs. Also, dissatisfaction with being oneself is compatible with the explanation given in 2.1.7 for the negative evaluation of specific dimensions (assuming that participants in Study 1 would have given similar responses to those in Study 2).

2.2.7. Cluster analyses of postquestionnaire items: Explicit weighting of dimensions. In Study 2, participants were asked to weight sub-dimensions separately, that is, they gave a separate rating for body and appearance and similarly, for psychology and personality as well as memories and knowledge. Average evaluations did not show substantial differences in sub-dimension ratings. To explore, whether this result holds for subgroups and to conceptually replicate the cluster analysis for Study 1 (see 2.1.9), we conducted a new cluster analysis on the eight ratings for dimensions with IBM SPSS 24.0, a k-means cluster analysis with six clusters (we kept the number constant to allow for a replication in theory). In cases where we found a close enough match between found clusters and clusters in the first analysis, we used the same

letter to designate the new cluster, this resulted in clusters A_2, B_2, D_2, and F_2. We used new letters for the final two clusters. The final cluster centers are visualized in Figure O:

- Cluster A_2 ($n_{A_2} = 197$, 24.1%) is comparable to cluster A in that all dimensions are assigned importance. The only difference is a slightly lower rating for possessions.
- Cluster B_2 ($n_{B_2} = 193$, 23.6%) is comparable to cluster B, with slightly higher ratings for friends. Psychology and memory are assigned very high ratings, other ratings are much lower.
- Cluster D_2 ($n_{D_2} = 103$, 12.6%) is comparable to cluster D, as there is no dimension with particularly high ratings, but slightly lower ratings for friends and possessions.
- Cluster F_2 ($n_{F_2} = 155$, 19.0%) is comparable to cluster F, with high ratings for the first three dimensions, but low ratings for friends and possessions.
- Cluster G_2 ($n_{G_2} = 70$, 8.6%) is very similar to cluster B_2, the importance of psychology and memory is about two points lower.
- Cluster H_2 ($n_{H_2} = 99$, 12.1%) has the highes values for memory, and shows more variance between sub-dimensions: There is a three point difference between personality (rated higher) and psychology, two points in favor of appearance over body and again two points in favor of memories over knowledge. The latter difference is also observed in cluster G_2.

There is no direct equivalent to cluster C (focus on psychology, memory, and friends) and cluster E (focus on the first four dimensions) from Study 1 (see 2.1.9)in the first analysis, but note that the analysis was done on a different set of questions with a larger weight on the first three dimensions due to double entries. The friends category is therefore likely to be technically suppressed. Differences between sub-dimensions are small for most groups. Beyond the differences mentioned for cluster G_2 and H_2 all other differences are well below two points, which again validates the grouping chosen in the scenario. Again, we observe relative unanimity in considering psychology and memory important with split ratings for body and appearance.

2.2.8. Cluster analyses of postquestionnaire items: Re-identification after fission. Study 2 offers a chance to directly test whether the cluster structure found in Study 1 re-emerges in this new group of participants. We decided against a confirmatory approach, as the change in the scenario question might create qualitative differences. Instead, we ran another cluster analysis with IBM SPSS 24.0, a k-means cluster analysis with six clusters (again we kept the number constant to allow for a replication in theory). The cluster centers are shown in Figure P.

To allow for ease of comparison, we re-ordered the clusters (the initial order is after all arbitrary) so that clusters of the first analysis can be compared with clusters in the new analysis. Where a qualitative similarity was found, we used the letter of the original cluster,

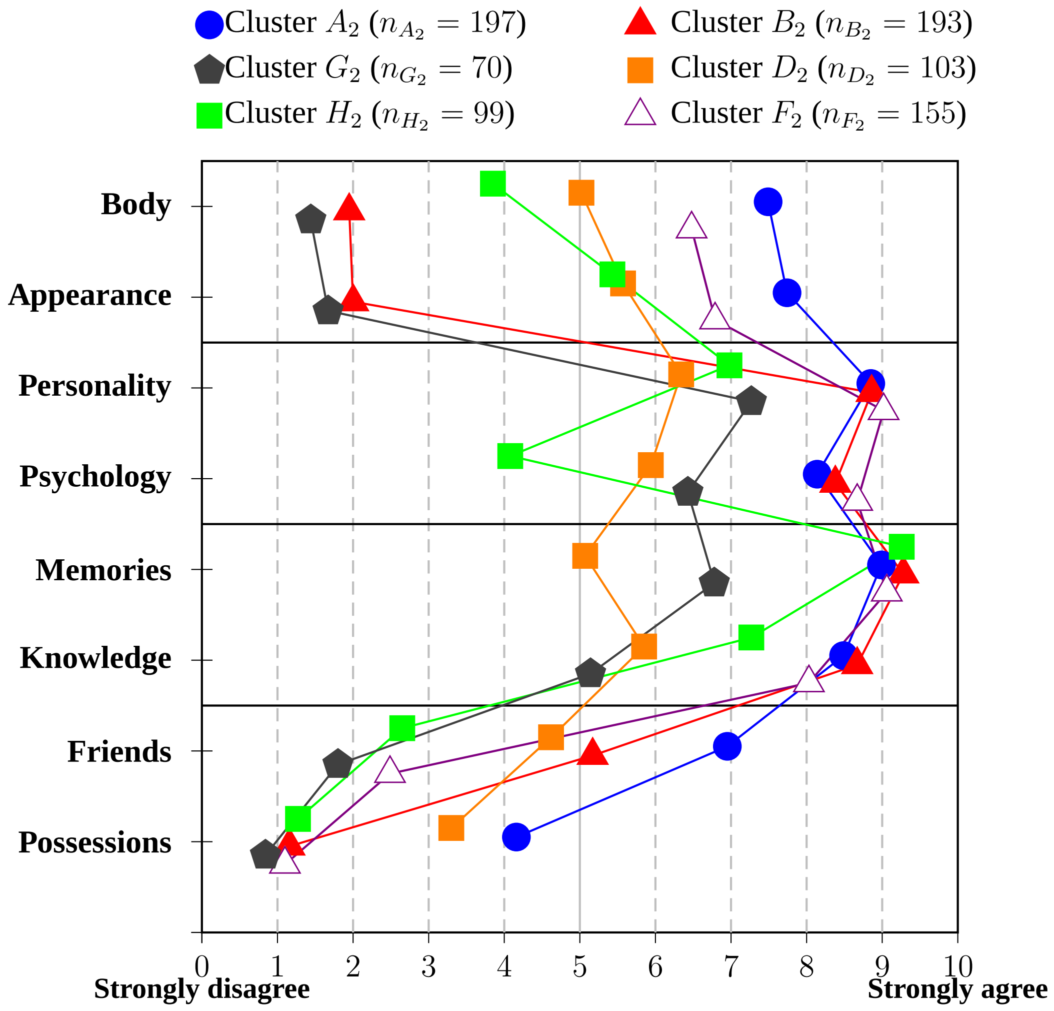

Figure O. Cluster centers after final iteration for a cluster analysis of the eight attribute evaluations for identity in Study 2

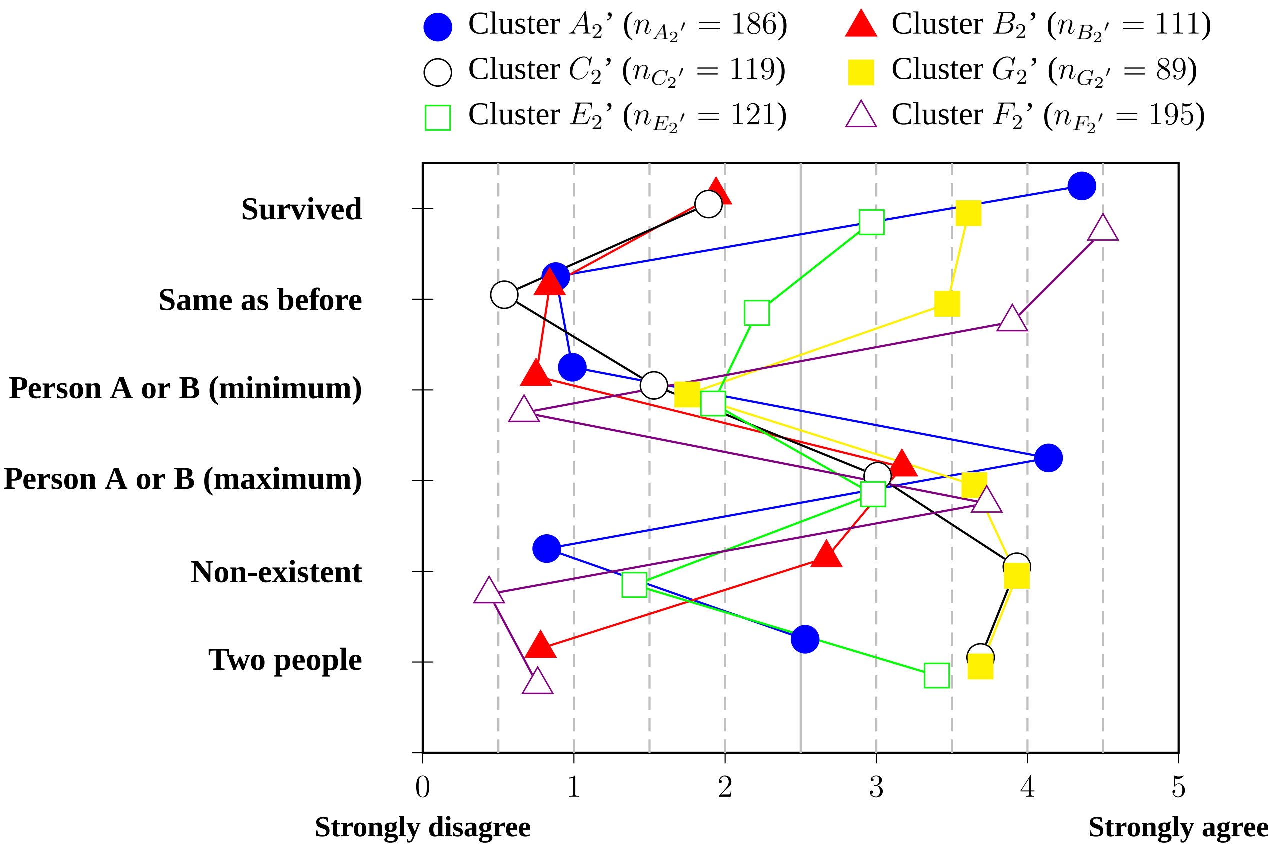

Figure P. Cluster centers after final iteration for a cluster analysis of the six postquestionnaire items in Study 2

indexed with 2. In this fashion, we were able to find a correspondence, for the cluster A', B', C', E', and F' of Study 1. The final cluster shows a different pattern from the original cluster D and no close similarity with any other previous cluster, therefore it was named G_2'. The correspondence varies in degree somewhat, cluster C' and cluster F', and possibly cluster E' show reasonably close matches with their corresponding clusters, cluster B_2' has a higher maximum identification and survival value compared to B'. Cluster A_2' shows relatively more extreme average answers to the first two questions, and less positive responses to the final two questions. The cluster solutions still show a high degree of similarity. For this reason, the new cluster descriptions would roughly correspond to those in Study 1 (see 2.1.10), with the exception of cluster G_2': Participants in this smallest cluster give similar answers as those in cluster C_2', but see themselves as both surviving and unchanged (while also confirming to be two people and non-existent). One may wonder, whether this pattern emerges from a response style of acquiescence or expresses a unique perspective on the situation.

Table T

Differences between clusters in average decision difficulty and scenario conditions in Study 2

	decision difficulty	larger attribute share	proportion memory/ psychology united
Cluster A_2'	4.6	67.4%	46.8%
Cluster B_2'	4.3	68.1%	49.6%
Cluster C_2'	5.9	65.2%	31.9%
Cluster G_2'	5.1	71.0%	51.7%
Cluster E_2'	5.1	66.9%	48.8%
Cluster F_2'	3.5	72.9%	65.6%

Study 2 also allows us to confirm the relationships with scenario features. As there is no monetary allocation in Study 2, we report the average perceived decision difficulty in Table T. Again, participants in the six clusters differ significantly in terms of decision difficulty ($F(5, 815) = 13.05$, $p < .001$), maximum share in attributes ($\chi^2(10) = 52.7$, p<.001), and separation of psychology ($\chi^2(5) = 35.5$, $p < .001$). Decision difficulty here stands in a similar relation to the clusters as monetary allocation in Study 1 (of course, the direction of the relationship is reversed, more extreme allocations correspond in this sense to a lower decision difficulty). For this reason, we refer you to section 2.1.10., as the interpretation of the new results would run in parallel with the one given for Study 1.

2.2.9. Percentage of traits retained and postquestionnaire responses. The percentage of dimensions retained in a person varied form 0% to 100% in 20%-steps. Here we analyze the answers to the postquestionnaire items separated by percentage of attributes retained. For the four items that are not person-specific we would expect a vertical symmetry (an 80%-20% split in favor of A is equivalent to a 20%-80% split for B. We both plot the means across percentages and conduct one-factorial ANOVAs with linear and quadratic contrast terms (to test for the expected symmetry).

Responses to the two person-specific questions concerning the identification with either

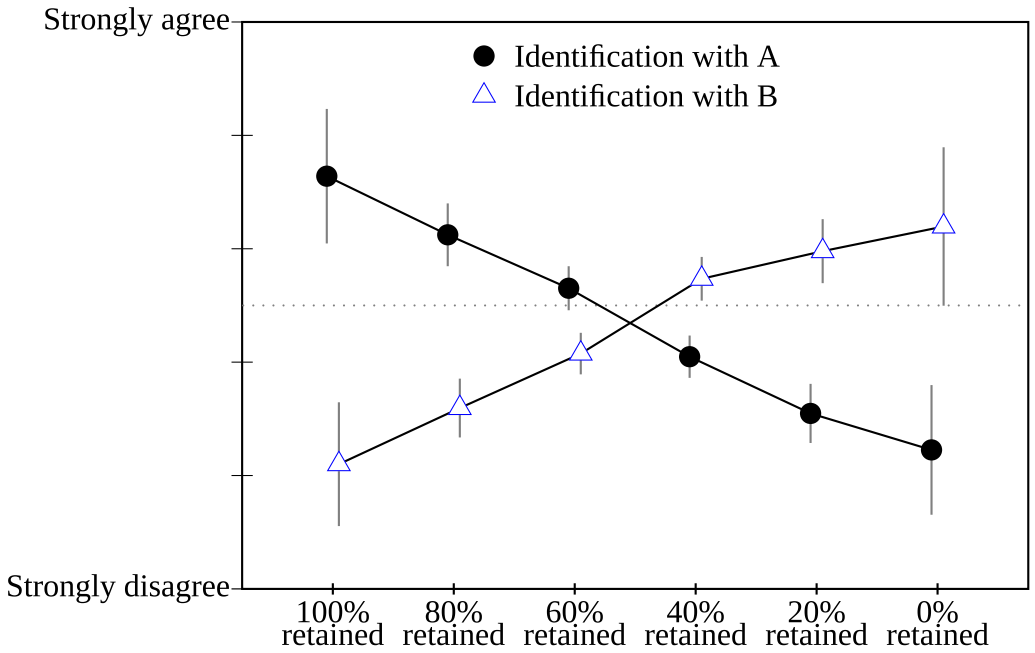

Figure Q. Mean maximum and minimum response to the statements "‘You are Person A after the incident."’ and "‘You are Person B after the incident."’ for the all possible percentages of retained dimensions in Person A. Whiskers denote the 95% confidence intervals around the mean (N=821).

continuer are plotted in Figure Q. There is a significant effect of percentage retained both for identification with Person A ($F(5, 815) = 23.85$, $p < .001$) and Person B ($F(5, 815) = 20.19$, $p < .001$) with significant linear terms ($F(1, 815) = 61.62$, $p < .001$; and $F(1, 815) = 47.63$, $p < .001$; unweighted). Identification with either person tracks the percentage of dimensions retained in that person. Note that this result does not imply that participants use a simple tallying heuristic: the percentage of traits retained also determines the probability that any given trait or a combination of traits are retained in a person. On the other hand, the difference from the extreme values for the categories 0% and 100% show an effect of the incident that goes beyond the disruption of continuity: Even if one of the two continuers retains all traits, there is only an imperfect identification on average with this continuer and still some residual identification with the other continuer in the sense, that participants are at least not completely certain that they are one and not the other.

The responses to the questions regarding survival and being the same person follow a similar pattern, as seen in Figure R. Both responses show a significant effect of percentage retained ($F(5, 815) = 4.62$, $p < .001$ and $F(5, 815) = 10.84$, $p < .001$) with a significant quadratic term ($F(1, 815) = 15.61$, $p < .001$; and $F(1, 815) = 47.18$, $p < .001$); unweighted). The comparison shows that participants are more willing to accept to have survived the incident

than to be unchanged by the experience. This is most pronounced for splits that leave continuers with a similar number of dimensions. This also demonstrates that the closeness metric is more heavily impacted by scenario conditions than the survival judgments, which coincides with the prediction of the closest continuer theory: As long as one of the continuers meets the threshold, survival would be guaranteed (no matter how far above the threshold this continuer is). Survival would be only endangered if the threshold is not met by either continuer or both continuers are too close. Both of these conditions are most likely to be expected in the 60% and 40% conditions.

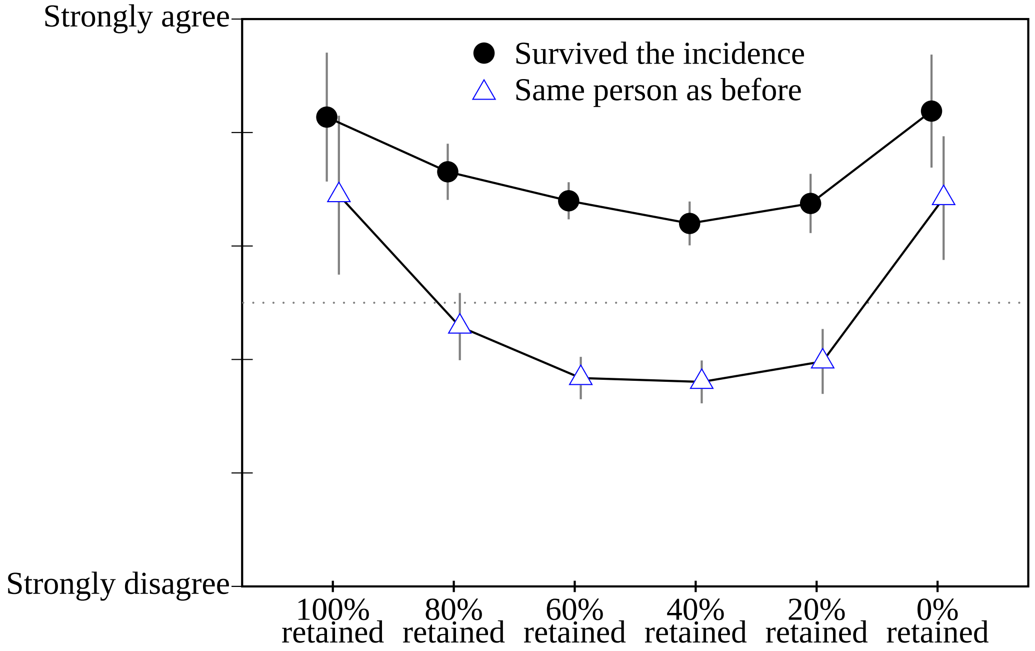

Figure R. Mean response to the statements "‘You have survived the incidence."’ and "‘You are the same person as before the incident."’ for all possible of percentages of dimensions retained in Person A. Whiskers denote the 95% confidence intervals around the mean (N=821).

Answers to the two-persons and non-existence questions are plotted in Figure S. Again, both responses are significantly influenced by the percentage retained ($F(5, 815) = 4.75$, $p < .001$ and $F(5, 815) = 3.35$, $p = .005$), again with significant quadratic terms ($F(1, 815) = 6.98$, $p = .008$; and $F(1, 815) = 8.73$, $p = .003$), but with an inverse order of responses compared to the survival questions. Participants agree more with the notion to be two people after the incident than not to exist at all in all conditions. Both responses track the percentage of traits retained, but to a more limited degree than earlier questions. Again, it seems that the two reactions are alternative (and as seen in 2.1.10 sometimes complementary) reactions to situations, in which both continuers are similar to each other and both lack dimensions. At the same time, on average participants do not completely reject these options, even when 100% of

dimensions are retained in either continuer. The incident itself seems to have some influence on triggering these responses.

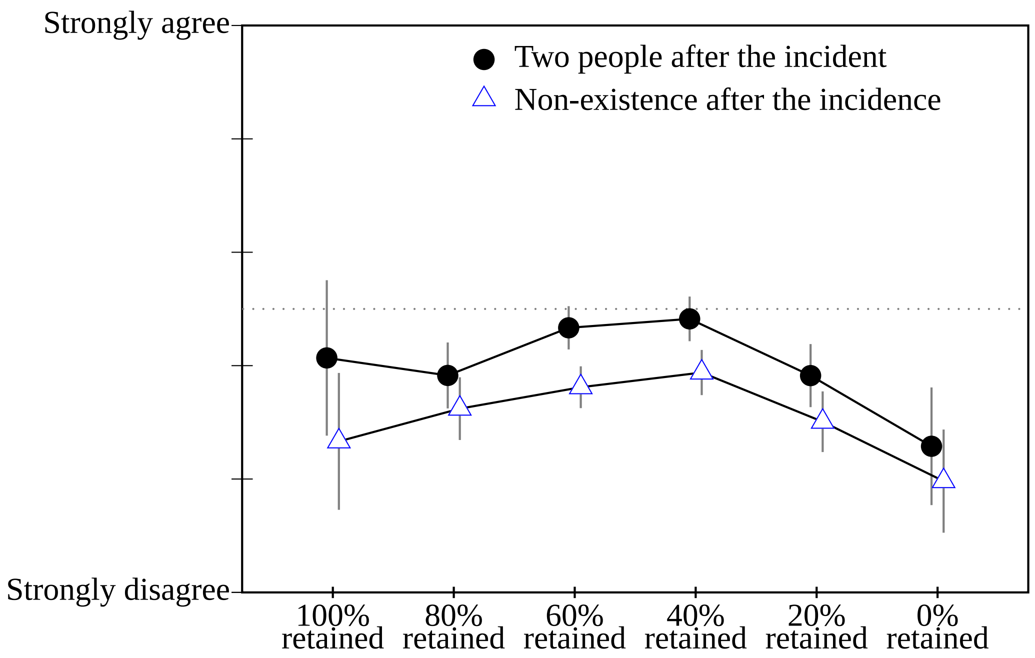

Figure S. Mean response to the statements "‘You are two people after the incident."’ and "‘You do not exist after the incident."’ for all possible of percentages of dimensions retained in Person A. Whiskers denote the 95% confidence intervals around the mean (N=821).

2.3. Qualitative Analysis in Study 1: Reasons given for allocating the money

Our participants were asked to justify the allocation of the money in an open answer format. A minimum number of characters was enforced, which virtually all participants used to give answers consisting of explanatory sentences. We analyzed these answers for two main reasons: (1) These answers may contain confirmatory evidence for supporting our analysis of the decision process. (2) These answers might give indication that some participants might have reinterpreted the scenario or might have allocated money in a way that could invalidate our conclusions. The classification was done on the basis of the statements, the splitting condition and the monetary allocation, as subjectively judged by the first author. The found categories are not necessarily clear-cut and exclusive, but are meant to give an impression of the type of variation in participants’ judgments.

2.3.1. Confirmatory evidence. Of 704 total responses, $n_c = 521$ (74.0%) were considered to follow the intended logic of the scenario. Table U shows the distribution of justification types across participant groups with similar allocation patterns. Participants that allocated the

money evenly between continuers were found to give different types of justification than the other two groups. Each type of justification is illustrated by some prototypical examples produced by participants (all statements are shown exactly as submitted without any corrections). All percentages are relative to n_c.

Justifications for equal allocations. The largest group of participants among those that split the money evenly (16.1% of all responses that follow the logic of the scenarios), were those arguing for a fundamentally similar situation for both continuers, which did not justify to prefer one over the other:

> *Participant # 236*: Both the surviving people will have been irreparably harmed in the same way and should at least be able to equally share the payment.

The second largest subgroup (13.8%) expressed genuine indifference between continuers based on a comparison of their attributes. This indifference was typically motivated by a weighing of attributes on each side:

> *Participant # 238*: I feel they are even. My personality and psychology as opposed to my memories and knowledge. Equally important in making up "me".
>
> *Participant # 296*: It's only fair. I like my possessions and my body just as much as I like my mind. If we must split, it's gonna be down the middle.

Others in this group gave a lack of connection with either continuer as reason for their indifference:

> *Participant # 425*: I wanted to be fair to the two since when I cease to exist, everything related to me doesn't matter to me and I want both of them to be able to benefit from me being gone. I want my money to be able to affect two lives rather than play favorites.

A third group invoked the fairness norm explicitly in justifying their allocation (10.2%). All of them used the word "fair" or referred to "fairness", such as:

> *Participant # 175*: Splitting it in half seemed the fairest thing to do.

A final group (7.3%) basically re-described their action of evenly allocating money as their goal or motivation for of their action:

> *Participant # 360*: I just tried to even out the money between the two.

Justifications for unequal allocations. The largest subgroup of participants (11.7%) described one of the continuers as the closer continuer of the two:

> *Participant # 205*: Person A is me, in any meaningful way. But Person B is also me, but just much less so me.
>
> *Participant # 320*: I would rather that someone that is more like me to get the money.

Table U

Distribution of justification type: Each row describes the number of occurrences of each justification type. Participants are split in those that allocate the money equally, unequally (minimum allocation higher than 25%) or extremely (minimum allocation below 25%). All percentages refer to the total number of justifications considered to follow the intended logic of the scenario (N=521).

	Justification	Equal split	Unequal split	Extreme split	Row-total
Equal	Equal compensation	84	—	—	84 (16.1%)
	Indifference between continuers	72	—	—	72 (13.8%)
	Fairness	53	—	—	53 (10.2%)
	Equal distribtuion	38	—	—	38 (7.3%)
	Total				*247 (47.4%)*
Non-equal	Continuer is my closer continuer.	—	33	28	61 (11.7%)
	Continuer has more important parts	—	16	26	42 (8.1%)
	Continuer is me	—	7	33	40 (7.7%)
	Continuer is "me"	—	7	30	37 (7.1%)
	Continuer has most important part	—	9	17	26 (5.0%)
	Continuer is the more attractive person.	—	10	6	16 (3.1%)
	Continuer is my closest continuer.	—	7	8	15 (2.9%)
	One continuer has more of me, the other less.	—	12	3	15 (2.9%)
	One continuer is me, the other deserves compensation.	—	2	10	12 (2.3%)
	Tallying of attributes	—	8	2	10 (1.9%)
	Total				*274 (52.6%)*
All	*Column-total*	*247 (47.4%)*	*111 (21.3%)*	*163 (31.3%)*	

Others (8.1%) focused on the differentiating attributes, and judged some of them as more important than others:

Participant # 257: my memories, personality, and friendships are more important to me than my body and possessions

Participant # 262: The mind and emotions are more important than the physical looks and possesions.

Another group of participants (7.7%) refer to one of the two continuers as themselves, using personal pronouns without any qualifications:

Participant # 156: Because Person B basically IS me, he only lost material possessions, everything else is basically the same. He has all of my memories, my personality, my knowledge, my friends. So I would want him to have it all because he would be me.

Participant # 664: Person B has my brain and therefore is exactly who I am.

Similarly, some participants (7.1%) refer to one of the two continuers as versions of themselves, but use some form of qualification or limitation:

Participant # 500: My memories, knowledge, and personality are almost my whole being.

Participant # 590: I would keep most of the money for me, that is the me that looks like a completely different person, because the core of me would be in that body.

A further group of answers (5.0%) focused on some attributes as the most important ones (without comparing them to less important ones):

Participant # 668: My personality and psychology are most important to me.

While the first group was described as describing one continuer as the closer continuer, a smaller group of participants (3.1%) either invokes the principle of the "closest continuer" or nominates one of the continuers to fill this role:

Participant # 447: So the one most like me gets the most.

A number of participants (2.9%) did not focus on relatedness but on attractiveness of the two continuers and considered one of the two to be more appealing or likeable.

Participant # 662: like Person A better. Dont have alot of friedns

Focusing on the allocation of compensation, some participants (2.9%) judge one continuer to be worthy of more compensation because that continuer has a stronger connection to the original, but stress that the weaker connection still entitles the other continuer to some part of the money:

Participant # 320: I gave person “A” a bit more money because that person has more of my traits. However, person “B” has my present personality and psychology, which I think are pretty important traits, so I gave person “B”almost the exact same amount as I gave to person A.

Participant # 523: I’d like the person that resembles me and contains my possessions to have the most compensation, but the person with personality is “me” as well, so they deserve some too.

In contrast to the former group, some participants (2.3%) allocate money to the second continuer not based on relatedness but argue with general desert or fairness:

Participant # 139: Person B is much more me, in every meaningful sense. The other person deserves some compensation too, though.

Participant # 480: I do believe that the other individual has been inconvenienced also, and so is entitled to some recompense – but then, he also gets all of my possessions. I think that I will certainly “be” person B, and that I am entitled to most of the payout in this cae.

Finally, the smallest category (1.9%) encompasses participants who basically counted the number of matching attributes and awarded compensation according to this tally:

Participant # 611: I gave two thirds to person B because they have three of the five qualities while person A only has two. 2/3 to 1/3 distribution.

2.3.2. Alternative interpretations. Of $N = 704$ total responses, $n_e = 183$ (26.0%) did not follow the intended logic of the scenario. Still, only a few of these answers were nonsensical or erroneous, many indicated legitimate interpretations or re-interpretations of the scenario, albeit often with a change in focus or meaning of the monetary allocation. Table V shows the distribution of justification types across participant groups with similar allocation patterns.

Table V

Distribution of non-standard justification type: Each row describes the number of occurrences of each justification type. Participants are split as in Table U. All percentages refer to the total number of justifications considered not to follow the intended logic of the scenario (N=183).

	Equal split	Unequal split	Extreme split	*Row-total*
Reverse allocation (compensation)	—	53	34	*87 (47.5%)*
Non-response or conceptual error	23	18	10	*51 (27.9%)*
Create (re-)interpretation of scenario	8	26	11	*45 (24.6%)*
Column-total	*31 (16.9%)*	*97 (53.0%)*	*55 (30.1%)*	

The most damaging (in terms of our research question), and yet the most frequent type of alternative answer types (47.5% of all alternative answers) did not take the original person as reference point for compensation, but argued from the point of view of the continuers. Often their state after creation was considered and the amount of suffering judged with the idea,

that both continuers needed to be compensated for their situation irrespective of the original. In this category, though, closeness to the original was judged to be preferable for similar reasons as expressed in answers above, yet the closer individual was judged to be *less* in need of compensation:

> *Participant # 49*: Well, I have a fairly good life and am willing to think that the person that gets my attributes will be able to go on and do well for themselves. The second person is getting a crap shoot. Who knows what they'll end up with. The only thing I know is tht they'll get my possessions, which I've pared back a bit since I am aging, and that include a ranch and farm animals, a lifestyle that not everyone likes or can handle. So they'll need some compensation to change situations if they need to.
>
> *Participant # 55*: It seems like person B underwent many more drastic changes than person A, so I felt that they should get more compensation.
>
> *Participant # 405*: Its clear that Person A has gone through more problems and therefore deserves a large settlement.
>
> *Participant # 638*: I chose to compensate person B more as they were pulled from their reality and dropped here, whereas person A is just me and will really have no different a life than they had before.

Strangely enough but maybe not surprising, some of the arguments brought forward in this category were rather self-centered, estimating the amount of suffering to be a function of the distance to themselves (the original).

A second group of answers (27.9% of alternative answers) could not be interpreted as valid responses to the scenario, either because the answer was too brief or implied a serious misunderstanding or hard to explain alternative reading of the scenario:

> *Participant # 25*: Person A seems more reliable to insurance policy.
>
> *Participant # 407*: B seems to be the odd choice out which appeals to me for travel.
>
> *Participant # 703*: just what I belive

The smallest group in this category (24.6%) offered creative readings of the scenario, such as:

> *Participant # 20*: I was going to split it fifty-fifty but then I remembered person B gets all the possessions, which includes, at least, a personal hyperspace car. Or rocket?

Some members of this group focused on specific properties of memory that interact with the scenario:

> *Participant # 547*: The person with the present memories and knowledge would be more traumatized by the event.
>
> *Participant # 592*: Well since person B is the only one going to remember the situation I think he should be compensated not to mention person A end up with ridiculously good looking body so I think that is compensation enough for this mishap.
>
> *Participant # 601*: I think A should receive more because A has the present memories and knowledge, so A is more aware of what occured and more likely to be affected by it.

In a similar vein, some participants estimate a differential capability to make use of the money, or estimated need from the current (not an assumed future) state of possessions:

> *Participant # 349*: I have no connection to person B, but I do feel bad that they are being impacted by this as well. Then again, I have no connection to person A either but I feel like since we look exactly the same and have the same possessions, person A could really use te money to buy some better looking clothes and maybe a new car. But they are both being inconvenienced so they both get money.
>
> *Participant # 490*: I would give most money to the person with my psychology, because I feel I am good with money. Some would still go to a person with my memories because I have good knowledge with money.
>
> *Participant # 671*: If person A has my present possessions and personality, I want to be sure they can live more comfortably than I live. With my memories and knowledge, hopefully person B will end up with better possessions and can use my knowledge to earn more money for thmselves.

2.3.3. Conclusion. The range of explanations and the level of detail are taken as a whole an indication of diligence in answering the scenario questions on the side of the participants. There are very few responses in line with a satisficing account of working through the task. The pattern of answers confirms that equal allocations are for the most part (but not completely) motivated in a qualitatively different way, often invoking fairness and equality concepts. Also, most respondents react to the scenario in the intended way: Many participants implicitly or explicitly use the closest-continuer criterion or at least a closer-continuer criterion for assigning money, mirroring Nozick's criterion for determining personal identity. Others follow a more criterial approach for determining shares, many in both groups in line with a psychological theory of continuity.

The analysis also highlights aspects of the scenario that could be improved upon: a substantial number of participants focused less on the relationship between original and continuers but evaluated the state of the continuers independently. Nonetheless, these diverging responses do not offer an alternative interpretation of our results in the main text: Those respondents compensating the more similar continuer less introduce noise into the analysis and make it harder to detect effects for the attribute factors. Indeed, an exploratory ANOVA after eliminating participants with alternative readings results in larger effect sizes. A much weaker main effect for the friends-factor and two two-way interactions of similar smaller size surpass the significance threshold in this analysis. Due to the subjectivity involved in assigning labels to the responses, this analysis is not reported here. In addition, the vast majority of responses indicate a correct reading of the scenario with the alternative reading restricted to the allocation decision. Should the scenario be used in future studies, it might be recommended that the focus on the original protagonist is made stronger. In addition, a basic payment by the company to both continuers as a reflection of their suffering might be added so that perceived responsibility for the two continuers' ability to start their existence does not determine the allocations as observed in some cases.

References

Bench, S. W., Schlegel, R. J., Davis, W. E., & Vess, M. (2015). Thinking about change in the self and others: The role of self-discovery metaphors and the true self. *Social Cognition*, *33*(3), 169–185.

Berniūnas, R., & Dranseika, V. (2016). Folk concepts of person and identity: A response to Nichols and Bruno. *Philosophical Psychology*, *29*(1), 96–122. doi: 10.1080/09515089.2014.986325

Blok, S., Newman, G., Behr, J., & Rips, L. J. (2001). Inferences about personal identity. In *Proceedings of the annual meeting of the cognitive science society* (Vol. 23).

Campbell, S. (2005). Is causation necessary for what matters in survival? *Philosophical Studies*, *126*(3), 375–396. doi: 10.1007/s11098-004-7786-1

Chen, S. Y., Urminsky, O., & Bartels, D. M. (2016). Beliefs about the causal structure of the self-concept determine which changes disrupt personal identity. *Psychological Science*, *27*(10), 1398–1406. doi: 10.1177/0956797616656800

Clark, A. (1997). *Being there: Putting brain, body, and world together again*. Cambridge, MA: MIT press.

Cumming, G. (2014). *The new statistics: Estimation for better research.* Retrieved Aug 14, 2014, from http://www.thenewstatistics.com

Fields, C. (2012). The very same thing: Extending the object token concept to incorporate causal constraints on individual identity. *Advances in Cognitive Psychology*, *8*(3), 234–247. doi: 10.5709/acp-0119-8

Gottfried, G. M., Gelman, S. A., & Schultz, J. (1999). Children's understanding of the brain: From early essentialism to biological theory. *Cognitive Development*, *14*(1), 147–174.

Gutheil, G., Gelman, S. A., Klein, E., Michos, K., & Kelaita, K. (2008). Preschoolers' use of spatiotemporal history, appearance, and proper name in determining individual identity. *Cognition*, *107*(1), 366–380. doi: 10.1016/j.cognition.2007.07.014

Heiphetz, L., Strohminger, N., Gelman, S. A., & Young, L. L. (n.d.). Who am i? the role of moral beliefs in children's and adults' understanding of identity. *Journal of Experimental Social Psychology*. doi: 10.1016/j.jesp.2018.03.007

Heiphetz, L., Strohminger, N., & Young, L. L. (2017). The role of moral beliefs, memories, and preferences in representations of identity. *Cognitive Science*, *41*(3), 744–767. doi: 10.1111/cogs.12354

Henningsen, A. (2013). censreg: Censored regression (tobit) models [Computer software manual]. Retrieved from http://CRAN.R-project.org/package=censReg (R package version 0.5-20)

IBM Corp. (2011). IBM SPSS statistics for windows, version 20.0. [Computer software manual]. Armonk, NY.

Kitcher, P. (1979). Natural kinds and unnatural persons. *Philosophy*, *54*(210), 541–547. doi: 10.1017/s0031819100063567

Kolak, D., & Martin, R. (1987). Personal identity and causality: Becoming unglued. *American Philosophical Quarterly*, *24*(4), 339–347.

Locke, J. (1700/1998). *An essay concerning human understanding (Clarendon edition of the works of John Locke)* (New ed.; P. H. Nidditch, Ed.). Oxford, UK: Oxford University Press.

Matheson, B. (2014, Sep 01). Compatibilism and personal identity. *Philosophical Studies*, *170*(2), 317–334. doi: 10.1007/s11098-013-0220-9

Matthews, S. (2000). Survival and separation. *Philosophical Studies*, *98*(3), 279–303. doi: 10.1023/A:10186611

McMahan, J. (2002). *The ethics of killing: Problems at the margins of life*. New York: Oxford University Press.

Mills, E. (1993). Dividing without reducing: Bodily fission and personal identity. *Mind*, *102*(405), 37–51. Retrieved from www.jstor.org/stable/2254171

Molouki, S., & Bartels, D. M. (2017). Personal change and the continuity of the self. *Cognitive psychology*, *93*, 1–17.

Moyer, M. (2008). A survival guide to fission. *Philosophical Studies*, *141*(3), 299–322. doi: 10.1007/s11098-007-9161-5

Newby-Clark, I. R., & Ross, M. (2003). Conceiving the past and future. *Personality and Social Psychology Bulletin*, *29*(7), 807–818.

Newman, G. E., De Freitas, J., & Knobe, J. (2015). Beliefs about the true self explain asymmetries based on moral judgment. *Cognitive Science*, *39*(1), 96–125. doi: 10.1111/cogs.12134

Nichols, S., & Bruno, M. (2010). Intuitions about personal identity: An empirical study. *Philosophical Psychology*, *23*(3), 293–312. doi: 10.1080/09515089.2010.490939

Nozick, R. (1981). *Philosophical explanations*. Cambridge, MA: Belknap.

Nunner-Winkler, G. (2015). Personal identity: Philosophical aspects. In J. D. Wright (Ed.), *International encyclopedia of the social & behavioral sciences* (2nd ed., pp. 742–749). Oxford, UK: Elsevier.

Olson, E. T. (2003). An argument for animalism. In R. Martin & J. Barresi (Eds.), *Personal identity* (pp. 318–334). Malden, MA: Blackwell.

Parfit, D. (1984). *Reasons and persons*. Oxford, UK: Oxford University Press.

Putnam, H. (1975). The meaning of 'meaning'. In H. Putnam (Ed.), *Mind, language and reality: Philosophical papers* (Vol. 2, pp. 215–271). Cambridge, UK: Cambridge University Press.

R Core Team. (2015). R: A language and environment for statistical computing [Computer software manual]. Vienna, Austria. Retrieved from http://www.R-project.org/

Sagi, E., & Rips, L. J. (2014). Identity, causality, and pronoun ambiguity. *Topics in Cognitive Science*, *6*(4), 663–680. doi: 10.1111/tops.12105

Shoemaker, D. (2014). Personal identity and ethics. In E. N. Zalta (Ed.), *The Stanford encyclopedia of philosophy* (Spring 2014 ed.). http://plato.stanford.edu/archives/spr2014/entries/identity-ethics/.

Shoemaker, D. W. (2007). Personal identity and practical concerns. *Mind*, *116*(462), 317–357. doi: 10.1093/mind/fzm317

Strohminger, N., & Nichols, S. (2014). The essential moral self. *Cognition*, *131*(1), 159–171. doi: 10.1016/j.cognition.2013.12.005

Strohminger, N., & Nichols, S. (2015). Neurodegeneration and identity. *Psychological Science*, *26*(9), 1469–1479. doi: 10.1177/0956797615592381

Van Zandt, T. (2003). PSTricks: Postscript macros for generic tex: User's guide [Computer software manual]. Retrieved from http://www.dante.de/CTAN/graphics/pstricks/base/doc/pstricks-doc.pdf

Wagner, L., & Carey, S. (2003). Individuation of objects and events: A developmental study. *Cognition*, *90*(2), 163–191. doi: 10.1016/s0010-0277(03)00143-4

Williams, B. (1970). The self and the future. *The Philosophical Review*, *79*(2), 161–180. doi: 10.2307/2183946
