# Supplementary material for: Putting your money where your self is: Connecting dimensions of closeness and theories of personal identity
Source: PLoS One. 2020 Feb 12;15(2):e0228271. doi: 10.1371/journal.pone.0228271 (PMC7015397; doi:10.1371/journal.pone.0228271)
Supplement: Attachment — Submitted filename: puttingYourMoneyResponseLetterR1corrected.pdf [file pone.0228271.s002.pdf]

RESPONSE TO EDITOR & REVIEWERS
Manuscript # PONE-D-19-26910, PLOS ONE

Dear Editor, dear Reviewers,

We would like to thank you for your work on our manuscript. We have carefully considered and reflected upon each of your remaining concerns and responded to them in this revised version. We explain in this letter how and where we have done so.

Best regards,
The authors

Contents

1 Editorial Comments 3

2 Reviewer 1's comments 3

3 Reviewer 2's comments 14

4 Reviewer 3's comments 17

5 Reviewer 4's comments 21

References 26

6 Appendix A: New discussion section 30

1 Editorial Comments

E1: I have now collected four reviews from four experts in the field. The reviewers like the paper but suggest several improvements before publication. Therefore, I would like to invite you to revise your work following the reviewers' comments. I am looking forward for the revision.

Response: Thank you for giving us a chance to revise our manuscript. We are grateful for the four reviewers' careful reading of the paper, and we will address each of their expressed concerns below.

E2: Prompt a) If there are ethical or legal restrictions on sharing a de-identified data set, please explain them in detail (e.g., data contain potentially sensitive information, data are owned by a third-party organization, etc.) and who has imposed them (e.g., an ethics committee). Please also provide contact information for a data access committee, ethics committee, or other institutional body to which data requests may be sent.

)

Response: Demographic data for Study 1 was suppressed for the freely accessible dataset in the Dataverse (see below). The reason for not sharing this part of the dataset is a difference in consent forms between Study 1 and Study 2. Study 2's form explcitily addressed the sharing of datasets via repositories, Study 1 did not. Thus, a restricted dataset was added to the dataset with demographic information, available upon request by researchers at academic institutions. Note that the demographics did not feature in the analyses in the paper.

E3: Prompt b) If there are no restrictions, please upload the minimal anonymized data set necessary to replicate your study findings as either Supporting Information files or to a stable, public repository and provide us with the relevant URLs, DOIs, or accession numbers. For a list of acceptable repositories, please see `http://journals.plos.org/plosone/s/dat`[illegible]

Response: Datasets for both studies were uploaded to the Harvard Dataverse (see Woike, 2019), accsessible at `https://doi.org/10.7910/DVN/AN1QF3`. Further explanations are given in the respons to R2.5, below.

2 Reviewer 1's comments

R1.0: This manuscript presents two experiments (with American sample from MTurk) that provide a welcome addition to the psychological literature on folk judgments of personal identity. It is also an important contribution to the broader theoretical discussion that is relevant to philosophers as well. Overall, Im quite sympathetic towards the main goals of the studies and see some value in the methodological approach that has been taken (though with some issues), in particular, an attempt to apply the so-called fission cases to explore the patterns of folk personal identity judgments. However, the paper could use some conceptual polishing and a little more precision in the framing, but by and large, this study merits eventual publication.

Response: Thank you for your positive assessment and your careful reading of the text. We address each of your comments below and add some explanations to clarify methodological choices.

R1.1.1: A more general remark would be that the paper is rather dense and packed with a lot of (and less relevant) discussions that could be side-passed. The main claims could be presented in a more condensed form and more tied to the evidence produced. I understand that there are many strands of research and theorizing in this area, but not all of it is equally relevant to the claims of the current paper. The paper is massive and some tightening up would be desirable (especially introduction and general discussion sections).

Response: We understand your concern in pointing out that the paper is relatively long. Part of our contribution is aimed at illuminating several strands of theorizing that might not be familiar to psychologists who have used "personal identity" as a very different concept. Our theoretical contribution that goes beyond the empirical contribution in sorting out different uses of this concept in psychology and philosophy requires some breadth to be viable. Readers with different backgrounds will, as evidenced by the selection of reviews compiled in this letter, different parts of the explanations more and less helpful. The theoretical contribution goes beyond the claims in the empirical part, and we have added a new section to the discussion to spell out this gap and give some ideas of this gap could be bridged by future research. As the theoretical contribution follows from our interpretation of the relevant literature, we found it important to cover this field in the chosen breadth. At the same time, the non-conventional format of PLOS ONE affords the opportunity to exceed the page restrictions of conventional print publications, which was one important reason for our choice of the journal (see also R2.1 for a positive assessment of our introduction).

R1.1.2: Following on this general remark, there is one interesting feature to this paper. I am not sure what to make of it. That is, the authors throughout all the paper (and especially in the general discussions section) devote a considerable space to discuss possible objections and possible responses to those objections. For instance, in the

general discussion section there is a whole rather long sub-section ("The fission scenario as scientific fiction: Benefits and validity concerns") devoted to discuss the validity of the scenarios. The fact that the authors themselves took great pains to identify possible objections to their methodology is a great and welcome endeavor. But it does also show that there are some inherent problems in the employed methodology and, I must say, not all attempts to respond to objections worked very well. I guess a more honest strategy would be simply to acknowledge the problems and vow (so to say) to deal with them in the future studies. Actually, while reading the paper similar objections where popping up in my mind, and particularly in respect to methodology. Bellow Ill raise some more concrete questions and concerns that could be addressed in the current paper or in the future studies.

Response: We can give a simple explanation for the space taken up by discussions of possible objections. The paper underwent two major revisions at a different journal, and many of the points were raised by one of the reviewers, who was ultimately satisfied with our responses to their concern (the resulting length of the paper exceeded the requirements of that other journal, though). Thus, not all of these points were raised by ourselves, and we would not have raised some of them unprompted and, as evident by our responses, do not agree with all of them. It did still not seem appropriate and honest to simply eliminate this discussion from the text. No methodology is without problems, but if there is a chance to go beyond a generic limitation section, we do not see a reason not to engage with these counter-arguments. Many of the early concerns were addressed in Study 2 that had not been part of the earliest version of the manuscript. It would not be honest to promise future studies when these are not actually planned. There are different ways in which we would like to extend our analysis of the central topic, but these extensions go beyond the scope of the present manuscript. We present a novel paradigm, thus we cannot rely on the power of convention to suppress criticism. At the same time, of course, a single manuscript has a limited capacity to fully engage with every single creative suggestion of possible variations of the basic structure, especially when a single test involves accumulated days of participant engagement. What the manuscript offers, nonetheless, is that we can provide converging evidence within and between studies.

R1.2: Closest continuer theory and identity?
It is not clear how the proposed framework is different from (or similar to) Rips at el. (2006) proposal that also builds on Nizicks theory. Some clarification would be useful. However, it should be mentioned that Rips at el. emphasized causality in their approach: "the continuer of the original object must be a causal outgrowth of that original" (Rips, Blok, & Newman 2006: 7). That is, perceived causal continuity is deemed to be central in producing and maintaining and an individual object over time and transformations. Correct me if I am wrong, but causality was not mentioned in the current formulation of the Closest continuer theory. Closeness metric, I assume, here is used to flesh out the theory. At any rate, if perceived causal continuity is deemed to be central for identity judgments, then this is a very general theory of rather thin

individual identity (and not of rather thick personal identity). In other words, if my characterization is correct, what the proposed version of Closest continuer theory adds to the discussion about personal identity per se?

Response: Rips, Blok, and Newman (2006) indeed based their theoretical approach on Nozick (1981). This is not too surprising, as Nozick's text was one of the major contributions to the field in philosophy. There is a major distinction between their approach and ours as Rips et al. (2006) focus on the question of object identity, not personal identity. It is part of our theoretical argument that this distinction does not matter as much for the concept of "identity" as some psychologists would believe, but there are most certainly unique aspects in personal identity. Thus, we focus in our design of the empirical approach both on the history of personal identity in psychology (James, 1890) and the important contribution of Parfit (1984) in "Reasons and Persons".
We are not completely certain, in which sense you use "thick personal identity" and "thin individual identity". There are no degrees of thickness in the concept of identity that we inherit from the philosophical debate. Causal continuity falls in the domain of criteria for assessing identity between objects (and in some thought experiments, also between persons; Campbell, 2005; Kolak & Martin, 1987). As both continuers undergo a similar process, we cannot address the question of causal continuity apart from underlining that the accident was deemed compatible with continued existence by many participants. We focus on a set of criteria that originated in the philosophical literature (see also the following response). The role of causality potentially did not receive sufficient attention in the previous version of thje manuscript. Thus, we added a new paragraph to the discussion:

> Critics of our scenario might further object that our random collage of features in the two continuers destroys the causal connection between past and present states necessary for identity (Matthews, 2000; Parfit, 1984). Preschool children already individuate objects and persons spatio-temporally (Gutheil, Gelman, Klein, Michos, & Kelaita, 2008; Wagner & Carey, 2003) and, following Sagi and Rips (2014), causal histories receive special attention in linguistic disambiguation in discourse. In all our scenarios (except the two extreme cases with exact duplicates), change in characteristics was induced by an accident, an unusual life event that disrupts spatio-temporal continuity. This fact might strengthen impressions that identity is not preserved. According to data reported by Nunner-Winkler (2015), for example, participants regarded changes in attitudes or beliefs that were due to normal life experiences as non-consequential for identity judgments—as opposed to changes induced by brainwashing, severe medical conditions, or accidents. Therefore, the nature of the transformation might play a role in our participants' judgments. Note that both continuers underwent the same procedure, so this factor cannot explain differential assessments. Although the abruptness and symmetry of the original person's transformation prevents the application of spatio-temporal

continuation criteria, participants might still construct "fictive causal histories" (Fields, 2012) to assess which of the two continuers might have the better chance of being the result of changes within an ordinary life.

R1.3: What "person" consists of? Another questions is even more pressing and has some consequences for the methodology used. The authors differentiated five dimensions of the person and assumed that these five categories are the way how the folk conceptualize personhood. With all fairness, the authors did provide justifications for the choice of these dimensions, but all of it relies on psychological research and theories. What is more important in this context, I would argue, is how folk themselves conceptualize the personhood. So my question is, did the authors try to do pre-testing or surveys in order to extract the most salient dimensions from the people? On the other hand, I am not sure whether it is warranted to have two psychology-related categories as separate dimensions: personality and psychology / memory and knowledge. This is clearly one category, but with some sub-categories. Besides, results indicate that both subcategories are the most significant dimensions in allocating insurance money. Also, in judgments of importance of these dimensions, both sub-categories correlate pretty well (eg., Table S5). Moreover, the choice not to include moral attributes is likewise not well justified (like Strohminger and Nichols did) They write: "we did not differentiate between personality and psychology on the one hand and moral values on the other. In separate evaluations, our participants ranked memories and psychology as more important for identity than moral values." (p. 32). This post hoc justification doesn't really help. At the outset, in determining dimensions of the personhood, it doesnt matter how participants ranked an importance of psychology and moral values for identity. First, what matters is whether participants see it as a separate category that makes up the person. Besides, it looks quite strange when the authors distinguish possessions as a separate dimension (that, apparently, had no effect whatsoever), but excluded moral attributes. Finally, it is not clear why for the category of "social relations" there was only "friends". It is really good that the study had this dimension, but the scope of social relations has been rather limited. This doesnt capture the whole complexity of social relations. Still, with all fairness, the authors did address this issues in Study 2, but again the choice of the items was not clear and transparent. Why this particular list of attributes?

Response: We would like to respond to the distinct parts in this critique in sequence. We will start with (a) the contrast between academic and folk theories, discuss (b) the reason for separating memory and psychology, (c) the reason for not including morality and its "post-hoc justification", (d) problems with the category of "social relations", and (e) concerns about the particular set of chosen features.
(a) You object regarding our choice of dimensions that "all of it relies on psychological research and theories" as opposed to folk theories. At an early stage, we discarded the idea of studying folk theories of personal identity without giving any guidance and focus, as we would have likely missed our target. It is highly unlikely that laypeople would be

able to give an explicit substantive account of "personal identity" as a concept, given the observation that most contemporary psychological texts use the concept in ways that are inconsistent with the philosophical concept (as discussed in the discussion). In other words, we did not want to elicit a definition of "person", but decided to confront participants with a puzzle case that required judgments integral to their implicit theory of personal identity. This is similar to eliciting the understanding of physical invariance by posing problem cases instead of asking children for explicit physical theories (that can be formulated much later than the implicit understanding can be observed).

(b) The psychological criterion and the memory criterion are distinguished in the philosophical debate. Of course, memory is often considered a part of psychology, but memory content, on the other hand, depends much more on relations between persons and other facts. I can imagine having a specific memory replaced (I was in Athens, not in Barcelona, for example) without being able to derive changes in predictions about future behavior.

(c) Moral traits, in contrast to the features we included in the design, has not been focused on in the historical literature. We would not have raised the point about including it without the publication of Strohminger and Nichols (2014), which, incidentally, happened after the data collection for Study 1 was concluded. This might explain the post-hoc character of the argument. There are several reasons why we would still not be entirely convinced that an addition would be warranted: The use of personal identity in Strohminger and Nichols (2014) is not compatible with our understanding of the term, as explained in the subsections "The crisis of identity in the social sciences" and "Separating closeness from identity". Reference to the philosophical concept is relegated to a footnote that does not do justice to the quoted texts. Morality, as a distinct criterion has not featured centrally in the philosophical literature on identity. There are few references to earlier phlilosophical articles. Indeed, two references to establish philosophical interest are cited "in press", one of the being Prinz and Nichols (2016). But Prinz and Nichols (2016) is not a conceptual paper, but a similar study in experimental philosophy, asking the same question about wehter someone "is the same person as before the accident" on a six point Likert scale, analysing change via one-sample t-tests against the scale midpoint with no information about variance. At least some of the scenarios involved does not necessarily present evidence for the general superiority of a morality criterion over a memory criterion, as changes in morality are accompanied by severe changes in behavior towards others in contrast to changes induced by memories in these scenarios. In other cases, memory changes cause only errors of omission, not errors of commission which can have much more sevee implications for behavior. Further, the separation between personality and moral traits does not seem to be as clear-cut on the operational level: Conscientiousness, one of the "big five" personality traits is featured as a moral trait in Strohminger and Nichols (2014) and contrasted with personality traits, just to name one of these problems. We would agree that further analysis is warranted, but would also expect a substantial amount of conceptual quicksand to be mitigated to determine the proper place of morality. Adding this complex concern to our study would more than likely increase its length substantially. On the other hand, we also agree that we limited our discussion of morality in the discussion, so that we decided to extend the

brief explanation and also give more weight to previous research investigating morality[1]:

> [...] in contrast to Strohminger and Nichols (2014), we did not differentiate between personality and psychology, on the one hand, and moral values, on the other. Evidence from several studies considering real-world personal transformations has indicated that identity judgments are most heavily influenced by changes or non-changes in moral values (Strohminger & Nichols, 2014). Changes in morality were judged to be more relevant than changes in (nonmoral) personality attributes or memory. In a similar vein, Strohminger and Nichols (2015) found that changes in morality in patients with neurodegenerative diseases strongly determined changes in perceived identity. Nunner-Winkler (2015) reported on a study asking participants which changes would lead them to see themselves as a different person. Ideas about right and wrong and sex membership were considered to be quite important; appearance and money were considered less relevant (although some participants rated looks to be important, consistent with our distributional results).
>
> The distinction between moral and nonmoral traits is somewhat ambiguous (e.g., conscientiousness was considered as a moral trait rather than a personality factor in Strohminger and Nichols (2014)). One person's morals do not and cannot exist in a social vacuum, moral consensus is central for co-ordination, affiliation and conflict resolution. Morality stands in complex relations to beliefs, values, behaviors and communities. It also depends on memory in nontrivial ways. Some of the induced changes in the scenarios even involved the loss of the moral faculty with a likely ripple effect reaching other dimensions of the self. If this perspective is true, the relevance of morality for personal identity might lie in these possibly disruptive consequences of changing one's morals in relation to one's environment and not because of its self-defining importance. Evidence for this interpretation is found in two studies demonstrating that changes in widely shared (and therefore less unique to the individual) moral values are considered to lead to more changes to the person than changes in controversial moral beliefs (Heiphetz, Strohminger, & Young, 2017; Heiphetz, Strohminger, Gelman, & Young, 2018). For controversial moral beliefs, that might be considered most defining and informative for describing a person's self, the effect was weaker than for memory. Also, the changes in memory induced by our scenarios would induce both errors of omission and errors of commission, which can have differential impacts on moral behavior (Stevens, Woike, Schooler, Lindner, & Pachur, 2018). In contrast, some studies focus mostly on omission errors due to memory changes (e.g., Prinz & Nichols, 2016), which are describes as having more limited effects on behavior towards others than changes in morality. In our scenarios, dimensions are replaced by random sampling from the participant's reference population, which is a different operationalization of change. Heiphetz et al. (2018) showed, for

[1] Note that in-text citations appear different from those in the revised manuscript, as we use an author-year citation style in this response letter.

example, how the perceived change was mediated by perceived disruptions of friendships.

Some argue that morality is not even conceivable *without* personal identity (Mills, 1993; Parfit, 1984; D. W. Shoemaker, 2007). Most people also seem to have inflated beliefs of their own morality (Newman, De Freitas, & Knobe, 2015). In separate evaluations, our participants ranked memories and psychology to be more important for identity than moral values. Nonetheless, a further decomposition of the broad headings we used in our study would be feasible and interesting in future research. In particular, the role of moral traits and behavioral tendencies could be considered separately, even within a similar factorial setup as the one we employed.

(d) We agree that our "friends" category does not do justice to the full range of social relations. There are some obvious complications with many other solutions, though: A replacement of family would most certainly involve a complete change of genetic constitution and directly impact other categories, for example. Friends are to some degree self-chosen and have a central place in one's social circles. The choice of items in Study 2 was based on a list of suggestions from a previous reviewer who did not offer reasons for this list. We found its elements to be representative of distinct social domains and sets of relationships, thus we included it in Study 2. Taking up your comemnt, we added the following paragraph to the discussion:

> The social dimension could be further differentiated, as well. Parents were considered to be more important than friends in Study 2. Of course, parents influence a person directly through the transmission of genes as well as indirectly through instruction and parenting behavior; changing one's parents cannot be considered a merely social manipulation and could well have an impact on every other dimension.

(e) We would like to re-emphasize the correspondence of our features to those focused on in the extensive definition of self by William James cited in the beginning of our article. It is true that we split "psychic powers" into two components, but please note James's central concern for possessions. We would also like to emphasize that the very structure of our factorial design does not necessitate completeness of the list of attributes to allow for comparisons between specific dimensions (a design with body and psychology alone would still allow for addressing part of our questions. Specifically, our design allows for a neutral response, we would not expect spurious weights placed on dimensions even if all of them were considered to be irrelevant.

R1.4: Experimental scenarios.
Authors spent some time in justifying their choice of sci-fi scenarios. However, I share similar worries with critics who view such scenarios, either in philosophical thought experiments or in psychological experiments, with suspicion. I must say that the sci-fi type of scenario is rather demanding on our imaginative capacities, there are way

too many counterfactual elements that a participant has to keep in mind while "consulting" his/her intuitions on identity. Specifically, scenario starts with "hyperspace": "You enter hyperspace to travel large distances and leave it at your new destination." Then you are asked to imagine: "For a brief moment, the present universe overlaps with a parallel universe." Finally, last section introduces:"Your travel agency contacts you while you are still in hyperspace and informs you that due to the overlap it has been calculated that, unfortunately, not one but two people will leave the hyperspace at your target destination: person A and person B, while you will no longer exist in your present state." I know people who dont like sci-fi movies and admit that don't understand them. So careful, unambiguous, construction of scenarios that is valid across different groups of people would be important. Did the authors make some comprehension pre-test (or some sort of cognitive interviewing)? If not, this is something for future research to consider. Notwithstanding comprehensibility issues, I worry that the description of the scenario that started from 1st person perspective and ended with 3rd person descriptions of A and B persons might bias participants in some way. That is, it could have been the case that at least some participants looked at A and B persons as different from oneself since they were described from 3rd person perspective (this could also partially explain the predominant equal split, as if it was a split of money between, say, your kids, but not you in two different incarnations). Of course, its just a hunch, some other variants of the scenario need to be tested in order to rule out this possibility.
At any rate, authors claim that: "intuitions derived from cognitive processes during these unreal and outlandish examples may not be necessarily meaningful when measured against the constraints of reality but rather cast light on how we use and reason with the concept of identity." (32). It might be the case. But a worry remains: does it really reveal any systematic and deeply-held intuitions/beliefs about personal identity or only post-hoc responses to rather weird scenarios. I might be wrong, but again the results indicated fair split of the money between A and B persons, which again points to some problems with scenarios. So, pre-testing is crucial here.

Response: Again, we will unpack our response to these points. We start by (a) commenting on the use of our sci-fi scenario (also addressing your last point about weird scenarios) (b) commenting on the role of personal perspectives.
(a) Compared to standard paradigms employed in decision making research or cognitive psychology, the presented scenario might demand some suspension of disbelief, but it is far less cognitively demanding than other paradigms routinely employed in these disciplines. It is the afore-mentioned power of convention that would make a choice between the gambles

A $(200, 0.23; 350, 0.41; 500, 0.35; 1000, 0.01)$ and
B $(220, 0.21; 320, 0.38; 520, 0.38; 900, 0.03)$

appear more defensible as a paradigm for use on MTurk than a narrative scenario that poses a conceptual puzzle. Based on responses, comments and results, we feel confi-

dent that the use of the scenario with our chosen sample was appropriate (and based on comprehension checks in previous studies, we would doubt that the gamble-problem would lead to less confusion when elicited). Certainly, some of the responses indicate idiosyncratic reactions to the task, but due to the factorial structure it is unlikely that these idiosyncrasies were able to influence our findings (and note that we ARE able to identify them in our design). The first author regularly employs comprehension checks in MTurk studies and found comprehension failure rates of more than 50% for some "standard" economic games. Instead, many participants welcomed the novelty of the scenario and took care in phrasing their responses. Comparisons between MTurk and student samples have been mostly favorable in terms of data quality and attention. The main part of the scenario was pre-tested in a smaller pilot study together with other variants of puzzle cases found in the literature, and ultimately chosen for its potential to generate meaningful continuous responses.
(b) Your worry about personal perspective in thought experiments is well-founded. Specifically, our scenario is related to Williams (1970), who focuses on the relevance of the personal framing on intuitions. It should be noted, though, that the indicated inconsistency in pronouns is preferable to alternatives, which could be considered much more leading. It is therefore a different question, whether variations in pronouns would make us expect pronounced differences in responses. Nichols and Bruno (2010) found no significant differences regarding first-person and third-person perspective when directly testing and comparing versions of the original scenario. Note that in a subsequent study (in preparation) featuring a different version of the transporter accident that is described entirely from a third-person perspective, we observe similar responses concerning the relationship between original and continuers. Based on this finding, a variation of personal perspectives should not be expected to change results. As we believe that your concern might be shared by other readers, we added a new paragraph to the discussion section discussing the potential for such influence.

> Participants responded to our scenario only from a first-person perspective. First-person evaluations of identity and survival might differ from third-person evaluations (Perry, 1972; S. Shoemaker, 1994). For one thing, many legal, practical, and social concerns can be fulfilled by a person who is a spontaneous true copy of the original person. How much participants care about the disruption of continuity might therefore differ depending on whether the replaced person is *them* or a neutral other person (Matthews, 2000). Rorty (1973) distinguished between an external observer's perspective on individual identification and an individual's internal perspective; features essential to an individual's self-perspective might be irrelevant for an observer, and not all philosophers assume that first-person judgments have final authority (S. Shoemaker, 1990; Sider, 2001). On the other hand, at least one study explicitly testing for the effect of perspective on intuitions about identity based on Williams (1970) found no substantial differences between a first-person and a third-person perspective (Nichols & Bruno, 2010). At the same time, continuers were introduced from a third-person perspective. This shift was

necessary to avoid a pre-judgment of the question how the original person relates to them, but might create a perceived distance. This could make it easier to give a negative answer to the survival question, but would have a symmetric influence on identity responses for both continuers.

It is less clear which perspective is better suited to evaluate claims about identity and survival. We assumed that involving the participant would be the best way to increase attention to the situation and diligence in responding. We induced a connection between our categories and their real-world instances, as perceived by the participants. A participant evaluating the importance of "body" in the money allocation problem will thus take the perception of her own body into consideration, which may lead to different results than when considering the importance of *a* body. This fact might play into our finding that a subgroup of participants placed a negative value on the continuity of the body.

R1.5: On the same note, there are some potential problems with post-questionnaire items:
1.1.5. Q9Q13 Importance for identity. How important are the following aspects for you when it comes to determining identity between two people? This is an ambiguous question. One can read it as a question about qualitative identity between two numerically different people. As a result, some participants might have responded to this reading of the question.
1.1.6. Q14Q18Importance for survival. How important are the following aspects for you when it comes to determining the survival of a person? This is an ambiguous question as well. The notion of survival here is used in a more philosophical sense, but this is also an everyday term that has other semantic connotations. For instance, on could read it as a question about "what helps someone to stay alive" or "what helps someone to endure". Thus, it is important to disambiguate between different readings. One can not assume that non-philosophers, ordinary folks, share similar understanding of the philosophical technical terms.

Response: (a) Regarding the identity question: Based on our theoretical model, identity judgments involve assessments of closeness. In phrasing the question, we chose to accentuate that we understand the term "identity" as relational, not substantive. Given the present discourse on identity in psychology, the substantive interpretation would have been likely by omitting the part "between two people". For the closeness metric, the two possible interpretations of the question should lead to similar answer It is highly unlikely that participants interpreted the question as referring to strict qualitative identity: In this case, all responses would have been required to pick the maximum value, as qualitative identity implies the complete lack of differences. The found pattern is evidence, instead, that participants judged weighted similarity, as implied by the model. This also corresponds with the found convergence in results.
(b) Regarding "survival", you argue that we should have provided more rather than

less guidance. We should note that our interest in interpretations of survival was secondary to our interest in identity. In this case, we were actually interested in finding out whether there actually *are* differences between philosophical and folk usage of the term. Most importantly, we wanted to know whether criteria for assessing survival were different from those for assessing identity, as stated in the relevant Research Question. The philosophical, technical use is likewise not unproblematic or unambiguous (e.g., compare Brueckner, 1993; Campbell, 2005; Johnson, 1997; Johansson, 2010; Matthews, 2000; Moyer, 2008; Parfit, 1984; Rachels & Alter, 2005).

R1.6: These potential problems in the methodology makes it hard to interpret results. However, I still belief that it is possible to extract some interesting and valuable information from the results as they are. While keeping the aforementioned concerns in mind (and presumably for future research), current results are consistent with previous work indicating that psychology is the most salient criterion in determining personal continuity, even in the case of fission. This is something akin to what Parfit advocated for – psychological connectedness. After some additional theoretical pruning, conceptual polishing and honest acknowledgment of some methodological issues (vowing to address them in the future), a revised version of the paper can be accepted for publication.

Response: Thank you again for your generally positive assessment and your helpful suggestions to improve the manuscript.

3 Reviewer 2's comments

R2.1: This paper investigates people's beliefs about continuity of a person, empirically assessing the adequacy of Nozicks closest continuer model and testing which bases seem to most inform continuity judgments.

I'll be fairly brief because I think this paper is quite clear-cut. My understanding of the primary criteria for PLOS ONE is internal validity are the conclusions well-supported by the data. I think the studies are well-designed, and the analysis is comprehensive and the conclusions are carefully drawn and accurately capture the results.

Ill also add that I think the research question is of broad inter-disciplinary interest, and the paper does an excellent job of surveying that broad and inter-disciplinary literature, and the questions being asked have been debated but not answered in the prior literature. So, I think the contribution is quite clear.

Response: Thank you for this assessment regarding the paper, its contribution and your evaluation of its potential audience.

R2.2: There are some limitations of the paper. I think the primary limitation is that the paper uses highly unrealistic "science fiction" scenarios. I agree with the authors reasoning for doing this, and the paper includes an entire section at the end with a fair discussion of the benefits and limitations of this approach.

Response: Thank you. We agree that it is necessary to respond to these concerns (and also reflect them in delineating potential limitations). For a related point, see also our response to R1.4 above.

R2.3: I was also a bit concerned about the characterization of the monetary allocation task as measuring only (or primarily) beliefs about continuity of identity. The discussion on p. 8-9 emphasizes the reasons why this measure should be responsive to beliefs about identity, but underplays a bit the possibility that other factors might influence the allocation decision. Given that dictator games typically reveal substantial sharing with distinct other people without a direct incentive to do so (e.g., as summarized in Engel 2011, which is cited), clearly other motivates can impact allocation decisions as well. The GD is more cautious on this point, and the other measures which do not have this issue reveal similar results, so I think this is a minor concern, but perhaps the initial discussion could be a bit clearer on this point.

Response: We have added to the discussion in question to clarify the possibility of other factors influencing the decisions. One reason why we did not conceive of this threat as too serious is that any factor unrelated to the tested dimensions would not be expected to change the relative weighting of dimensions. Rather, the expectation would be an attenuation of the effect via the addition of unsystematic noise. This is due to the completely symmetric setup of all factorial conditions (each feature combination appears again with reversed sides). Any tendency towards equal splitting does not change the expressed order of importance (only for an exact equal split the participant would appear neutral). Any other-regarding consideration that would change our findings should move responses towards an equal split (complete selfishness would lead to responses in accordance with identification). We added the sentences:

> In our design, all possible composition patterns were presented twice, with the continuers assigned to the two positions switched in the second version. Thus, any effect due to fairness consideration would move monetary allocations towards an equal split, effects unrelated to the factorial distribution should stochastically even out. Responses influenced by these two factors could thus attenuate discovered relationships, but they should not introduce spurious new relationships.

In this way, we acknowledge the possibility of changed findings due to fairness considerations, but also state to what degree our design shoudl be robust against some of their effects.

We would also agree with you in regarding the convergence of different measures within and across studies as a good indication for the robustness of our procedure against the possible noise factors, thus we can at least indirectly confirm our stated assumptions.

R2.4: Overall I think this is an important and well-done paper that will advance the debate about how people think of identity continuity.

Response: Thank your for this evaluation.

R2.5: My greatest concern, however, is that the authors do not plan to share their data. This is contrary to PLOS-ONE policy. It is the editors decision, but in my view, this is a serious issue as it relates to publication in PLOS ONE. The data in this paper is not the kind of sensitive or proprietary data that justifies an exemption, and IRB consent terms are, at least in part, a choice made by the authors. Deidentified data for minimal risk studies is commonly made public these days, and a long history in psychology has shown that "available upon reasonable request" policies simply do not work to ensure the necessary transparency. My opinion would typically be that if properly deidentified data (i.e., excluding not only identifiers but also demographics, if need be) cannot be posted, then the authors should submit the work to one of the journals that does not yet require data sharing.
However, I think this is somewhat of an atypical case. I noted the exhaustive online appendix, which is extraordinarily transparent about the data summaries and analyses. If the data absolutely cannot be posted, then I would suggest the following. First, I would like to see more detailed documentation on this point, to ensure that the data ethically cannot be shared (as opposed to the authors simply preferring not to share). Second, the online appendix should be permanently hosted on a public repository such as OSF or on PLOS ONE if published (i.e., as opposed to dropbox, where it might lapse into unavailability). Third, the full data analysis code, a data dictionary and a statement detailing the procedure for requesting the data including the timeframe for response should also be posted. I would also like the authors assurance that the data has already been prepared for sharing upon request.

Response: We very much appreciate the nuanced phrasing of your concern. Upon receiving these comments (also reinforced by the journal), we—that is the first author—immediately contacted the IRB involved (Ethics Committee of the Max Planck Instiute for Human Development, Berlin) to negotiate possible, responsible ways of sharing our data. Here, I would like to add a personal note as first author to explain the specific sensitivity: In several other manuscripts I focus on the possible conflict between open data practices and privacy concerns—especially for crowdsourced participant pools. This work has actually sensitized me to the merits of both sides of this coin, so I lean towards advocating responsible data sharing (alas, with more restrictions than is common practice). One central feature in my recommendations is the importance of consent, thus I

feel obliged to follow through with these recommendations in my own work. At the same time, I would not advocate for unreasonable barriers to sharing particularly, when data sets are truly deidentified and their content can neither be constructed as personally sensitive nor as containing quasi-identifiers for merging attacks. Thus, we have revisited the consent forms employed in both studies.
As a result of this process, Study 2 was considered to be unproblematic for sharing: Participants actually agreed to the possibility of their data being uploaded to public repositories. To avoid reidentification, I took a number of precautions: The age variable was grouped into categories, open-format answers were not included in the data set, time stamps and identifiers were removed. Given the remaining information, a participant would be hard-pressed to identify their own data row, which I find acceptable for sharing. I do not want to judge whether participants find the data sensitive or not. Some participants might argue that their attitude towards body, friends and possessions qualify as such. I uploaded the dataset into a published Dataverse in the Harvard Dataverse
The consent form for Study 1 did not make the explicit suggestion that public repositories could be used in the future. At the same time, we diverged form the standard institutional form (which of course was evaluated by the IRB) in not negating this possibility. Thus, the IRB did not advise against publishing the data for Study 1 in a restricted form. I decided to add two versions of the dataset to the Dataverse: One with age brackets and gender included, the other without it. The full dataset was protected by making it requestable via the platform, whereas the limited dataset is freely accessible. Given the lack of the general consent for publication, I apply the criterion that it should not be possible for a participant to identify their own data based on quasi-identifiers (having perfect memory of given responses would allow for reidentification of their own data, but perturbation would not mitigate any foreseeable risk, here).
You can access both datasets at `https://doi.org/10.7910/DVN/AN1QF3`.

We have changed the Data Availability statement to:

> The deidentified data for Study 1 and Study 2 are published as (Woike, 2019) in the Harvard Dataverse and are accessible without restrictions. Note that the dataset for Study 1 is published without age and gender information (not analyzed in this manuscript). The Dataverse also offers a restricted version of this dataset (with age and gender information) that can be made available via the platform to researchers at academic institutions.

4 Reviewer 3's comments

R3.1: This study on reactions to hypothetical cases of fission is a novel and important contribution to the literature on lay concepts of personal identity and survival. It presents important new results on empirically severely underexplored but theoretically highly significant branching thought experiments (especially notably, the study

demonstrates that many participants identified the original with both continuers of the fission accident simultaneously); introduces methodological innovations; and enriches the empirical debate with important theoretical perspectives (namely, those by James and Nozick). I am confident that it should be published.

Response: Thank you very much for this assessment.

R3.2: First, another body of literature in which structurally similar cases were explored in order to shed light on folk thinking about personal identity can be found in cognitive science of religion. Most notably, I would like to draw attention to work by Claire White on reincarnation beliefs (the most relevant paper is White, C. (2015). Establishing personal identity in reincarnation: Minds and bodies reconsidered. Journal of Cognition and Culture, 15(3-4), 402-429.). The experimental task in these studies is to identify the "true reincarnate" from a set of potential candidates that vary in their features. These studies suggest a potential alternative explanation of present results - that it is not the type of content (e.g. presence of autobiographic memories vs presence of body) that drives reidentification judgments but differences in distinctiveness of ascribed features (and the more distinctive are the preserved features, the less likely it is that their presence is just a pure coincidence, the more attractive becomes an explanation of occurrence of these distinctive features in terms of preserved identity). It seems that some of the results of the present study could potentially be explained in this way (see e.g. Fig 7, where generic features, such as nationality, profession or group membership, seem to gravitate toward the less important end of scale). It would be great if the authors briefly engaged with this potential alternative explanation.

Response: This is a very interesting switch in the design of a reidentification experiment. Our experiment eliminates uncertainty about the origin of component parts in the successors. Likewise, as we note in the discussion, many of the paradigms used in psychology are focused on a single successor and leave no doubt about its status. The mentioned paradigm allows for the intriguing situation that uncertainty could created with a single successor. This paradigm requires a different suspension of disbelief, affecting those who do not share beliefs about reincarnation (80% of US Americans, according to the study), but does not necessarily involve a more extreme stretch of the imagination. In this respect, the paradigm seems an attractive alternative. In fact, Strohminger and Nichols (2014) use a variation of the reincarnation scenario to differentiate superficial traits from fundamental traits in their Study 4.
There are also some downsides to these scenario versions: Participants have to subscribe to some theory of how reincarnation works, or at least determine which aspects of a human being are preserved. Reincarnation into the same physical body is less frequently found in religious beliefs than reincarnation into different bodies with varying understandings of what comprises the non-material being (what is put back "into flesh").
C. White (2015) use a scenario, in which they ask for picking out the true reincarnation of a deceased among several candidates or ask for likelihood ratings that a living

person is a reincarnation. What both questions assume, is that the deceased person *can* be reidentified as one living person. The focus will thus be on distinguishing features, and the focus on distinct features might be partially due to this underlying assumption. Gricean norms would further lead participants to assume that the reoccurrence of matching distinct descriptions will be non-accidental, even if they would not necessarily agree that the mere continuity of the distinct feature alone would be sufficient for establishing identity without this context.

In the description of their reincarnation variant, Strohminger and Nichols (2014) unfortunately use the somewhat opaque terms "real identity" and "true self", and it is again not entirely clear, how these terms relate to personal identity. In addition, their scenario places a focus on religion: "Participants were told that many religious traditions hold that humans can be reincarnated after death into a new human body" (p. 166). Thus, it is possible that moral terms are chosen for compatibility with religious morals (the most frequently preferred terms were "honest" and "trustworthy").

We have added a reference to these scenario types to the newly added subsection that is contained in the Appendix below.

R3.3: Second, the authors write on p. 31 that 'participants did not seem to differentiate between criteria for identity and criteria of survival'. While this is true for explicit weighting of dimensions, a quick glance at clusters in figures S13 and S16 suggests that for quite sizeable proportion of participants ascriptions of sameness and ascriptions of survival diverged markedly. Notably, in clusters A and D (approx. 40% of participants in Study 1) and A2 in Study 2, participants ascribed survival and denied sameness. The same trend, while not crossing the midpoint (or crossing it less markedly) can be seen in several other clusters and also in mean agreements. It would be great to have additional stress (and additional comments on) the relationship between identity and survival (not only on explicit weighting of criteria but also on ascriptions of identity and survival themselves, where people seem to often differentiate between the two).

Response: Thank you for alerting us to this over-generalization. We have modified the sentence following the sentence you cite to stress that this result is true for average responses: "Their average ratings of the importance of the various dimensions were similar for both." It is also true that there are some specific patterns for clusters that weight specific dimensions as having differential importance for identity and survival. In most of these cases, the relevance for identity is regarded to be slightly higher than the relevance for survival. To some degree, survival does seem to constitute a lower bar than identity, from the view point of these participants. Thus, I could survive an accident, for example, but change to a degree that would make me a different person. Parfit (1984) speculates about the possibility that chains of connectedness might be broken within one lifetime.

Further, you are right that some participants differ in their acceptance of the statements about survival and sameness. But this is less of a contradiction regarding the relationship between identity and survival than implied: Participants in cluster A' affirm both that

the person has survived *and* that the person is the same person (maximum of Person A or B), they have a lower rating for the statement that the person has not changed and is the same as before. Identity is perfectly compatible with change, or the old person could not be reidentified as the same person as the child. The same is true for cluster D' in Study 1 and cluster A_2' in Study 2. We explore some of the cluster patterns in the supporting material, and drill down on feature combinations that might help to explain intransitivity. Given the length of the manuscript (see the comments under R1.1), we would shy away from adding this discussion to the main manuscript, even if we share your interest in these questions.

5 Reviewer 4's comments

R4.1: The manuscript explores continuity of personal identity using Nozicks closest continuer theory. The manuscript examines a series of questions related to the theory including: What metrics determine closeness? Do peoples intuitions about continuity of identity conform to transitivity? Do identity judgments follow monetary allocation decisions? Do people follow the logic of Nozicks theory? Do people's explicit importance ratings predict monetary allocation decisions? In two studies, using a fission scenario, the manuscript explores these questions and some of the major finding are: memories/knowledge and personality/psychology tend to be the most important closeness metrics, peoples responses do not strictly adhere to transitivity of identity, judgments of identity tend to follow monetary allocations, explicit importance ratings predict monetary allocation decisions.

Response: This is a good summary of the main findings.

R4.2: The manuscript does a nice job of examining some specific instances of whether Nozick's theory describes how people think about personal identity (e.g., examining the violation of transitivity and similarity between identity judgments and monetary allocations) and clarifying what metrics are used in closeness judgments. However, because of the number of questions the manuscript aims to explore and the large number of results, it is a bit hard to follow, particularly the results section. The large number of results can make it hard to differentiate what the main findings are from what more peripheral findings are. Further, I think more clarity about what the goals of the research are and precisely what questions/results are relevant to each goal would be useful.

Response: Thank you for your positive assessment and your explanations regarding your concerns about organization. We agree that there are a large number of results reported in the study (and an even larger amount when also considering the analyses in the supplementary material. There are several reasons for this feature of the studies: First, we introduce a novel paradigm and therefore needed to show converging evidence for our findings. This required some redundancy that we actually welcomed for demonstrating the robustness of our findings against changes of measures, ordering and scenario texts. We added a reference to this goal when introducing Study 2:

> In Study 2, we replaced the continuous task with a binary choice task, in order to assess the degree to which the results of Study 1 might have been shaped by our design choices (Landy et al., 2019). All hypotheses formulated for Study 1 were also tested in Study 2, replacing references to monetary allocation by references to the new task.

Second, some of these robustness checks in Study 2 were prompted by reviewers' concerns regarding a previous version of the manuscript. As the paradigm has not been tested independently, we can neither point to previous research nor, convincingly, to contrary intuitions to alleviate such concerns.
Third, as we need to explore the suitability of this scenario to inform the discourse on personal identity, we added several exploratory analyses to estimate its potential. As a result, we ended up with a large number of separate findings that would be more customary in cognitive psychology papers than in experimental philosophy articles. Thus, we decided to move a substantial number of (interesting) findings into the supplementary material, and organized the manuscript by hypotheses and research questions. We return to the research questions when presenting the results and we implemented your suggestion (R4.8) to make these references more transparent.

R4.3: It seems that there are at least three goals: 1) to explore and further specify what parts of Nozicks theory meani.e., what determines peoples judgements of closeness, 2) to show that Nozick's theory is a framework is a useful framework for describing how people think about personality identity and, 3) to use Nozicks framework to resolve existing issues in the personal identity literature. While I think the design of the experiments and results seems to address the first goal, the support for the second and third goals would benefit from further support/clarification.
Regarding the second goal (to show that the closest continuer theory is a useful framework for describing how people think about personal identity), it seems like there are a number of areas where the results are counter to what the theory would predict or the relevant results are not highlighted. For example, the violation of transitivity seems to be in direct contradiction to the theory. Further, a key aspect of this theory seems to be that there is a separation between the continuer judgment and the closeness judgment but its not entirely clear to me where the separation in these types of judgments is shown in the participants responses.

Response: Nozick's theory is a normative theory in the philosophy of personal identity. Thank your for your assessment regarding the first goal. Prima facie, a violation of premises or implications by laypeople would not invalidate the theory, but you are absolutely right in being concerned that it might limits its use. One can argue that the (empirical) usefulness of a theory could be judged in its ability to organize empirical findings. This is the standard defense of rational choice theory (that is no less a normative, not an empirical theory) against empirical violations. We nonetheless agree that it is important to investigate deviations, and first step for this investigation is to identify both the nature and the extent of these deviations. Thus, we attempted to stress congruence between theory and results and divergence. We discuss the role of intransivity in the discussion.
We also agree with your statement about the third goal, but we would see this part of our contribution as mainly theoretical. We explain that the empirical findings in areas of the social sciences (subsection "The crisis of identity") are not able to address

the guiding questions, but instead of discarding those findings as irrelevant for personal identity (e.g. Starmans & Bloom, 2018), we assign a constructive and essential place to these investigations (subsection "Separating closeness from identity"). This part of our contribution would not be invalidated by a failure of the second part, as this would not single out our findings as uniquely problematic.
We agree that we cannot give a full response to the second goal raised. We have not developed a full empirical process model of the reidentification decision, but we have made at least some progress. We have added a new subsection to the discussion in which we discuss desiderata for future research to continue this project (shown in the Appendix in this response letter).

R4.4: The suggestion that the open-ended responses suggest that people follow the logic of the closest continuer theory needs further discussion. It appears that the evidence for this is that people tend to justify their allocation decisions with some statement about giving more money to the person who is more of them or closest to them. However, this seems like it could be consistent with a view of personality identity as similarity. It seems reasonable that "closest to me" or "more of me" could mean that people see that person as more similar and that people weight psychology and memory more heavily in similarity judgments than the other metrics used in these studies. Further, I would have thought that "logic" of the closest continuer theory would be more about the decision tree (and the separation between continuer and closeness judgments) rather than about just closeness alone as an input into allocation decisions. Perhaps this is addressed by the cluster analysis in the SM but its not clear to me which of those clusters correspond to those expected in Nozicks decision scheme. (As a result, Im not sure what the statement "most responses can be interpreted meaningfully through the lens of the closest continuer theory" (pg. 19) refers to.

Response: We discusss the relation between similarity and closeness in our response to point R4.7. Based on this understanding, there is no incompatiblity between your reading of the phrases "closest to me" and "more of me" and the assumptions of the closest continuer theory. You are correct in pointing out the difference between closeness assessment and reidentification. This is also our main concern about many of the studies that purportedly investigate personal identity and, in our reading, are focused on similarity, instead (Starmans & Bloom, 2018). Thus, the postquestionnaire questions address the possible outcomes of Nozick's decision tree. The sentence you cite follows several references to the supporting material. As stated in our response to R4.2, we decided to move these analyses into the supplement to reduce the length of the main manuscript. Here we explore the relationship between feature distribution and judgments of non-existence, for example. The downside of this decision is that some statements cannot be verified by results in the main text alone. The new passage (see the Appendix) formulates further steps that could be taken to further explore the decision logic.

R4.5: In general, as it is a complex theory and there are a large number of results, I think itd be very helpful to, upfront, more clearly lay out the parts of the theory that are being tested and what pattern of results would support the theory and what pattern would be contradictory to the theory. Of course, the manuscript does lay out some of the major hypotheses and research questions at the beginning, however, some prioritization (or, perhaps, statement about what is not the focus of the manuscript) would be useful as to help focus the reader on the main takeaways.

Response: Nozick's theory is a normative theory that stands independent of empirical findings. One could criticize its assumptions or show discrepancies between common understandings and premises in this theory. Thus, the first part of our manuscript lays out the theoretical framework. We are not able to address every aspect and implication of this theory in our empirical investigation. Thus, we develop our hypotheses and research questions to specify the focus of our attention. Our hypotheses are derived from the philosophical and psychologcial literature and not restricted to Nozick's theory. Thus, when we find that possessions are disregarded by participants (Hypothesis 1e), we do not gain evidence against Nozick's theory, but evidence against William James's conviction that possessions have a central place in self-conceptions that have been taken up by prominet theories in marketing (Belk, 1988). Thus, we decided to guide our investigation of lay-theories of personal identity by research questions, not hypotheses. This investigation cannot provide evidence against the framework, but we can test to which degree the framework can help to organize empirical findings. We believe that this distinction should indeed be mentioned at the beginning of the section on research questions and hypotheses, and have included clarifying passages. In the section "Dimensions of assessing closeness", we added:

> Note that we do not derive these features from Nozick's theory, as the closeness metric could accommodate all or none of them. Thus, our hypotheses are derived from the cited theories in philosophy and psychology.

and we added a passage to our introduction to "Personal reidentification after fission":

> Of course, no empirical finding could be interpreted as a refutation of Nozick's normative theory. The analysis will thus focus on the usefulness of this framework for organizing empirical responses by (mostly) non-philosophers. Thus, we formulate research questions, and not hypotheses.

R4.6: Regarding the third goal, it seems like the suggestion is that Nozicks framework is useful in resolving issues in the personal identity literature because it separates closeness and continuity (identity), and avoids the issue of studying similarity rather than identity. As noted above, its not entirely clear to me where the separation between closeness and continuity is shown in the results. It seems likely that there is data to show this but it either isnt explicitly stated or is buried in a lot of other results.

Response: Please see our response to R4.3. An important part of our contribution is theoretical in nature: None of the criticized paradigms would even be able to identify discrepancies between normative theories and lay-perspectives. The usefulness of the framework lies in its capability to integrate data generated in these studies irrespective of this shortcoming, that led other to postulate that these studies should be disregarded for addressing questions of personal identity.
Thus, our paradigm overlaps to some degree with previous studies in collecting assessments of continuity/similarity, but it goes further and also analyzes the decision-making process that requires theses assessments as input.

R4.7: I think the definition of similarity that is being used in this manuscript could be more explicitly stated as the difference between similarity and closeness is not entirely clear. One difference between closeness and similarity seems to be that closeness allows for different dimensions to have different weights and for the weights to vary across individuals. However, some accounts of similarity would allow for such differences in weights across features and across individuals (e.g., Ahn et al., 2000 Cognitive Psychology). If similarity is intended to mean simple feature overlap, since the "features" used in the study are such large categories (that may include differing numbers of features), its a bit unclear to me that the experiments can differentiate closeness from a feature-overlap similarity.

Response: We would consider similarity judgments to be a wider class than closeness judgments in the context of personal identity. We completely agree that similarity judgment can vary inter-individually and are modeled as such (e.g., Nosofsky, 1991). At the same time, feature overlap does not seem to capture the way factors influenced participants' judgments. Note that in all versions of the scenarios all features were preserved, only in different compositions and distributed across two continuers. Similarly, the effect of splitting memory and psychology on identity judgments was shown to appear independent of the number of features preserved in the continuers (see also SM, Figure S7). Closenes is obviously a type of similarity: both have a maximum value in numeric identity (when even spatial co-ordinates are shared). But mere qualitative identity would not be enough to constate numerical identity, as the introductory example about billiard balls underlines.

R4.8: Minor Comments:

It would be very helpful to restate what the research questions are when they are referred to in the results section by numberits very hard to remember what question each number refers to. The hypotheses are re-stated more often, however, the same comment applies when they are only referred to by number.

Response: Thank you for this suggestion. We went through the manuscript and identified references to hypotheses and research questions. We completely agree that—especially

the research questions—might not have been evident in the context of our references. We thus extended the sentences with these reference resulting in the sentences:

> "we can give an affirmative answer to Research Question 1: The importance assigned to each of the five dimensions was similar for identity and survival"

and

> "The overall pattern indicates a positive answer to Research Question 2: Participants explain their monetary allocation often in reference to closeness or identity relations with continuers."

Research Question 3 and 4 are now repeated at the beginning of the results paragraph, and we added reminders to the dimensions specified by the hypotheses in brackets after these references.

References

Belk, R. W. (1988). Possessions and the extended self. *Journal of Consumer Research*, *15*(2), 139–168. doi: 10.1086/209154

Brandstätter, E., Gigerenzer, G., & Hertwig, R. (2006). The priority heuristic: Making choices without trade-offs. *Psychological Review*, *113*(2), 409–432. doi: 10.1037/0033-295X.113.2.409

Brook, A. (2014). Tracking a person over time is tracking what? *Topics in Cognitive Science*, *6*(4), 585–598. doi: 10.1111/tops.12107

Brueckner, A. (1993). Parfit on what matters in survival. *Philosophical Studies*, *70*(1), 1–22. doi: 10.1007/bf00989659

Bullot, N. J. (2014). Explaining person identification: An inquiry into the tracking of human agents. *Topics in Cognitive Science*, *6*(4), 567–584. doi: 10.1111/tops.12109

Campbell, S. (2005). Is causation necessary for what matters in survival? *Philosophical Studies*, *126*(3), 375–396. doi: 10.1007/s11098-004-7786-1

Cokely, E. T., & Feltz, A. (2009). Adaptive variation in judgment and philosophical intuition. *Consciousness and Cognition*, *18*(1), 356–358. doi: 10.1016/j.concog.2009.01.001

Feltz, A., & Cokely, E. T. (2009). Do judgments about freedom and responsibility depend on who you are? Personality differences in intuitions about compatibilism and incompatibilism. *Consciousness and Cognition*, *18*(1), 342–350. doi: 10.1016/j.concog.2008.08.001

Fields, C. (2012). The very same thing: Extending the object token concept to incorporate causal constraints on individual identity. *Advances in Cognitive Psychology*, *8*(3), 234–247. doi: 10.2478/v10053-008-0119-8

Gigerenzer, G. (1996). On narrow norms and vague heuristics: A reply to Kahneman and Tversky. *Psychological Review*, *103*(3), 592–596. doi: 10.1037/0033-295X.103.3.592

Gutheil, G., Gelman, S. A., Klein, E., Michos, K., & Kelaita, K. (2008). Preschoolers' use of spatiotemporal history, appearance, and proper name in determining individual identity. *Cognition*, *107*(1), 366–380. doi: 10.1016/j.cognition.2007.07.014

Heiphetz, L., Strohminger, N., Gelman, S. A., & Young, L. L. (2018). Who am I? the role of moral beliefs in children's and adults' understanding of identity. *Journal of Experimental Social Psychology*, *78*, 210–219. doi: 10.1016/j.jesp.2018.03.007

Heiphetz, L., Strohminger, N., & Young, L. L. (2017). The role of moral beliefs, memories, and preferences in representations of identity. *Cognitive Science*, *41*(3), 744–767. doi: 10.1111/cogs.12354

James, W. (1890). *The principles of psychology (Vol. 1).* New York, NY: Holt.

Johansson, J. (2010). Parfit on fission. *Philosophical Studies*, *150*(1), 21–35. doi: 10.1007/s11098-009-9393-7

Johnson, J. L. (1997). Personal survival and the closest-continuer theory. *International Journal for Philosophy of Religion*, *41*(1), 13–23.

Kolak, D., & Martin, R. (1987). Personal identity and causality: Becoming unglued. *American Philosophical Quarterly*, *24*(4), 339–347.

Landy, J., Jia, M., Ding, I., Viganola, D., Tierney, W., Dreber, A., … others (2019). Crowdsourcing hypothesis tests: Making transparent how design choices shape research results. *Psychological Bulletin*.

Martignon, L., Katsikopoulos, K. V., & Woike, J. K. (2008). Categorization with limited resources: A family of simple heuristics. *Journal of Mathematical Psychology*, *52*(6), 352–361. doi: 10.1016/j.jmp.2008.04.003

Matthews, S. (2000). Survival and separation. *Philosophical Studies*, *98*(3), 279–303. doi: 10.1023/A:10186611

Mills, E. (1993). Dividing without reducing: Bodily fission and personal identity. *Mind*, *102*(405), 37–51. doi: 10.1093/mind/102.405.37

Moyer, M. (2008). A survival guide to fission. *Philosophical Studies*, *141*(3), 299–322. doi: 10.1007/s11098-007-9161-5

Newman, G. E., De Freitas, J., & Knobe, J. (2015). Beliefs about the true self explain asymmetries based on moral judgment. *Cognitive Science*, *39*(1), 96–125. doi: 10.1111/cogs.12134

Nichols, S., & Bruno, M. (2010). Intuitions about personal identity: An empirical study. *Philosophical Psychology*, *23*(3), 293–312. doi: 10.1080/09515089.2010.490939

Nosofsky, R. M. (1991). Relation between the rational model and the context model of categorization. *Psychological Science*, *2*(6), 416–421.

Nozick, R. (1981). *Philosophical explanations.* Cambridge, MA: Belknap.

Nunner-Winkler, G. (2015). Personal identity: Philosophical aspects. In J. D. Wright (Ed.), *International encyclopedia of the social & behavioral sciences* (2nd ed., pp. 742–749). Oxford, UK: Elsevier.

Parfit, D. (1984). *Reasons and persons.* Oxford, UK: Oxford University Press.

Perry, J. (1972). Can the self divide? *The Journal of Philosophy*, *69*(16), 463–488. doi: 10.2307/2025324

Prinz, J. J., & Nichols, S. (2016). Diachronic identity and the moral self. In *The routledge handbook of philosophy of the social mind* (pp. 465–480). Routledge.

Rachels, S., & Alter, T. (2005). Nothing matters in survival. *The Journal of Ethics*, *9*(3-4), 311–330. doi: 10.1007/s10892-005-3506-0

Rips, L. J., Blok, S., & Newman, G. (2006). Tracing the identity of objects. *Psychological Review*, *113*(1), 1–30. doi: 10.1037/0033-295x.113.1.1

Rorty, A. O. (1973). The transformations of persons. *Philosophy*, *48*(185), 261–275. doi: 10.1017/s0031819100042753

Sagi, E., & Rips, L. J. (2014). Identity, causality, and pronoun ambiguity. *Topics in Cognitive Science*, *6*(4), 663–680. doi: 10.1111/tops.12105

Shoemaker, D. (2014). Personal identity and ethics. In E. N. Zalta (Ed.), *The Stanford encyclopedia of philosophy* (Spring 2014 ed.). `http://plato.stanford.edu/archives/spr2014/entries/identity-ethics/`.

Shoemaker, D. W. (2007). Personal identity and practical concerns. *Mind*, *116*(462), 317–357. doi: 10.1093/mind/fzm317

Shoemaker, S. (1990). First-person access. *Philosophical Perspectives*, *4*, 187–214. doi: 10.2307/2214192

Shoemaker, S. (1994). The first-person perspective. *Proceedings and Addresses of the American Philosophical Association*, *68*(2), 7–22. doi: 10.2307/3130588

Sider, T. (2001). Criteria of personal identity and the limits of conceptual analysis. *Noûs*, *35*(s15), 189–209. doi: 10.1111/0029-4624.35.s15.10

Starmans, C., & Bloom, P. (2018). Nothing personal: What psychologists get wrong about identity. *Trends in Cognitive Sciences*. doi: https://doi.org/10.1016/j.tics.2018.04.002

Stevens, J. R., Woike, J. K., Schooler, L. J., Lindner, S., & Pachur, T. (2018). Social contact patterns can buffer costs of forgetting in the evolution of cooperation. *Proceedings of the Royal Society B: Biological Sciences*, *285*(1880), 20180407.

Strohminger, N., & Nichols, S. (2014). The essential moral self. *Cognition*, *131*(1), 159–171. doi: 10.1016/j.cognition.2013.12.005

Strohminger, N., & Nichols, S. (2015). Neurodegeneration and identity. *Psychological Science*, *26*(9), 1469–1479. doi: 10.1177/0956797615592381

Wagner, L., & Carey, S. (2003). Individuation of objects and events: A developmental study. *Cognition*, *90*(2), 163–191. doi: 10.1016/s0010-0277(03)00143-4

White, C. (2015). Establishing personal identity in reincarnation: Minds and bodies reconsidered. *Journal of Cognition and Culture*, *15*(3-4), 402–429. doi: 10.1163/15685373-12342158

White, C. M., Hafenbrädl, S., Hoffrage, U., Reisen, N., & Woike, J. K. (2011). Are groups more likely to defer choice than their members? *Judgment and Decision Making*, *6*(3), 239.

White, C. M., & Hoffrage, U. (2009). Testing the tyranny of too much choice against the allure of more choice. *Psychology & Marketing*, *26*(3), 280–298. doi: 10.1002/mar.20273

Williams, B. (1970). The self and the future. *The Philosophical Review*, *79*(2), 161–180. doi: 10.2307/2183946

Woike, J. K. (2019). *Replication Data for: "Putting your Money Where your Self is"*. Harvard Dataverse. Retrieved from https://doi.org/10.7910/DVN/AN1QF3 doi: 10.7910/DVN/AN1QF3

Woike, J. K., Hoffrage, U., & Martignon, L. (2017). Integrating and testing natural frequencies, naïve Bayes, and fast and frugal trees. *Decision*, *4*(4), 234–260. doi: 10.1037/dec0000086

6 Appendix A: New discussion section

Towards a process model of re-identification

Our studies allow to make some progress in the analysis of decisions involved in determining personal identity. Like Rips et al. (2006), who develop the causal continuer model based on Nozick's theory, we are interested in the decision process. Decision processes, as implemented by human beings, are often insufficiently described by functions merely predicting decision outcomes. A further analysis of the decision processes needs to address questions of information search: Which persons are considered as continuers? When and why is the search for possible continuers stopped? Which dimensions are considered in the subjective closeness metric, and how are these dimensions integrated? We showed some results compatible with decision-making following the closet continuer logic. Is there further evidence for the three steps being followed in a specific sequence[2] and how stable is this process across individuals? A structurally similar model of decision making has been proposed for explaining the phenomenon of choice deferral (C. M. White, Hafenbrädl, Hoffrage, Reisen, & Woike, 2011; C. M. White & Hoffrage, 2009). When faced with a selection of possible alternatives, choice in the 2S2T-model (C. M. White & Hoffrage, 2009) is deferred for one of two reasons. First, none of the options is good enough and surpasses a decision threshold or second, too many options are good enough, surpassing the threshold but it becomes difficult to choose the best option. Of course, personal re-identification is not simply preferential choice but the analysis of the decision process might still be informed by the analysis of related or parallel processes in other domains.

While the mathematical form of weighted-additive linear models implies weighting and adding, many other operations, such as lexicographic stepwise procedures that ignore (sometimes most of the) variables in the equation (Woike, Hoffrage, & Martignon, 2017) would still be captured by this model (Martignon, Katsikopoulos, & Woike, 2008). Brook (2014) argued for a model of personal re-identification that starts with psychological factors and only considered other dimensions if the information is missing (or inconclusive). Variance in choosing and applying criteria might again be related to other individual differences (Cokely & Feltz, 2009; Feltz & Cokely, 2009). Based on the variations in our chosen design for this study, it is not yet possible to build cognitive models of participants' decisions. It is, for example, unclear whether an appropriate model should be stochastic, as in Rips et al. (2006), or deterministic.

Our scenarios varied factors that should mostly influence the assessment of closeness and only indirectly the decision-making based on these assessments. Future research could shift this focus to the subsequent stages of the procedure. Thus, specific exit nodes of the decision tree in Figure 1 could be investigated. For example, is there a minimum level of closeness required for participants to determine that any of the continuers is identical to the original person? Do participants share the intuition that a fission resulting in multiple exact copies does not preserve identity, and would this depend on the level of closeness? What type of difference is considered to be sufficient to single out a closest continuer?

[2]The fast-and-frugal tree in Figure 1 would not yield different outcomes if the first three levels changed their relative position.

Both studies in this manuscript confronted participants with two continuers. Future studies could increase the number of continuers. A different approach could focus on one continuer by either keeping a second continuer constant, or moving from paired comparisons to binary reidentification. Previous studies have explored variants of thought experiments compatible with these ideas. C. White (2015) implemented one such scenario, focusing on the likelihood that a living person might be the reincarnation of a deceased person (see also (Strohminger & Nichols, 2014)). For reincarnation judgments, distinctiveness was found to guide decisions. Similar to our sci-fi scenario, this setting might introduce specific assumptions about the process of reincarnation that could guide responses. For example, the importance of body similarity might be evaluated to be a lot lower than when responding to a scenario, in which a ship wreck survivor is returned from an island and matched to missing persons, and the importance of moral attributes to be higher. In contrast to the second scenario, the reincarnation scenario prevents the use of causal histories that are useful for person tracking (Bullot, 2014).

It might also be the case that different practical concerns demand different criteria of identity. We investigated the parameters of identity in the context of re-identification and compensation. Other practical concerns, such as attributing blame, responsibility, or guilt, or allocating punishments and rewards, might trigger different responses, as the criteria of identity might shift or current properties of persons might become more relevant than historical properties and re-identification questions (D. W. Shoemaker, 2007; D. Shoemaker, 2014).

To sum up, while we made progress to shed light on the decision processes used by participants, we have not yet established a complete process model, which should be the goal for future research (Brandstätter, Gigerenzer, & Hertwig, 2006; Gigerenzer, 1996).
